# Intraspecies characterization of bacteria via evolutionary modeling of protein domains

**DOI:** 10.1038/s41598-022-21036-3

**Published:** 2022-10-05

**Authors:** Iva Budimir, Enrico Giampieri, Edoardo Saccenti, Maria Suarez-Diez, Martina Tarozzi, Daniele Dall’Olio, Alessandra Merlotti, Nico Curti, Daniel Remondini, Gastone Castellani, Claudia Sala

**Affiliations:** 1 Department of Physics and Astronomy ‘Augusto Righi’, University of Bologna, 40127 Bologna, Italy; 2 Department of Experimental, Diagnostic and Specialty Medicine, University of Bologna, 40138 Bologna, Italy; 3 Laboratory of Systems and Synthetic Biology, Wageningen University and Research, 6708 WE Wageningen, The Netherlands

**Keywords:** Computational biology and bioinformatics, Classification and taxonomy, Phylogeny, Ecological modelling

## Abstract

The ability to detect and characterize bacteria within a biological sample is crucial for the monitoring of infections and epidemics, as well as for the study of human health and its relationship with commensal microorganisms. To this aim, a commonly used technique is the 16S rRNA gene targeted sequencing. PCR-amplified 16S sequences derived from the sample of interest are usually clustered into the so-called Operational Taxonomic Units (OTUs) based on pairwise similarities. Then, representative OTU sequences are compared with reference (human-made) databases to derive their phylogeny and taxonomic classification. Here, we propose a new reference-free approach to define the phylogenetic distance between bacteria based on protein domains, which are the evolving units of proteins. We extract the protein domain profiles of 3368 bacterial genomes and we use an ecological approach to model their Relative Species Abundance distribution. Based on the model parameters, we then derive a new measurement of phylogenetic distance. Finally, we show that such model-based distance is capable of detecting differences between bacteria in cases in which the 16S rRNA-based method fails, providing a possibly complementary approach , which is particularly promising for the analysis of bacterial populations measured by shotgun sequencing.

## Introduction

The accurate identification and classification of bacteria is essential for epidemiological surveillance, as well as for the study and understanding of specific ecosystems such as the human microbiome. Bacterial classification, however, is a complex problem, especially at the intraspecies level. Commonly used methods include the reconstruction of phylogenetic trees based on the comparison of specific molecular sequences (e.g. 16S rRNA gene) with reference databases, which contain annotated sequences of previously classified bacteria . This allows to generate the so-called taxonomic classification of the sampled bacteria.

The basic unit of classification in taxonomy is usually the species level. While such resolution may be sufficient for other domains of life, the intraspecies level of bacteria includes high amounts of genomic and phenot ypic variation, through which strains of bacteria belonging to the same species manifest highly different behaviours^1^. For instance, different strains can infect different hosts^2–4^, or even more extremely, some bacterial species can have both pathogenic and non-pathogenic isolates^1,5^. The 16S rRNA gene is commonly used for bacterial classification since random mutations of the 16S rRNA gene sequence serve as a proxy of evolutionary time^6^. However, bacterial classification based on 16S rRNA gene has low phylogenetic power at the intraspecies level, where functional diversification of strains is faster than random mutations of the 16S rRNA gene^6^. Various alternative approaches to estimate the bacterial phylogeny have hence been proposed^7–9^. Such methods mainly start from specific bacterial molecular sequences and either compute a distance matrix or model the substitution process through which the molecular sequences have evolved. In both cases, the appropriate choice of the specific molecular sequences to be investigated is crucial. The chosen sequences have to satisfy two conditions for the phylogenetic reconstruction to be successful: orthologs of sequences have to be shared by the tested bacteria and they have to contain a vertical phylogenetic signal. Approaches that use a single molecular sequence, such as 16S rRNA, may work well at higher taxonomic levels, but they are usually too generic to differentiate species or strains. A popular method which overcomes this problem while still controlling the phylogenetic noise is multilocus sequence analysis (MLSA)^10,11^, which simultaneously uses sequences of seven housekeeping genes. However, phylogenetic signals are not equally distributed across the genome and, while some classes of genes are expected to perform better for phylogenetic inference than others^12^, the choice of the specific set of genes to be used is still a matter of debate.

Here, we develop a new measurement of evolutionary distance derived from the ecological modeling of the protein domains that populate the bacterial genome and we propose to exploit such distance to deepen the characterization of bacteria at the intraspecies level. Our method does not rely on the selection of a specific set of genes or molecular sequences, but is based on the ecological modelling of the whole set of protein domains within the bacterial genome. Protein domains are segments of proteins and are defined as substructures produced by any contiguous part of a polypeptide chain that can fold independently of the rest of the protein into a compact and stable structure^13,14^. Studies on the conformation, function and evolution of proteins suggest to consider the protein as a molecule composed of a set of independent protein domains, which constitute its organizational units, so as its evolutionary components. The evolution of living organisms, or rather, the proteome evolution, is caused by the occurrence of random modifications of the genetic code and these events affect each protein domain independently. Protein domain duplication, mutation, recombination, relocation and horizontal transfer are in fact the main mechanisms that allow the outbreak of proteins with new functionalities, thereby contributing to the emergence of complexity^15–17^. As a matter of fact, the relationship between protein domain evolution and phylogeny has already been investigated in previous works, with findings suggesting a connection between the two. On the one hand the phylogeny of hundreds of organisms (including more than 100 bacteria) has been reconstructed satisfactorily (i.e., in good agreement with the classical taxonomy) based on the presence or absence of protein domain superfamilies^18^, or based on specific patterns of protein domains^19^. On the other hand the evolutionary origin and history of protein domains has been reconstructed based on the taxonomic tree of organisms^20^.

Taking an ecological approach, we here focus on the dynamic processes that led to the current configuration of protein domains, assuming that this could highlight important aspects of the evolution of genomes, proteins, and organisms. The proposed phylogeny will then be based on the evolutionary model of protein domains, incorporating the parameters estimated from such model.

### Evolutionary modeling of protein domains

In the past, various models of evolution have been proposed for protein domains, each linked to a particular distribution of the protein domains frequencies. This distribution is what in ecology is called Relative Species Abundance distribution (RSA). It is a measure of the biodiversity of an ecosystem, in our case the set of protein domain families that populate the genome, and can be used to fit real data in order to verify the validity of the underlying ecological hypothesis. Among the proposed ecological models for protein domains, the birth-death-innovation one stands out. The RSA distribution predicted by this model is a Pareto distribution and it fits the RSA of a wide range of genome-associated quantities well, including protein domains^21,22^.

Here, we consider a generalization of the birth-death-innovation model^21^ in which both demographic and environmental noise are added to the model. Environmental stochasticity is due to environmental changes which act simultaneously on all individuals while demographic stochasticity reflects the reproduction differences among individuals within a population. A suitable generalized model was developed by S. Engen and R. Lande in 1996^23,24^, who proposed that the RSA distribution could be a Poisson Log-Normal or a Negative Binomial, depending on the assumptions.

Following Engen and Lande’s derivation^23,24^, we make the neutral ecological equivalence assumption that the evolutionary dynamics is the same for all the protein domain families. In this way, we will not need to introduce protein domain family-specific indices for the evolutionary rates in the following equations^25^. As in Engen and Lande’s papers, we also assume that protein domain families enter the protein domain community (i.e. the genome) at times generated by an inhomogeneous Poisson process (see Supplementary Material or, for more details, Karlin and Taylor textbooks^26,27^) with rate $\omega (t)$ and that they evolve independently of each other. Denoting the present time as $t=0$, speciations (i.e. arrivals of new protein domain families in the genome) up to now have then occurred at negative times, generated by $\omega (t)$. Let the distribution of the abundance *X*(*t*) at time $t=0$ of a protein domain family entering the genome at time $-t < 0$ be *f*(*x*; *t*) and let *p*(*t*) be the probability that it has not gone extinct. Then, Engen and Lande showed that the abundances *x* of the protein domain families in the community are generated by an inhomogeneous Poisson process with rate

1 $$\begin{aligned} \lambda (x) = \int _0^{\infty } \omega (-t) p(t) f(x;t) dt, \end{aligned}$$

Now, we let the process for each protein domain family be a diffusion process (see Supplementary Material) which is a solution of the stochastic growth equation

2 $$\begin{aligned} \frac{dx}{dt} = rx - xg(x) + x\sigma _r(x)\frac{dB(t)}{dt}, \end{aligned}$$

where *x* still indicates the number of individuals within a protein domain family (i.e., its abundance), *r* is the constant growth rate ($r = b-d$, where *b* is the birth rate and *d* is the death rate), *g*(*x*) is a density regulation function, $\sigma ^2_r = \sigma _e^2 + \sigma _d^2/x$ is the sum of the environmental ($\sigma _e^2$) and demographic ($\sigma _d^2/x$) stochasticities, and the process *B*(*t*) is a standard Brownian motion, so that $\frac{dB(t)}{dt}$ is the so-called white noise. It can be shown (see Supplementary Material) that the diffusion process which solves the stochastic differential Eq. (2) has infinitesimal mean $m(x) = [r+\sigma ^2_d/(2x)+\sigma ^2_e/2]x - xg(x)$ which can be rewritten as $m(x)=[r-g(x)]x + (1/2)\sigma ^2_d + (1/2)\sigma ^2_ex$. The noise term hence turns out to be composed by a multiplicative part (i.e., the environmental noise, which acts on the whole population of family domains), and an additive one (i.e., the demographic noise, which acts on individuals within the population and whose effect is thus stronger in smaller populations).

If protein domain families evolve independently from each other, then Engen and Lande showed that their abundances are generated by an inhomogeneous Poisson process with rate $\lambda (x)$ (see Supplementary Material). Specifically, depending on which assumptions are made on the model parameters, the predicted RSA distribution could be a Poisson Log-Normal, a Negative Binomial, or a Log-Series distribution.

Engen and Lande showed that if we consider the Gompertzian density regulation function^23,28^ $g(x)=\gamma \ln (x+\epsilon )$ , where $\gamma$ is a constant and $\epsilon =\sigma _d^2/\sigma _e^2$, then

3 $$\begin{aligned} \lambda (x) = \frac{\alpha \omega _0}{x+\epsilon } e^{-\frac{1}{2}\frac{[ln(x+\epsilon ) -r/\gamma ]^2}{\sigma _e^2/2\gamma }} \end{aligned}$$

where $\omega _0$ is the speciation parameter and $\alpha = \frac{2}{\sigma _e^2}e^{\frac{\gamma }{\sigma _e^2}\left[ ln(1+\epsilon ) -\frac{r}{\gamma } \right] ^2}$. When $\epsilon \ll x$, $\lambda (x)$ can be approximated to a Log-Normal distribution

4 $$\begin{aligned} \text {Log-Normal}(x) = \frac{1}{x \sigma \sqrt{2 \pi }} e^{-\frac{(ln(x)-\mu )^2}{2 \sigma ^2}}, \end{aligned}$$

with parameters $\mu = r/\gamma$ and $\sigma ^2 = \sigma ^2_{e}/2 \gamma$. This implies that the RSA distribution of protein domains would be a Poisson Log-Normal with parameters $\mu$ and $\sigma ^2$.

If instead, we consider a linear density regulation function^24^ $g(x)=\eta x$, where $\eta$ is a constant, then

5 $$\begin{aligned} \lambda (x) = a \omega _0 (x+\epsilon )^{\frac{2(r+\eta \epsilon )}{\sigma ^2_e}-1} e^{-\frac{2\eta }{\sigma ^2_e}(x+\epsilon )}, \end{aligned}$$

where $\omega _0$ is the speciation parameter, $\epsilon =\sigma _d^2/\sigma _e^2$ and $a = \frac{2 e^{2(1+\epsilon )\eta /\sigma ^2_e}}{\sigma ^2_e(1+\epsilon )^{2(r+\eta \epsilon )/\sigma ^2_e}}$. When $\epsilon \ll x$, $\lambda (x)$ is now a Gamma distribution with shape $\alpha =2(r+\eta \epsilon )/\sigma ^2_e$ and rate $\beta =2\eta /\sigma ^2_e$, and the RSA distribution of protein domains is a Negative Binomial

6 $$\begin{aligned} \text {Negative Binomial}(x) = \frac{\Gamma (x+\alpha )!}{x!\Gamma (\alpha )!} (1- {q})^{\alpha } {q}^x, \end{aligned}$$

with dispersion $\alpha$ and success rate ${q}=\sigma ^2_e/(2\eta + \sigma ^2_e)$.

Finally, if we consider the limit of Eq. (6) in which the dispersion parameter $\alpha$ tends to zero, the Negative Binomial density function [Eq. (6)] converges to a Log-Series

7 $$\begin{aligned} \text {Log-Series}(x) = \frac{{q}^x}{x}, \end{aligned}$$

that hence represents a special case of Negative Binomial model when $2(r+\eta \epsilon )/\sigma ^2_e \rightarrow 0$.

Here, we aim to model the evolution of protein domains within the genome using Engen and Lande’s theoretical framework. Specifically, modeling the protein domain RSA of thousands of bacterial genomes, we intend to provide a novel phylogenetic characterization of bacteria.

We hypothesize that new protein domain families are generated in the genome at times specified by an inhomogeneous Poisson process and that different families are mutually independent. Within the protein domain families, we assume that the evolutionary dynamics can be described by a stochastic birth-death process in which: (i) the growth rate *r* is constant; (ii) both environmental and demographic noise are included; iii) density regulation function is either linear or Gompertzian. The growth rate *r* can be interpreted as the rate by which a protein domain family gets a new copy inside the genome. This unifies two different events, the birth and death of protein domains which correspond to protein domain duplication and protein domain inactivation/deletion. Regarding the two noise terms ($\sigma _d^2$ and $\sigma _e^2$), the demographic variance $\sigma _d^2$ captures small differences in duplication or survival between different individual domains which belong to the same protein domain family, while the environmental variance $\sigma _e^2$ represents larger scale disruptions of the proteome which act on all protein domains within the family simultaneously. Events such as horizontal gene transfer are an example of environmental stochasticity. The role of the density regulation, *g*(*x*), is to keep the abundance of protein domain family from diverging. This is usually a consequence of the limited availability of resources in the community, and it can be related to the limited size of the genome (and consequently of the proteome) in the contest of protein domains^29^.

The modeling of protein domain RSA within bacterial genomes will allow us to derive a novel measurement of phylogenetic distance. Specifically, we will show that the Poisson Log-Normal distribution [Eq. (4)] is the one that best fits the empirical RSA of bacterial protein domain families and we will derive our novel phylogenetic distance based on its two parameters $\mu = r/\gamma$ and $\sigma ^2 = \sigma ^2_{e}/2 \gamma$, together with the protein domains density within the bacterial genome, as detailed in the "Materials and methods" section. In the following, we will show how such distance is related to the usual 16S rRNA gene-based and taxonomic classifications of bacteria and how it constitutes a promising measurement to investigate the bacterial phylogeny at the intraspecies level, where both 16S rRNA and taxonomic classification fail.

## Results and discussion

### Protein domains show a Gompertzian growth

The protein domain RSA distributions of 3368 bacterial genomes were obtained as detailed in the "Materials and methods" section. Briefly, for each bacterial genome we retrieved all the identifiable protein domains. Then, we computed the RSA by counting the number of protein domains belonging to each protein domain family.

**Figure 1:**
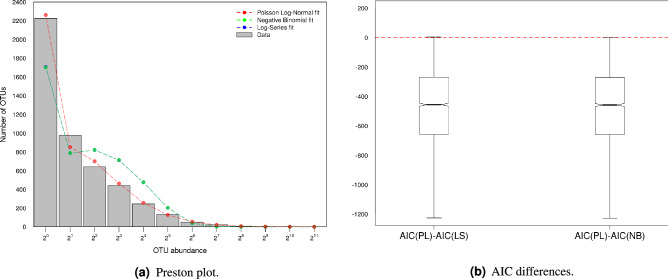
Fit of protein domains RSA. (**a**) Example of protein domains Preston plot fitted with three different distributions: the Poisson Log-Normal, the Negative Binomial and the Log-Series. Results refer to the bacterial genome $\text {GCA}\_000717515$. The Negative Binomial and the Log-Series fit overlap. This implies that the dispersion parameter $\alpha$ of the Negative Binomial distribution (see Eq. (6)) is close to zero. The mean and the median of the dispersion parameter obtained for the 3368 bacterial genomes are ${2.67\times 10^{-4}}$ and ${2.62\times 10^{-7}}$, in agreement with the observed overlap. (**b**) Distribution of the difference between the AIC obtained with the Poisson Log-Normal model (PL) and the Log-Series (LS) or the Negative Binomial (NB) model, considering all the 3368 bacterial genomes.

Three evolutionary hypotheses were tested by fitting the empirical RSAs with the Log-Series [Eq. (7)], the Negative Binomial (Eq. (6)) and the Poisson Log-Normal (Eq. (4)) distribution (Fig. 1a). According to the Akaike Information Criterion (AIC)^30^, in $99.97\%$ of bacteria the selected model was the Poisson Log-Normal (Fig. 1b). This model performed better than both the Log-Series and the Negative Binomial and described the data well, with an average $R^2$ of 0.97 (minimum $R^2$=0.86). The selection of the Poisson Log-Normal model instead of the Negative Binomial or the Log-Series, implies that the protein domains evolution process is characterized by a Gompertzian density regulation function ($g(x)=\gamma \ln (x+\epsilon )$) rather than a linear one ($g(x)=\eta x$). This suggests an asymmetric process in which the proliferation rate for low abundant protein domains is faster than for the high abundant ones.

### Protein domains deactivation is faster than duplication

The examination of the Poisson Log-Normal scale ($\mu$) and location ($\sigma ^2$) parameters (see Eq. (4) and Supplementary Material) estimated by the fitting procedure for each bacterial genome, allows us to reveal further features of the evolutionary process of protein domains.

**Figure 2:**
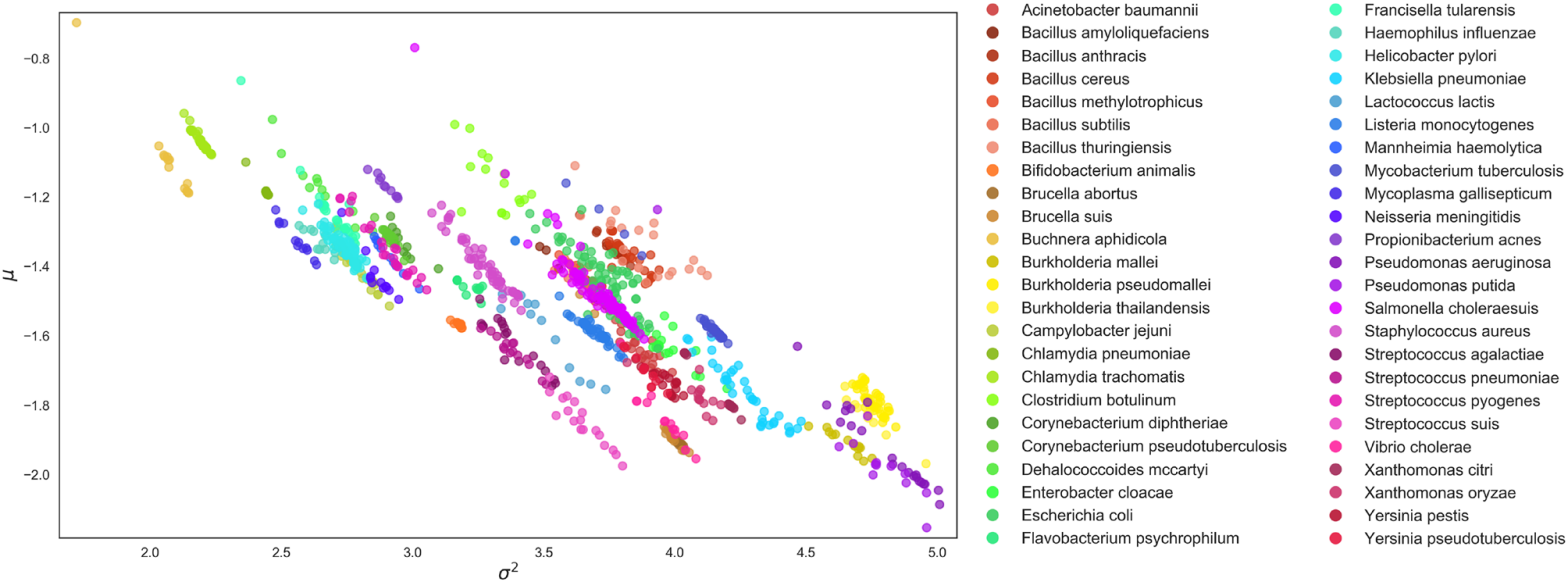
Distribution of species according to the model parameters. Scatter plot of Poisson Log-Normal parameters $\mu$ versus $\sigma ^2$ obtained fitting the protein domains RSAs. Only species represented by at least 10 different strains were included in the plot, for a total of 1173 bacterial genomes which belong to 48 different species. Different colors represent different species as indicated in the legend.

First of all, Fig. 2 shows that $\mu$ has negative values in all bacterial genomes. Recalling that $\mu =r/\gamma$, where *r* is the growth rate and $\gamma$ is the multiplicative constant of the Gompertzian function, which must be positive, this implies that the growth rate of protein domains, *r*, is also negative. Notice that the growth rate can be expressed as the difference between the birth and the death rate, $r=b-d$. Hence, a negative *r* means that the death rate is greater than the birth rate ($d > b$). In the evolutionary model of protein domains, the birth rate *b* has the meaning of duplication rate, while the death rate *d* is the rate at which protein domains are deactivated. A negative *r* hence implies that protein domain deactivation, which is related to the accumulation of events which disrupt the coding sequence of protein domains, happens at a faster rate than the duplication of the whole protein domain sequence within the genome.

Furthermore, the plot of $\mu$ as a function of $\sigma ^2$ (Fig. 2) highlights the negative linear relationship between $\mu$ and $\sigma ^2$. Such relationship can also be deduced mathematically.

Starting from the expressions $\mu =r/\gamma$ and $\sigma ^2=\sigma _e^2 / 2\gamma$, and after simple algebraic manipulation, we can in fact obtain that $\mu = 2r\sigma ^2 / \sigma _e^2$, which explains the negative linear relationship between the two parameters.

Besides the negative relationship, the plot of $\mu$ versus $\sigma ^2$ also highlights the presence of clusters of bacterial genomes with similar ecological features, which are pictured in the plot as roughly parallel stripes (Fig. 2). When we depict bacterial strains belonging to the same species using the same color, it emerges that the stripes are related to the bacterial taxonomy. This result motivates us to introduce a new approach to bacterial phylogeny based on the ecological modeling of protein domains and the consequent estimation of the parameters $\mu$ and $\sigma ^2$.

### Protein domain RSA and evolutionary distance

We propose to calculate the pairwise evolutionary distances between bacteria based on three parameters: the Poisson Log-Normal scale and location parameters discussed above ($\mu$ and $\sigma$), and the density of protein domains in the bacterial genome. Such density describes to which extend the whole bacterial genome is populated with protein domains and it hence constitutes an additional feature of the protein domain ecological dynamics. As detailed in the Materials and Methods, the distance between bacteria is specifically computed as the 3D euclidean distance in the scaled space of $\mu$, $\sigma$, and protein domain density. In the following, we refer to such distance as ‘RSA distance’.

To evaluate the bacterial interrelationships derived from the RSA distances, we compared our results with both the bacterial taxonomic classification and the 16S rRNA gene-based phylogeny. Specifically, starting from the RSA distance matrix we computed a hierarchical clustering of bacteria and we compared the resulting clusters with those obtained from the 16S rRNA gene-based distance matrix. Both clustering results were then compared with the bacterial taxonomic classification.

Notice that the usage of both 16S rRNA phylogeny and bacterial taxonomic classification allows us to exploit the complementary information that these two approaches provide, despite their intrinsic connection. Namely, modern microbial taxonomy is mostly based on 16S rRNA gene^6^ and, on the other hand, the cutoffs commonly used in 16S rRNA phylogeny originated from phenotype-based taxonomy^31^. However, while taxonomy allows us to assign human interpretable names to bacteria, to associate such names with phenotypic properties, and to classify bacteria into a predefined hierarchy, 16S rRNA phylogeny provides a quantitative measurement of the evolutionary distance between bacteria that can be compared with the RSA distance without setting any pre-defined threshold. Moreover, the usage of 16S rRNA phylogeny allows us to investigate the bacterial relationships at the intraspecies level, for which the taxonomic classification is not available.

As detailed in the Materials and Methods, 16S rRNA distances were calculated based on the bacterial 16S rRNA gene reference sequences, following the standard procedure^32^. Taxonomic classification, instead, was retrieved from NCBI and included the following levels: phylum, class, order, family, genus and species. In order to obtain a comparable number of clusters from all three methods, we considered separately each taxonomic level and we cut the 16S rRNA and the RSA -based hierarchical trees so as to get a number of clusters equivalent to the number of taxa available at the selected taxonomic level.

**Figure 3:**
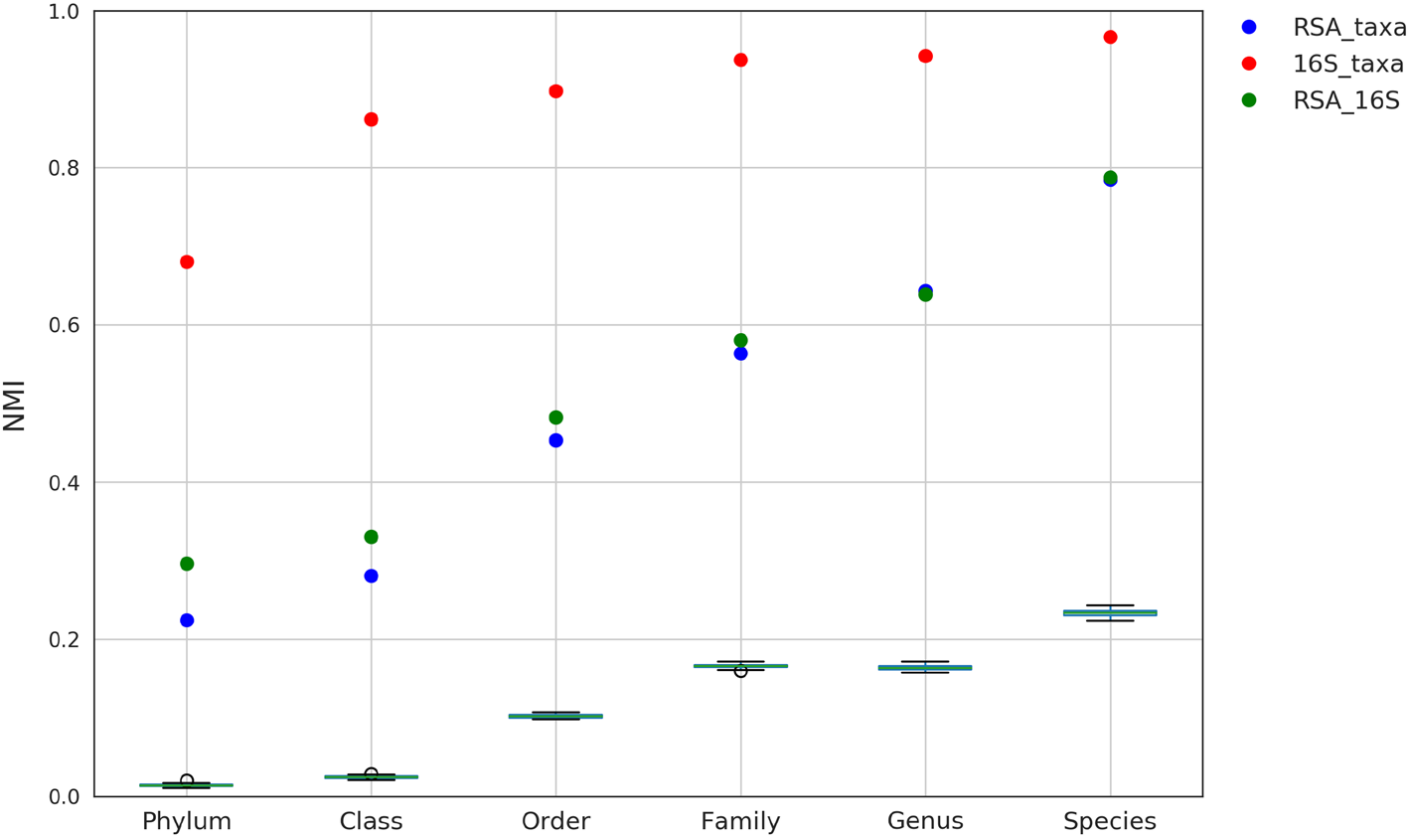
Comparison between the three clustering results at different taxonomic levels. NMI scores (*y-axis*) are calculated as a measurement of agreement between clusters based on: RSA method and taxonomy (*blue*), 16S rRNA gene and taxonomy (*red*), RSA method and 16S rRNA gene (*green*). Different taxonomic levels are considered for the comparison: phylum, class, order, family, genus and species (*x-axis*). The box plots represent the baselines of NMI score and are based on simulations.

At each taxonomic level, the Normalized Mutual Information (NMI) was used as a measurement of agreement between different clustering solutions^33^. Notice that, while the theoretical range of the NMI score is the interval $\left[ 0,1\right]$, NMI is biased towards clustering solutions with more clusters and fewer data points^34^. Consequently, the baseline of NMI score in practise is not zero and relatively high NMI scores can be an artifact caused by the low ratio between number of bacteria and number of taxonomic groups. To make the comparison fair, we used simulations to calculate the baseline NMI for each taxonomic level (box plots of Fig. 3).

As expected by their intrinsic relationship, taxonomy and 16S rRNA phylogeny show high agreement (red dots in Fig. 3). RSA-based clusters, instead, show a certain deviation from both taxonomy (blue dots in Fig. 3) and phylogeny (green dots in Fig. 3). For both comparisons, however, the NMI scores are still evidently higher than the baseline, signifying that the RSA model captures phylogenetic signals to a certain degree. Comparing the obtained NMI scores with the baseline, we notice that the agreement between RSA-based clusters and both taxonomy and phylogeny increases at lower taxonomic levels, reaching the maximum at species level. Taking as ground truth the taxonomic classification, the total purity of the RSA-based clusters at species level is 0.65, signifying that 65% of bacteria are correctly classified.

To assess the robustness and stability of the RSA-based phylogeny, with regard to the choice of protein domains, we randomly selected subsamples of protein domains in different proportions (from $10\%$ to 90% of all protein domains). The reconstructed phylogenetic trees were then compared with the phylogenetic tree obtained using all protein domains (see Materials and Methods for details), and the correlation between the trees was calculated (see Supplementary Fig. S6). As expected, with larger proportions of protein domains taken into account, the correlation between subsample-based phylogeny and base phylogeny increases. For larger subsampling proportions, the compared phylogenetic trees are in good agreement: for a subsample with 90% of protein domains, the mean cophenetic correlation is equal to 0.74, and the mean common-nodes-correlation is equal to 0.68. We notice that the common-nodes-correlation is more stable compared to the cophenetic correlation, as expected since cophenetic correlation is affected by the height of the phylogenetic trees. The results suggest that the overall structure of the phylogenies is stable even for smaller subsampling proportions, while subsampling height of the branches correlates with the full-data height only at larger subsampling proportions.

To evaluate the intraspecies composition obtained from the RSA-based clustering, we selected the subset of species for which at least 10 different strains were present in our data (48 species). Among them, we selected the species where hierarchical clustering showed a clear separation of clusters (including outliers) and for which published literature characterizing at least some of the strains was available (6 out of 48 species). For these 6 species, we again assessed the robustness and stability of RSA phylogenies, as detailed in the "Materials and methods" section. Our results suggest (see Supplementary Fig. S7) that the subsample-based phylogenies are in good agreement with the full-data phylogenies, especially for larger subsampling proportions. We notice the correlations is larger than in the case of phylogenetic trees for randomly selected 100 bacteria (Supplementary Fig. S6), especially for certain species (i.e., *Xanthomonas citri*). This could be attributed to the smaller size of the phylogenetic tree. However, the species with similar phylogenetic tree size still show differences in correlation (i.e., *Xanthomonas citri* and *Francisella tularensis*), suggesting that the RSA-based distance matrix between the strains of *Xanthomonas citri* carries stronger phylogenetic signal. Comparing 6 observed species with the randomly sampled subsets of 100 bacteria, we can analogously conclude that the RSA-captured phylogenetic signal is stronger within the species. In the following, we discuss the results obtained for the 6 selected bacterial species in more details.

**Figure 4:**
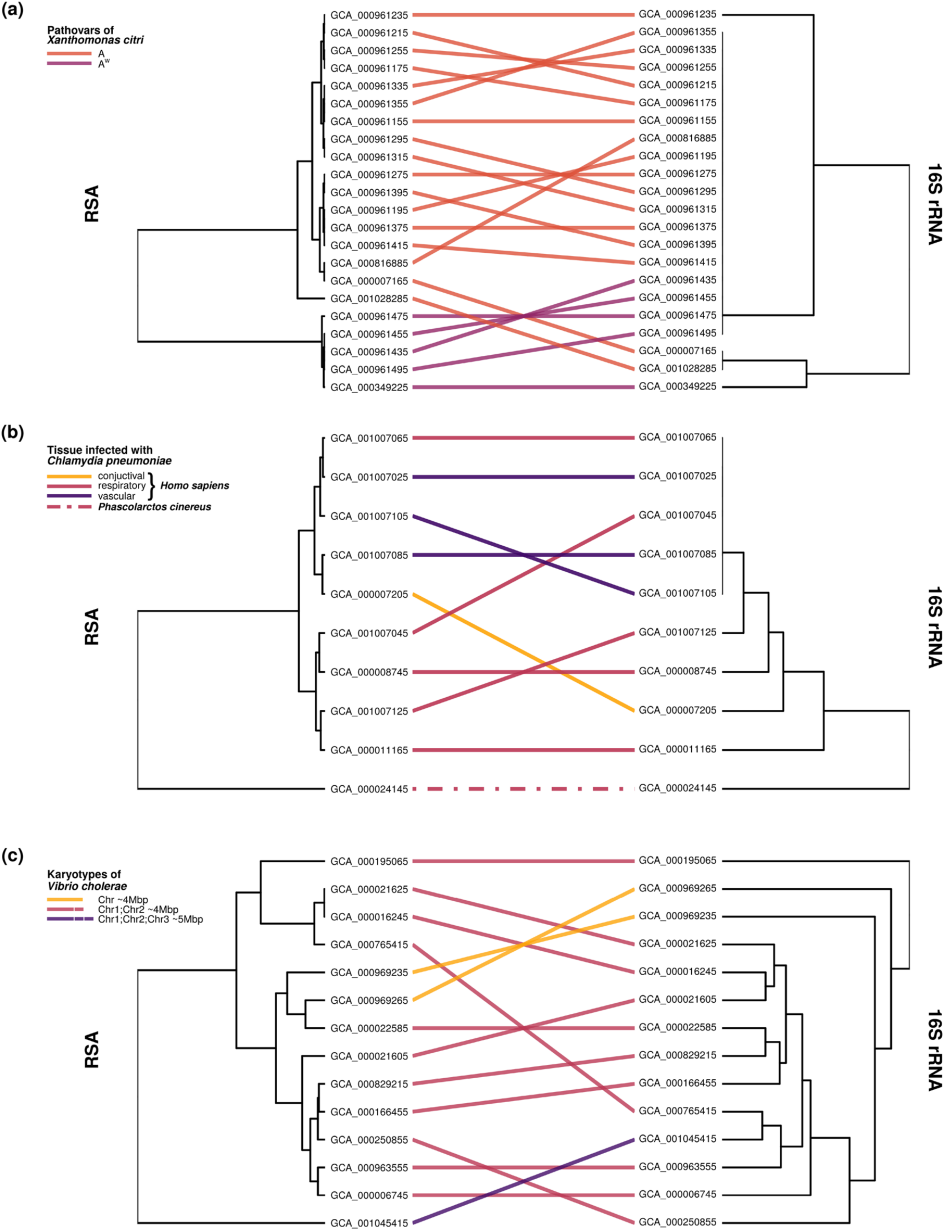
(Previous page.) Hierarchical clustering of bacteria at the intraspecies level, comparing solutions obtained by RSA and 16S rRNA method. Each subplot shows a tanglegram with RSA-based dendrogram on the left and 16S rRNA-based dendrogram on the right. Lines connect the same bacteria from two dendrograms. The color/type of the line represents the feature of the bacterium it connects. (**a**) 22 strains of *Xanthomonas citri* belong to two different pathovars: A (*orange*) and $\hbox {A}^{\mathrm{W}}$ (*purple*). (**b**) 10 strains of *Chlamydia pneumoniae* are isolated from different tissues: conjuctival (*yellow*), respiratory (*magenta*) and vascular (*violet*). 9 strains represented with solid line are human (*Homo sapiens*) pathogens while the one strain represented by dashed line is koala (*Phascolarctos cinereus*) pathogen. (**c**) 14 strains of *Vibrio cholerae* are colored based on their karyotype. 11 strains have two circular chromosomes Chr1 ($\sim 3$ Mb) and Chr2 ($\sim 1$ Mb) (*magenta*). 2 strains have one $\sim$4 Mb long circular chromosome (*yellow*). One strain has three chromosomes Chr1 ($\sim$3 Mb), Chr2 ($\sim$1 Mb) and Chr3 ($\sim$1 Mb) (*violet*).

### RSA-based method distinguishes subspecies infecting different hosts

*Xanthomonas citri* subsp. *citri* (XCC) and *Chlamydia pneumoniae* (*Cpn*) are two species whose subspecies can infect different hosts. Here we show that the RSA-based method correctly discriminates such subspecies even when their divergence is not detected comparing the 16S rRNA gene sequences.

*Xanthomonas citri* subsp. *citri* (XCC) is a causal agent of citrus canker type A, a bacterial disease affecting different plants from the genus *Citrus*. While citrus canker A infects most citrus species, two of its variants, A* and $\hbox {A}^{\mathrm{W}}$, have a much more limited host range with XCC pathotype $\hbox {A}^{\mathrm{W}}$ infecting only Key lime (*C. aurantifolia*) and alemow (*C. macrophylla*)^2^. Our data set includes 17 strains of XCC pathotype A and 5 strains of XCC pathotype $\hbox {A}^{\mathrm{W}}$^2^. RSA-based clustering of the 22 XCC strains identifies two separated clusters (Fig. 4a, left) which coincide with the two XCC pathotypes. Concurrently, clustering based on 16S rRNA gene fails to identify the two pathotypes of XCC (Fig. 4a, right). This suggests that even though pathotypes A and $\hbox {A}^{\mathrm{W}}$ have different hosts, their diversification is not reflected in the variability of the 16S rRNA gene. On the other hand, modeling the protein domain RSA of the two pathotypes succesfully captures the different functions of their proteomes.

Another important aspect of the citrus canker is the geographical spread of the disease. The 22 strains of XCC included in our data set have diverse geographical origin. While all $\hbox {A}^{\mathrm{W}}$ strains were sampled from USA, strains of pathotype A originate from USA, Brazil and China. RSA clustering of 17 A-type strains colored by their sampling location shows a geographical pattern (Supplementary Fig. S2) similar to the one obtained by Patané et al.^2^ using a maximum likelihood tree based on 1785 concatenated unicopy genes, with the only exception of strain jx-6 ($\text {GCA}\_001028285$) coming from China.

For what concerns *Chlamydia pneumoniae* (*Cpn*), this is an obligate intercellular parasite which is widespread in human population and causes acute respiratory disease. Besides humans, different animal species can be infected with *Chlamydia pneumoniae*. Our data set includes 9 strains which infect humans (*Homo sapiens*) and 1 strain isolated from koala (*Phascolarctos cinereus*). RSA-based clustering clearly separates such isolate from the group of highly similar human isolates (Fig. 4b, left). This result is confirmed by 16S rRNA-based clustering (Fig. 4b, right) and is in agreeement with previous results in which the comparison of four human-derived isolates and the koala strain LPCoLN ($\text {GCA}\_000024145$) through whole-genome sequencing showed a much higher variation between human and koala-derived strains than within the human-derived strains^35^.

Another peculiarity of *Chlamydia pneumoniae* is tissue tropism. The human-derived strains of *Chlamydia pneumoniae* can in fact be divided into conjuctival, raspiratory and vascular based on their tissue of origin. *Cpn* tissue tropism was the focus of the study conducted by Weinmaier et al., where whole-genome sequences of multiple *Cpn* strains isolated from different human anatomical sites were compared and animal isolates were used as outgroup^3^. Weinmaier et al. found a good agreement between the anatomical origin of strains and the maximum likelihood phylogenetic tree based on all SNPs. However, they could not obtain a clear separation between anatomical subgroups of *Cpn*. Our results show that the RSA-based method partially succeeds in separating subspecies related to different tissues (Fig. 4b, left). The RSA-based dendrogram, in fact, shows a cluster of four respiratory bacteria. However, it does not separate the other subspecies by infection site, suggesting that tissue tropism is not entirely captured by our method.

### RSA-based method discriminates subspecies with different genome composition

In some cases, subspecies of the same species are characterized by global differences in the genome composition. This is, for example, the case of *Vibrio cholerae* and *Buchnera aphidicola*. Here, we show that the RSA-based model is able to capture such differences and to discriminate subspecies with known different genomic peculiarities.

*Vibrio cholerae* is the causative agent of cholera disease. Its genome is normally composed of two chromosomes: Chr1 ($\sim 3$ Mb) and Chr2 ($\sim 1$ Mb). However, some strains show a different karyotype. The two strains $1154\text {-}74$ ($\text {GCA}\_000969235$) and $10432\text {-}62$ ($\text {GCA}\_000969265$), for instance, underwent the process of chromosomal fusion and possess only one $\sim 4$ Mb long circular chromosome, which shows a high degree of synteny with the two chromosomes of the more common strains^36^. The strain $\text {TSY}216$ ($\text {GCA}001045415$), on the other hand, besides having the original two chromosomes, also contains an additional $\sim 1$ Mb long replicon, which does not share any conserved region with Chr1 and Chr2^37^. For these reasons, we expect the single- and two-chromosome strains to be phylogenetically closer to each other than to the three-chromosome strain, which contains the extra replicon. The 16S rRNA gene-based clustering, however, does not identify any clear separation between the three types of strains (Fig. 4c, right). As a matter of fact, all the 16S rRNA gene copies of all the *Vibrio cholerae* strains included in our data set are located on $\sim 3$ Mb long chromosome, which shows high synteny across all strains. It is therefore not surprising that the comparison of the 16S rRNA genes does not capture the global genomic differences that exist between the considered strains. On the other hand, the results obtained with the RSA-based clustering show a clear distinction of the strains with different genomic structure (Fig. 4c, left). The reason for the success of the RSA-based method lies in the theoretical definition of RSA-based distance. In fact, the RSA-based distance depends on the Poisson Log-Normal location parameter $\sigma ^2$, which increases with the genome length (Supplementary Fig. S1): by definition, $\sigma ^2 = \sigma _e^2 / 2\gamma$, and, while the environmental noise $\sigma _e^2$ can be reasonably considered independent of the genome length, the density regulation $\gamma$ is expected to be stronger for smaller genomes, which repesent a scarcer environment with less resources.

*Buchnera aphidicola* is a bacterial species in mutualistic endosymbiotic relationship with different aphids (members of superfamily *Aphidoidea*). As many endosymbionts, *Buchnera aphidicola* underwent the process of genome reduction as an adaptation to the host-associated lifestyle and has a genome with length $<1$ Mb. One of the main processes which contributed to genome reduction is gene inactivation followed by progressive gene disintegration^38^. An intermediate step of this process is pseudogenization. For this reason, we expect the number of pseudogenes within a genome to be related to its evolutionary state and strains with similar numbers of pseudogenes to be phylogenetically similar. Among the 13 strains of *Buchnera aphidicola* available in our data set, we observe a high variability in the number of pseudogenes, with 12 strains having between 7 and 63, and one strain (JF98, $\text {GCA}\_000183305$) having 176 pseudogenes. Since the total number of genes (considering both protein coding genes and pseudogenes) is similar in all strains, this implies that JF98 has a smaller proteome compared to the others. Our results show that the divergence of strain JF98 from the others is evident from the RSA-based clustering (Supplementary Fig. S3-a, left), while it is not clear from the 16S rRNA-based one (Supplementary Fig. S3-a, right).

On the other hand, another outstanding property of the *Buchnera aphidicola* strains is their capability to infect different hosts. Focusing on this aspect, we notice that the RSA-based clustering only partially agrees with a grouping of the strains based on the infected host. The 16S rRNA-based method, instead, finds more coherent results (Supplementary Fig. S3-b), possibly due to the fact that endosymbionts and their hosts tend to co-evolve together once their endosymbiotic relationship starts^4^.

### Distinct bacterial isolates detected by the RSA-based method

For several species in our data set, the RSA-based phylogenic tree indicates the presence of outlier strains (see Supplementary Fig. S8). For two of such species (*Listeria monocytogenes* and *Francisella tularensis*), in particular, we were able to find bibliographic information at the strain level which provides a biological explanation to our findings.

*Listeria monocytogenes* is a food-borne pathogenic bacterium which causes listeriosis in humans. From the 48 *Listeria monocytogenes* strains present in our data set, RSA-based clustering identifies a subgroup of two strains (Supplementary Fig. S4). These two strains, La111 ($\text {GCA}\_000382925$) and N53-1 ($\text {GCA}\_000382945$), seem to have very similar proteome composition which differs from that of the other strains. Holch et al. investigated strains La111 and N53-1 in their study on bacterial persistence in *Listeria monocytogenes*^39^. Based on whole-genome analysis, the authors found that these two strains, which were isolated 6 years apart from different Danish fish processors, are extremely similar and collectively different from the other analyzed strains, in agreement with our RSA-based results.

*Francisella tularensis*, instead, is an intracellular bacterium which is a causal agent of tularemia. Our data set includes 25 *Francisella tularensis* strains from different subspecies: *tularensis*, *holarctica*, *mediasiatica* and *novicida*. While neither RSA- nor 16S rRNA-based clustering accurately separate subspecies of *Francisella tularensis*, the RSA method identifies one outlier, strain TIGB03 ($\text {GCA}\_000248415$) (Supplementary Fig. S5). Unlike the other 24 virulent strains, strain TIGB03 is an attenuated *tularensis* strain. This strain was described by Modise et al. as an attenuated O-antigen mutant of the virulent strain TI0902 ($\text {GCA}\_000248435$)^5^. Indeed, we notice that 16S rRNA genes of these two strains are identical (Supplementary Fig. S5) and thus 16S rRNA-based clustering is unable to detect the mutant strain. Comparing whole-genome sequences of strains TIGB03 and TI0902, Modise et al. found 31 nonsynonymous point mutations and a 75.9 kb long duplicated region in the mutant strain TIGB03. However, such long duplication does not make TIGB03 an outlier in terms of genome length (the genome length of TIGB03 is 1.97 Mb and that of the other 24 strains included in our study ranges from 1.86 to 2.05 Mb). This indicates that the presence of nonsynomymous mutations led to different proteome composition, which is recognized by our method.

## Conclusion

The *de novo* clustering of phylogenetically similar metagenomes is a crucial step for the study of bacterial populations. To this aim, a typical approach is the pairwise comparison of 16S rRNA sequences derived from the biological sample of interest. After computing the pairwise distance matrix, clusters are defined based on a custom similarity threshold. Then, clusters are usually compared to databases of annotated sequences to derive the taxonomic classification, that is to associate human-made hierarchical labels (bacterial names) to the detected metagenomes. While taxonomic classification allows to recognize known bacteria and their phenotype, *de novo* clustering potentially allows to obtain a higher resolution of the bacterial population at the intraspecies level, unaffected by the possible bias of human-made classifications.

Here, we proposed to measure the phylogenetic distance between bacteria, based on their genomic evolving units: protein domains. Specifically, we exploited the ecological modeling of ecosystems proposed by Engen and Lande^23^ to describe the evolution of protein domains, considering them as ‘species’ that populate the genome. Based on such modeling, we derived a new phylogenetic distance that can be computed fitting the protein domains Relative Species Abundance distribution (RSA). We showed that clustering the bacterial genomes based on 16S rRNA or exploiting our model allows to capture different features in terms of both genome composition (e.g. presence of additional replicon, pseudogenes, duplicated regions, or nonsynomymous mutations) and bacterial phenotype (e.g. infected host). Our results hence open to the possibility of developing an algorithm in which both pieces of information are exploited to obtain an overall best inference of bacterial phylogeny. This will hopefully be part of future works.

The proposed approach is especially promising when samples are analysed by means of shotgun sequencing, rather than 16S rRNA sequencing^40^. Indeed, 16S rRNA sequencing is a powerful method to detect and compare metagenomes. However, it is limited by being based on the measurement of a single gene. On the other hand, information on the whole metagenome can be derived when using shotgun sequencing^40^. In terms of phylogeny, 16S rRNA sequencing detects only metagenomic divergences which directly affect the 16S rRNA gene, and its ability to discriminate metagenomes at the intraspecies level is therefore limited^6^. Our RSA-based method, on the other hand, provides a global measurement based on the whole proteome. As highlighted by our results, these two approaches are hence somehow complementary, being the RSA-based method more sensitive to evolutionary processes that affect the metagenome globally, possibly leaving the 16S rRNA gene unaffected, and the 16S rRNA-based method being instead more sensitive when such gene is involved, such as in the case of *Buchnera aphidicola*. Overall, our results show that the RSA-based approach is a promising method that could be exploited to study the metagenomic composition of samples, in particular when shotgun sequencing is performed and the whole metagenome could be reconstructed, though experimental validation would be required to definitely assess the efficacy of our approach.

## Materials and methods

### Data retrieval

The genome sequences of 3370 bacteria were downloaded from the NCBI database^41^. Draft genome sequences were discarded and only the higher quality fully circular genome sequences were retained. GeneBank files containing genome sequences and existing annotations were retrieved from the NCBI database and imported into the Semantic Annotation Platform for Prokaryotes^42^ using the EMBL/GBK to RDF SAPP module. *De novo* identification of genetic elements (gene calling) was performed using Prodigal (2.6)^43^ with codon table 11, which is recommanded when dealing with bacterial genomes. Dedicated SPARQL queries were built to extract all proteins and their sequences from the RDF triplestore used by SAPP to store the intermediate results. InterProScan^44^ was used to identify protein domains in the corresponding sequences. Due to the high number of distinct protein sequences to be analyzed, the SURFsara GRID infrastructure was used^45^ to concurrently analyze the sequences. Dedicated SPARQL queries were used to retrieve all the identified domains and assign them to the originating protein and bacterial genome. Finally, the matrix generating module from SAPP was used to generate a matrix containing the number of instances of the detected domain (domain abundance) for each of the studied genomes and for each of the identified protein domains. Overall 3370 bacterial genomes were analyzed and 13,934 distinct domains were identified.

### Model fitting and selection

Protein domains RSAs were fitted with the Maximum Likelihood Estimation method implemented in R “sads” package v.0.4.2^46^. Data were modelled with a truncated Poisson Log-Normal, a truncated Log-Series and a truncated Negative Binomial distribution, so that to test and compare different ecological hypothesis. In all cases, truncation was performed to exclude the zero abundance class, that is not observable in empirical data. Two bacterial genomes which couldn’t be fitted due to extreme RSA distributions are $\text {GCA}\_000200735$ and $\text {GCA}\_000831405$. After removal of these bacteria, we proceeded with the analysis of the remaining 3368 bacterial genomes. Akaike Information Criterion (AIC)^30^ and R-squared were computed to assess the models performances and for model selection.

### Calculation of RSA model-based distance matrix

Fitting truncated Poisson Log-Normal distribution to protein domain RSA, we obtained estimates $\mu$ and $\sigma$ for 3368 bacteria. In addition to Poisson Log-Normal parameters, we calculated protein domain density for every bacteria as ratio between total number of protein domains present in the genome and the genome length. RSA distance between each pair of bacteria was calculated as 3D euclidean distance in the scaled space of $\mu$, $\sigma$ and protein domain density. Scaling was performed independently at each dimension subtracting the mean and dividing by standard deviation.

### Calculation of 16S rRNA gene-based distance matrix

In order to calculate phylogenetic distances based on 16S rRNA gene, we used the silva database^47^ to retrieve the 16S rRNA reference sequences of the bacterial species for which protein domain data were available. For 48 bacteria, the 16S rRNA sequence was not present, so we considered only the remaining 3320 bacteria for the following analysis. Since the same bacterial genome can have multiple different copies of 16S rRNA gene, as 16S rRNA distance between a pair of bacteria we used the mean pairwise distance between all pairs of 16S rRNA sequences within two genomes^48^. The alignment of 16S rRNA sequences and calculation of distances was performed with mothur^32^ “pairwise.seqs” function with default parameters.

### Comparison of different clustering solutions

For each taxonomic level, we considered only bacteria for which the classification was known and which belonged to a taxonomic group with at least 10 members. As a result, we analysed 3270, 3148, 3032, 2714, 2139 and 161 bacteria at phylum, class, order, family, genus and species level, respectively. They belonged to 14, 20, 48, 63, 54 and 48 different taxonomic groups. Hierarchical clustering was performed on both RSA and 16S rRNA distance matrices. For RSA clustering we used Ward’s minimum variance method while for 16S rRNA we used average linkage method. The Ward’s method was used to minimize the total in-cluster variance^49^. Since this method is based on Euclidean distance, it couldn’t be applied to 16S rRNA distance matrix. To get clustering solutions for RSA and 16S rRNA, we cut the hierarchical tree fixing the number of clusters to the number of taxa at the selected taxonomic level. Finally, we used the NMI score as a measure of clustering agreement between the three approaches: RSA, 16S and predefined taxonomy. To calculate the baseline for the NMI score, we compared the taxonomy with clustering solutions based on simulations. For the fixed number of clusters and data points, each simulation assigned random cluster to each data point. Purity of a RSA cluster with respect to taxonomy is calculated as the ratio between the size of the cluster’s most abundant taxonomic group and the cluster size. The total purity of the RSA clustering solution is a weighted average of its clusters’ purities where weight is a cluster size.

### Assessment of robustness and stability of the phylogeny

To assess the robustness and stability of RSA-based phylogeny, regarding the choice of protein domains, we randomly selected subsamples of protein domains and we compared the obtained subsample-based results with the one obtained when all protein domains were used. Specifically, we subsampled protein domains in various proportions (ranging from 0.1 to 0.9), then we fitted the subsampled protein domain RSA distribution with Poisson LogNormal distribution and we used the estimated parameters to calculate the phylogeny of the examined bacteria, following the same methodology used when considering all protein domains. The obtained subsample-based phylogeny (i.e., dendrogram) was then compared to the full-data-based phylogeny calculating the correlation between dendrograms. Two different correlation measurements were used, cophenetic correlation and correlation based on common nodes (i.e., nodes that have the same list of leaves). For the calculations, the function “cor.dendlist” from the R “dendextend” package v.1.14.0^50^ was used. For every subsampling proportion (0.1–0.9), 100 bacteria were randomly chosen from 3368 available bacteria and a random subsample of protein domains was taken from the remaining protein domains (which were present in at least one of the chosen bacteria). The correlation between two 100-leaf dendrograms (computed with all protein domains and with a chosen subsample of protein domains) was calculated. The procedure was repeated for 50 times, resulting in 50 correlation measurements for every subsampling proportion. Additionally, robustness and stability of phylogeny was assessed for 6 bacterial species (*Xanthomonas citri*, *Vibrio cholerae*, *Francisella tularensis*, *Buchnera aphidicola*, *Listeria monocytogenes*, *Chlamydia pneumoniae*). For every bacterial species and for different subsampling proportions (from 0.1 to 0.9), with 100 repeats, a subsample of protein domains was randomly selected and subsample-based phylogeny of the species was recontructed. Correlations between the full-data dendrogram and the 100 subsample-based dendrograms were calculated. Subsequently, the mean and standard deviation of correlation was obtained for every species and every subsampling proportion.

## Supplementary Information

Supplementary Information 1.

Supplementary Information 2.

## Data Availability

The datasets analysed during the current study are available in the NCBI repository^41^ (https://www.ncbi.nlm.nih.gov/). The complete list of accession numbers (GenBank IDs) is provided in the Supplementary material.
