# Supplementary material for: Intraspecies characterization of bacteria via evolutionary modeling of protein domains
Source: Sci Rep. 2022 Oct 5;12:16595. doi: 10.1038/s41598-022-21036-3 (PMC9534902; doi:10.1038/s41598-022-21036-3)
Supplement: Supplementary file 1 — Supplementary Information 1. [file 41598_2022_21036_MOESM1_ESM.pdf]

Supplementary Material

Stochastic process

A stochastic (or random) process is a family of random variables $\{X(t); t \in T\}$ indexed by a parameter t. To properly define a stochastic process, we need to specify the state space S, the index parameter T and the dependence relations among the random variables $X(t)$.
The state space S is the space in which all possible values of random variables $X(t)$ lie. If $S = \mathbb{N}_0 = \{0, 1, 2, \ldots\}$, then we call $X(t)$ a discrete state process. If $S = \mathbb{R}$, then $X(t)$ is a real-valued stochastic process. As with the codomain of any function, S is not uniquely defined but usually the appropriate choice for S is obvious.
The index $t, t \in T$ is usually interpreted as time parameter of the process and T is the set of all times in which we observe the stochastic process. If $T = \{0, 1, 2, \ldots\}$, then $X(t)$ is a discrete time stochastic process. In this case, we often write X_n instead of $X(t)$. If $T = [0, \infty\rangle$, then $X(t)$ is called a continuous time process.

Markov process

Markov process is a stochastic process $\{X(t); t \geq 0\}$ with the following property

$$P(a < X(t) \leq b \mid X(t_1) = x_1, X(t_2) = x_2, \ldots, X(t_n) = x_n) =$$
$$P(a < X(t) \leq b \mid X(t_n) = x_n), \quad \text{whenever } t_1 < t_2 < \cdots < t_n < t. \tag{1}$$

Poisson process

Homogenouos Poisson process A homogenous Poisson process with rate λ is a stochastic process $\{X(t); t \geq 0\}$ on the nonnegative integers which has the following properties:

- $X(0) = 0$.
- $\{X(t)\}$ has independent increments, that is, for every pair of disjoint time intervals $[t_1, t_2]$, $[t_1, t_2]$, with $t_1 \leq t_2 < t_3 \leq t_4$, the increments $X(t_4) - X(t_3)$ and $X(t_2) - X(t_1)$ are independent random variables.
- $P(X(t+h) - X(t) = 0) = 1 - \lambda h + o(h)$ as $h \downarrow 0 \quad (x = 0, 1, 2, \ldots)$.
- $P(X(t+h) - X(t) = 1) = \lambda h + o(h)$ as $h \downarrow 0$.
- $P(X(t+h) - X(t) \geq 2) = o(h)$ as $h \downarrow 0$.

Notice that we say that an arbitrary function $f \in o(h)$ if $\lim_{h \downarrow 0} \frac{f(h)}{h} \to 0$.

Inhomogeneous Poisson process If instead of a constant, $\lambda(t) : [0, \infty\rangle \to [0, \infty\rangle$ is an integrable function, then we get an inhomogeneous (or nonhomogeneous) Poisson process. All of the properties (a)-(e) still hold replacing λ with $\lambda(t)$ as does the following relation

$$X(t+h) - X(t) \sim Poisson\left(\int_t^{t+h} \lambda(\alpha) d\alpha\right). \tag{2}$$

Brownian motion

Brownian motion is a stochastic process $\{X(t); t \geq 0\}$ with the following properties:

- Every increment $X(t+h) - X(t)$ is normally distributed with mean 0 and variance $\sigma^2 h$ with σ^2 being a constant.
- $\{X(t)\}$ has independent increments, that is, for every pair of disjoint time intervals $[t_1, t_2]$, $[t_1, t_2]$, with $t_1 \leq t_2 < t_3 \leq t_4$, the increments $X(t_4) - X(t_3)$ and $X(t_2) - X(t_1)$ are independent random variables.
- $X(0) = 0$ and $X(t)$ is almost surely continuous functions of t.

If $\sigma^2 = 1$, then a Brownian motion is called a *standard Brownian motion*.

Diffusion process

Diffusion process is a Markov process $\{X(t);t\geq 0\}$ whose state space is an interval I with endpoints $-\infty\leq l<r\leq\infty$ with the following properties:

- $\lim_{h\downarrow 0}\frac{1}{h}P\left(|X(t+h)-x|>\varepsilon \mid X(t)=x\right)=0$ for every $\varepsilon>0$ and for all $x\in I$.
- $\lim_{h\downarrow 0}\frac{1}{h}E\left[X(t+h)-X(t)\mid X(t)=x\right]=\mu(x,t)$, where $\mu(x,t)$ is a continuous function of x and t.
- $\lim_{h\downarrow 0}\frac{1}{h}E\left[(X(t+h)-X(t))^2\mid X(t)=x\right]=\sigma^2(x,t)$, where $\sigma^2(x,t)\geq 0$ is a continuous function of x and t.

The function $\mu(x,t)$ is called the *infinitesimal mean* and the function $\sigma^2(x,t)$ is called *infinitesimal variance*. Brownian motion is a regular diffusion process on the interval $\langle-\infty,\infty\rangle$ with $\mu(x)=0$ and $\sigma^2(x)=\sigma^2$, where σ^2 is a constant.
Additionally, a diffusion process is *regular* if starting from any point in the interior of I any other point in the interior of I may be reached with positive probability.

Transformation formula for a diffusion process Let $\{X(t);t\geq 0\}$ be a regular diffusion process whose state space is an interval I having endpoints l and r, and suppose $\{X(t)\}$ has infinitesimal parameters $\mu(x)$ and $\sigma^2(x)$. Let g be a strictly monotone function on I with continuous second derivative $g''(x)$ for $l<x<r$. Then $Y(t)=g(X(t))$ defines a regular diffusion process on the interval with endpoints $g(l)$ and $g(r)$, and $\{Y(t)\}$ has infinitesimal parameters

$$\mu_Y(y)=\frac{1}{2}\sigma^2(x)g''(x)+\mu(x)g'(x) \tag{3}$$

$$\sigma_Y^2(y)=\sigma^2(x)\left[g'(x)\right]^2 \tag{4}$$

where $y=g(x)$.

Stochastic differential equation (SDE)

Let $\{B(t);t\geq 0\}$ be a standard Brownian motion. The "process" $dB(t)/dt=W(t)$ is called a Gaussian white noise "process". A classic stochastic differential equation has the following form

$$dX(t)=f(X(t),t)dt+g(X(t),t)dB(t). \tag{5}$$

The Itô's solution to the SDE (5) is a diffusion process with infinitesimal mean

$$\mu(x,t)=f(x,t) \tag{6}$$

and infinitesimal variance

$$\sigma^2(x,t)=g^2(x,t). \tag{7}$$

Solution to the Engen and Lande's SDE

Engen and Lande assumed the process for each species, in the context of population dynamics, is a diffusion process which is a solution of a stochastic growth equation

$$\frac{dx}{dt}=rx-xg(x)+x\sigma_r(x)\frac{dB(t)}{dt}, \tag{8}$$

where $\sigma_r^2(x)=\sigma_e^2+\sigma_d^2/x$.
To solve the SDE (8), we first write it in a the following concise form

$$\frac{dx}{dt}=r'x-xg(x), \tag{9}$$

where $r'=r+\sigma_r dB(t)/dt$ and $\sigma_r^2=\sigma_e^2+\sigma_d^2/x$.
Now we introduce the substitution $y=\ln x$. As a result of the substitution, we have $dy=dx/x$ and $x=e^y$ which together with (9) gives a SDE for y

$$\frac{dy}{dt}=[r-g(e^y)]+\sigma_r(e^y)\frac{dB(t)}{dt}. \tag{10}$$

Applying Itô approach, the process y is a diffusion process with infinitesimal mean $r - g(e^y)$ and infinitesimal variance $\sigma_r^2(e^y)$. Using the transformation formula for diffusion processes for $x = e^y$, we get that x is a diffusion process with infinitesimal mean $m(x)$ and variance $v(x)$ given by

$$m(x) = \left[r + \frac{1}{2}\frac{\sigma_d^2}{x} + \frac{1}{2}\sigma_e^2\right] x - xg(x) \tag{11}$$

$$v(x) = \sigma_d^2 x + \sigma_e^2 x^2. \tag{12}$$

Poisson Log-Normal RSA

The protein domain RSA has been fitted with a Poisson Log-Normal distribution, which is a Poisson distribution with rate $\lambda(x)$ distributed according to a Log-Normal:

$$\lambda(x) = \text{Log-Normal}(x) = \frac{1}{x\sigma\sqrt{2\pi}} e^{-\frac{(ln(x)-\mu)^2}{2\sigma^2}}, \tag{13}$$

According to Engen and Lande's model, the parameters of the Log-Normal distribution are $\mu = r/\gamma$ and $\sigma^2 = \sigma_e^2/2\gamma$, where: i) r is the constant growth rate of individuals within a protein domain family (see equation (2) of the main text) and can be expressed as the difference between the birth and the death rate $r = b - d$; ii) γ is the Gompertzian constant that appears in the Gompertzian density regulation function $g(x) = \gamma \ln(x + \varepsilon)$; iii) σ_e^2 is the environmental stochasticity.

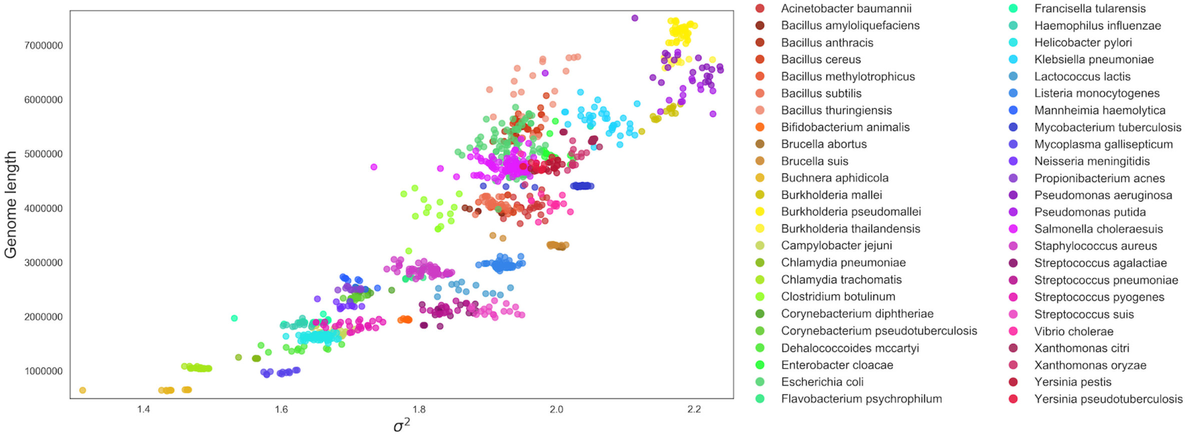

Figure S1. Scatter plot of bacterial genome length versus Poisson Log-Normal parameter σ^2 obtained fitting the protein domains RSAs. Figure shows only those species which are represented with at least 10 different strains in our dataset. There are in total 1173 bacteria which belong to 48 different species. Different colors represent different species, as indicated in the legend.

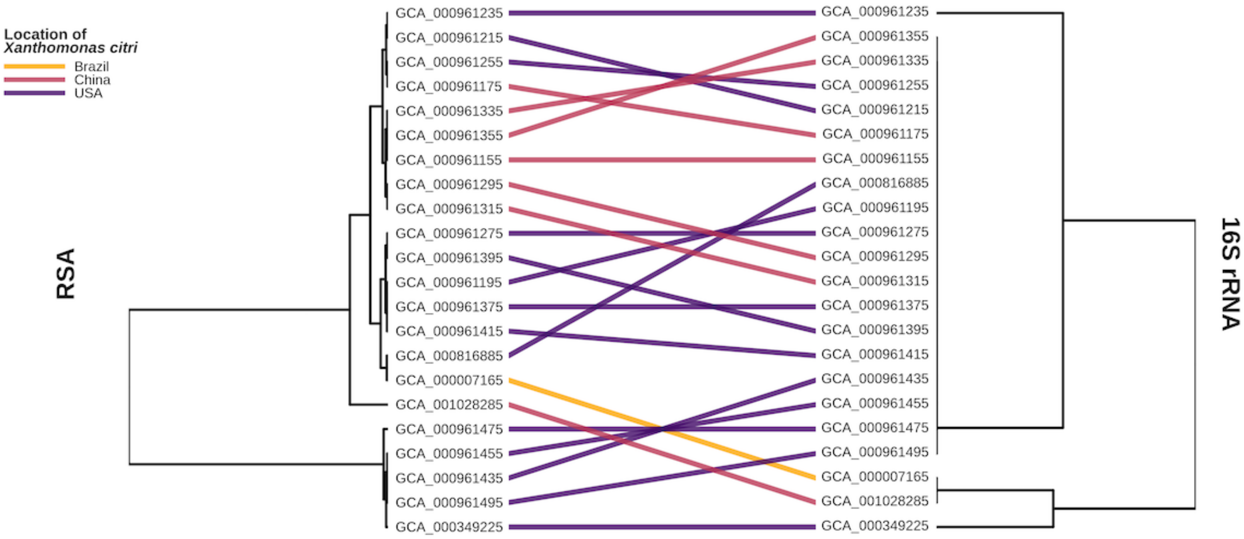

Figure S2. Hierarchical clustering of 22 strains of *Xanthomonas citri*, by RSA method (*left*) and based on 16S rRNA gene (*right*). The strains have different origin: Brazil (*yellow*), China (*magenta*) and USA (*violet*).

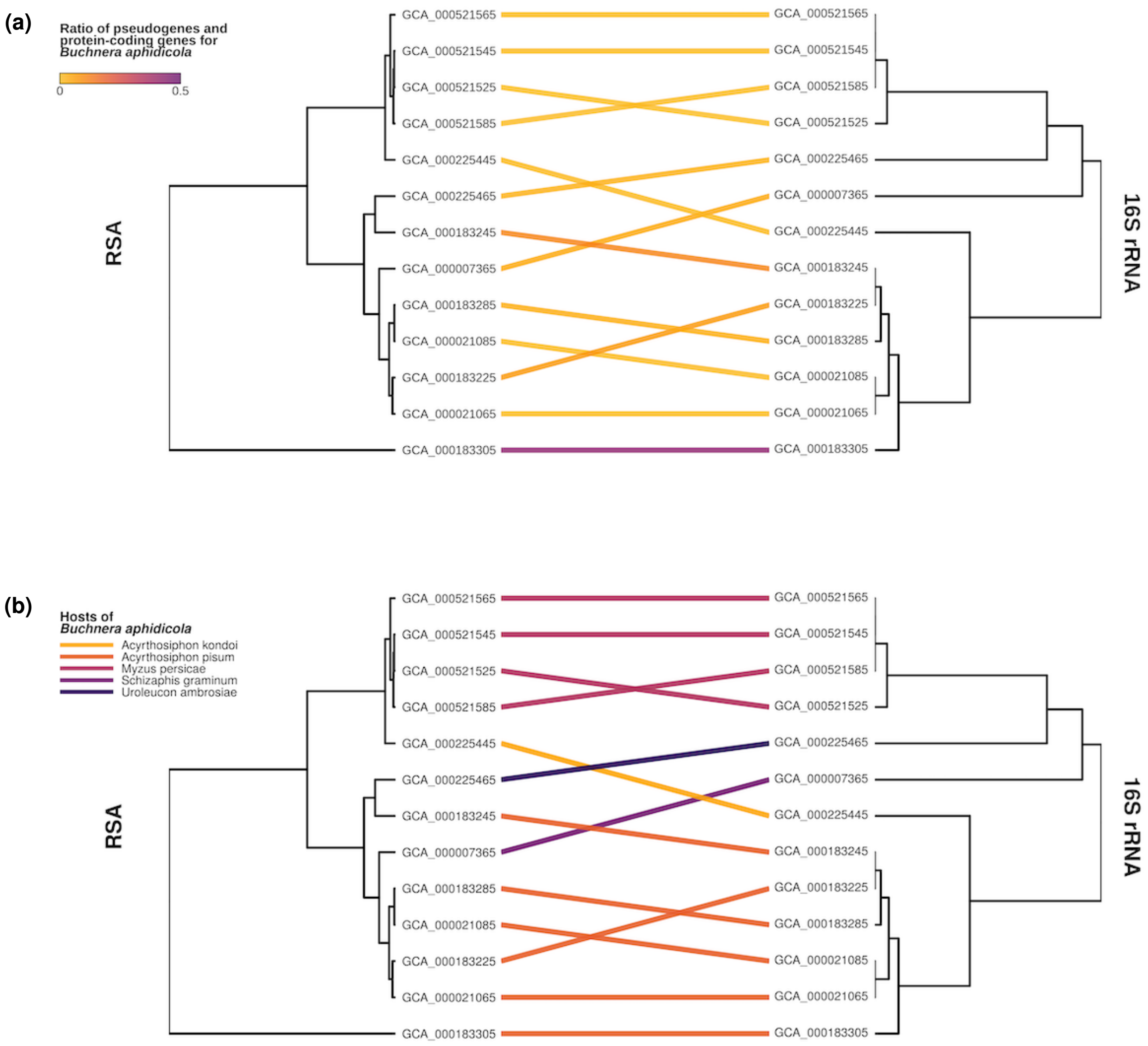

Figure S3. Hierarchical clustering of 13 strains of *Buchnera aphidicola*, by RSA method (*left*) and based on 16S rRNA gene (*right*). **(a)** Strains are colored by the ratio between number of pseudogenes and protein-coding genes. We notice that 12 stains have relatively low ratio while strain GCA_000183305 has ratio equal to 0.429. **(b)** 13 strains have different aphid hosts: *Acyrthosiphon kondoi* (*yellow*), *Acyrthosiphon pisum* (*orange*), *Myzus persicae* (*magenta*), *Schizaphis graminum* (*violet*) and *Uroleucon ambrosiae* (*navy*).

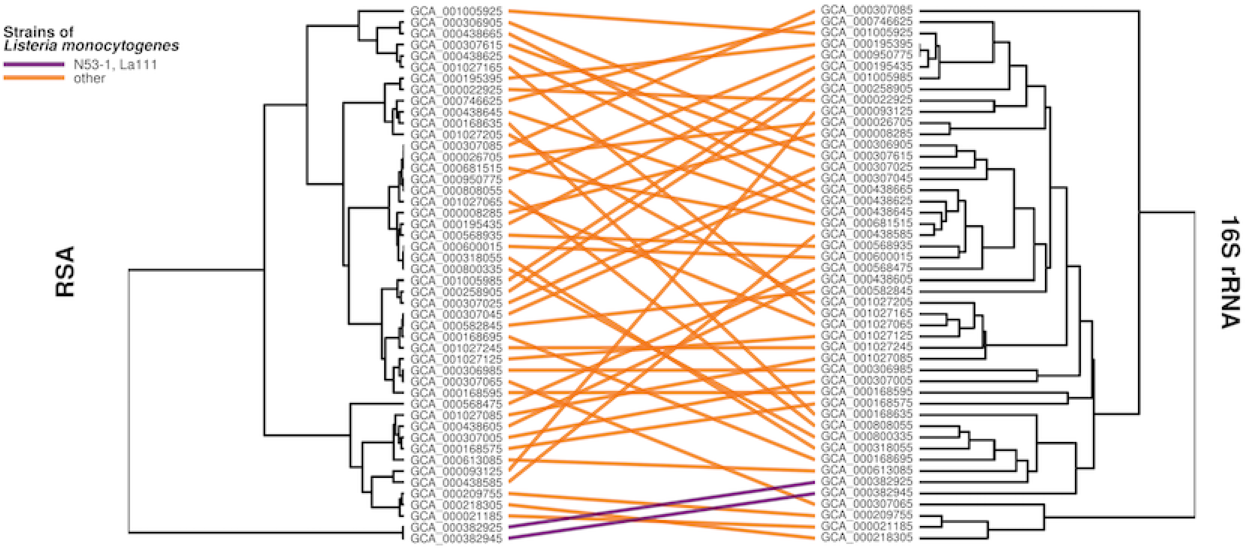

Figure S4. Hierarchical clustering of 48 strains of *Listeria monocytogenes*, by RSA method (*left*) and based on 16S rRNA gene (*right*). RSA method identifies a separate cluster of two strains: N53-1 (GCA_000382945) and La111 (GCA_000382925) (*violet*).

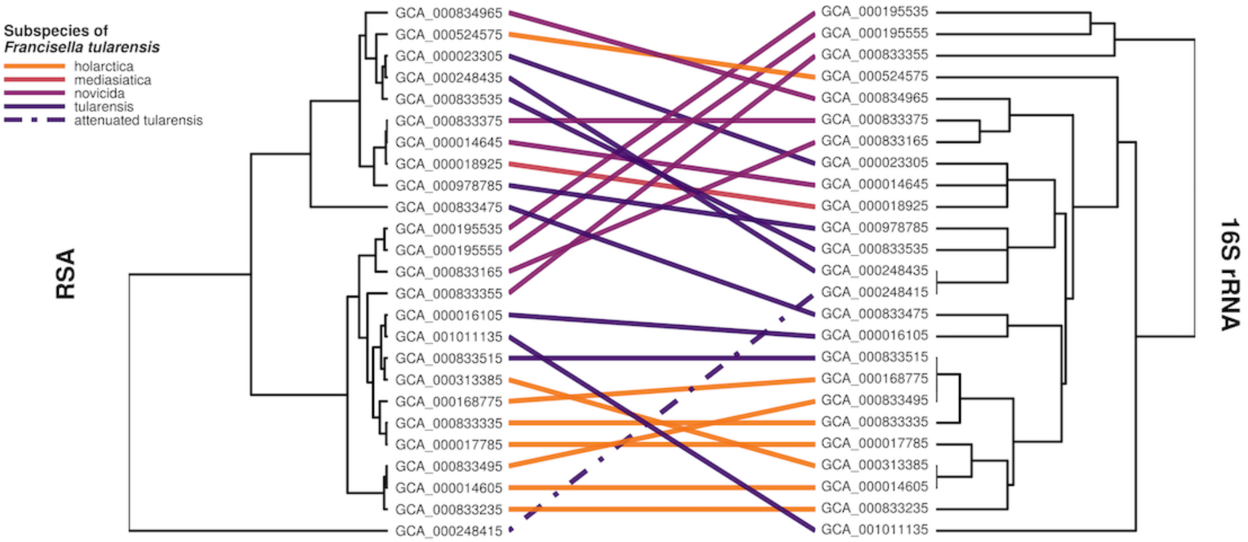

Figure S5. Hierarchical clustering of 25 strains of *Francisella tularensis*, by RSA method (*left*) and based on 16S rRNA gene (*right*). The strains belong to different subspecies: *holarctica* (*yellow*), *mediasiatica* (*orange*), *novicida* (*magenta*) and *tularensis* (*violet*). Strain TIGB03 (GCA_000248415) (*dashed violet*) is an attenuated *tularensis* strain. More precisely, it is an attenuated O-antigen mutant of virulent strain TI0902 (GCA_000248435).

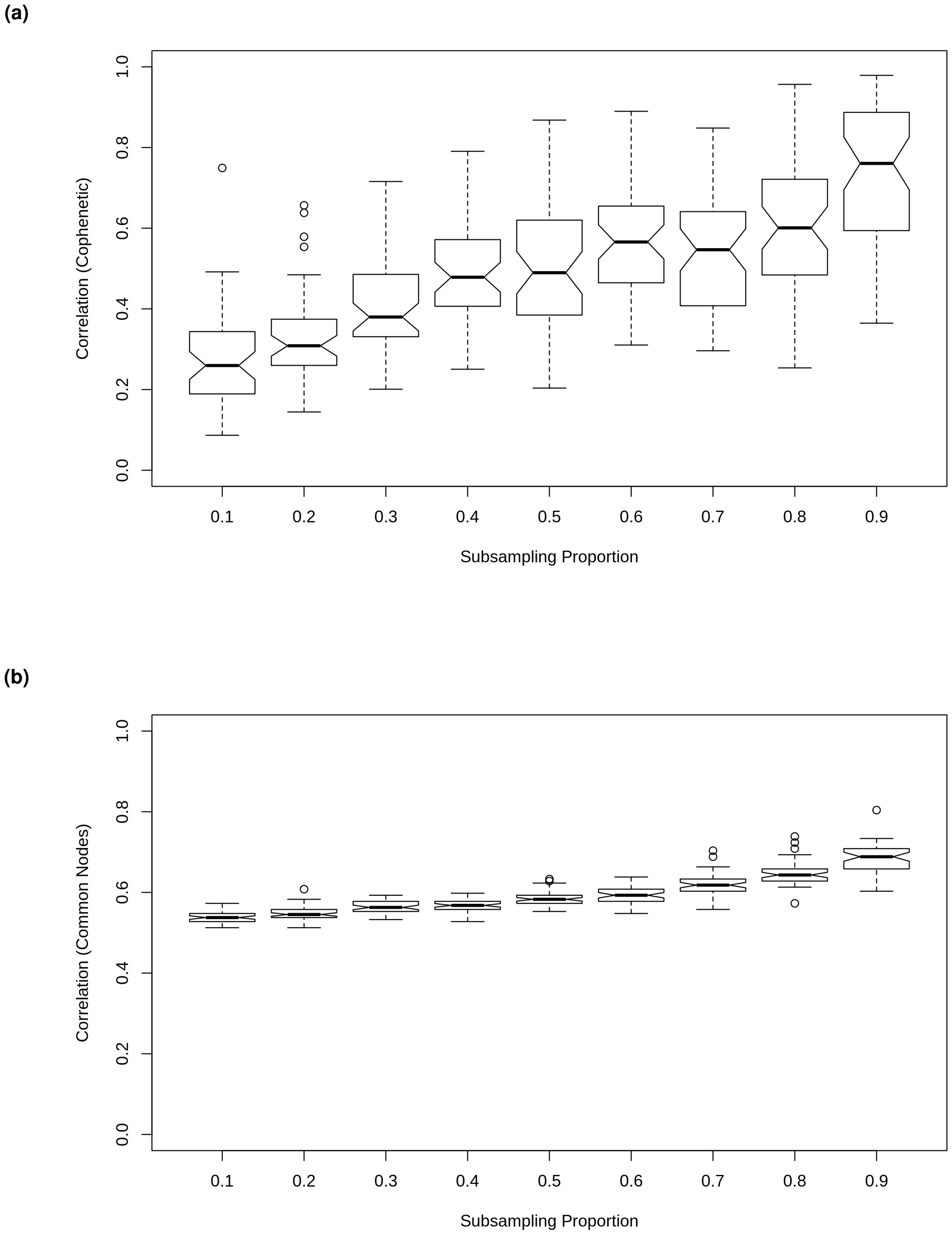

Figure S6. Correlation between full-data dendrograms and subsample-based dendrograms. Protein domains were subsampled in different proportions (*x-axis*), with 50 repeats for every proportion. For every subsampling proportion, for every repeat, 100 species were randomly selected and the correlation between a dendrograms (one calculated using all protein domains, and the other one calculated using a random subsample of domains) is calculated. Boxplot of all calculated correlations is shown in the figure. **(a)** Correlation between dendrograms is cophenetic correlation. The value of correlation is between -1 and 1. **(b)** Correlation between dendrograms is based on common nodes (i.e. nodes that have the same list of leaves). The value of correlation is between 0 and 1.

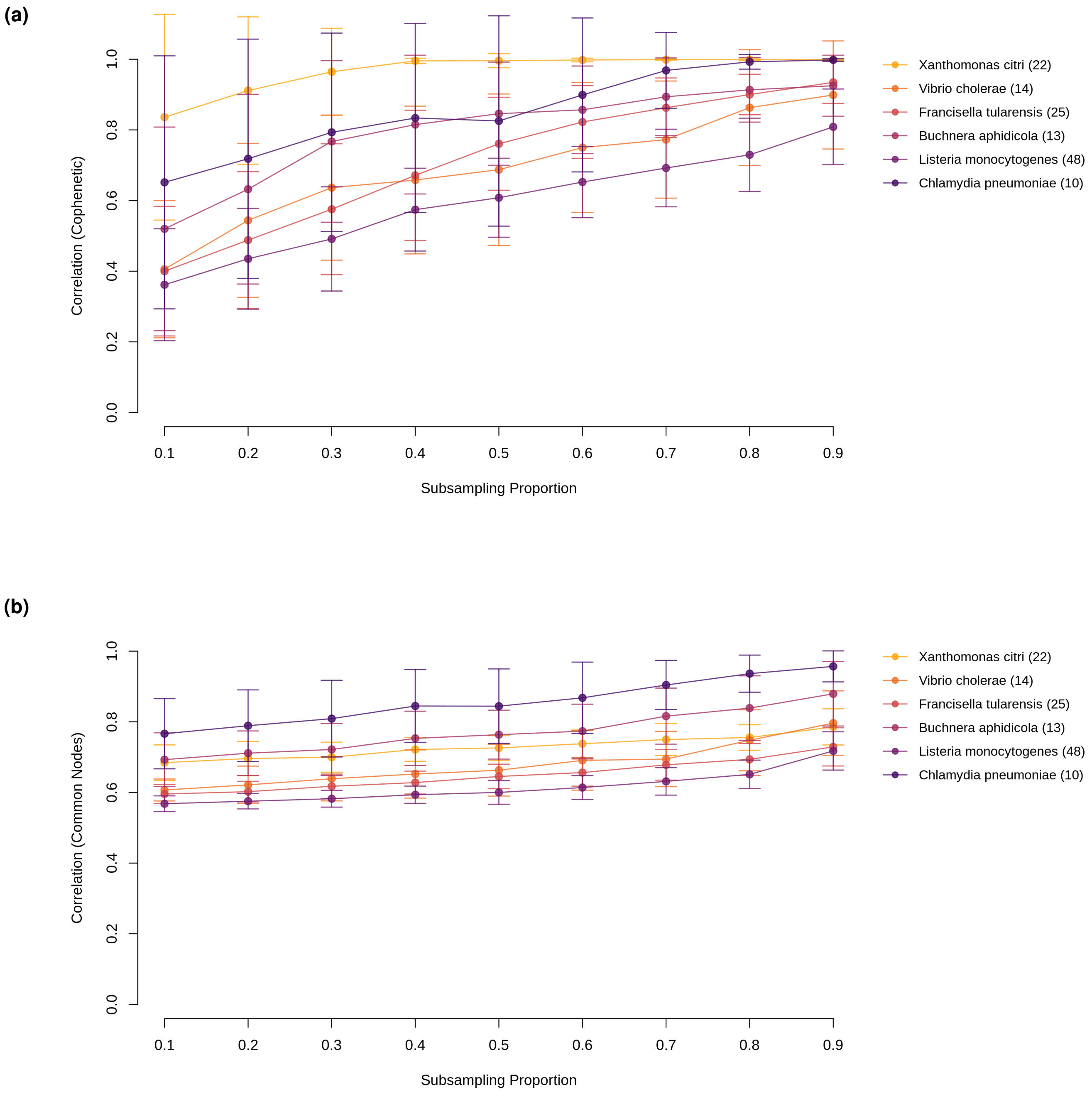

Figure S7. Correlation between a full-data dendrogram and subsample-based dendrograms, for six different bacterial species. Protein domains were subsampled in different proportions (*x-axis*), with 100 repeats for every proportion. For every subsampling proportion, mean and standard deviation of correlation (based on 100 repeats) were calculated, as shown in the figure. The number of strains within the species is indicated inside the parantheses in the legend. **(a)** Correlation between dendrograms is cophenetic correlation. The value of correlation is between -1 and 1. **(b)** Correlation between dendrograms is based on common nodes (i.e. nodes that have the same list of leaves). The value of correlation is between 0 and 1.

Data availability - accession numbers

We here provide the list of accession numbers (GeneBank IDs) of the datasets analysed in this study:

GCA_000005825 – 2.CP001878 – CP001880, GCA_000006625 – 1.AF222894, GCA_000006725 – 1.AE003849 – AE003851,
GCA_000006745 – 1.AE003852 – AE003853, GCA_000006765 – 1.AE004091, GCA_000006825 – 1.AE004439, GCA_000006845 –
1.AE004969, GCA_000006865 – 1.AE005176, GCA_000006885 – 1.AE005672, GCA_000006905 – 1.AE005673, GCA_000006945 –
1.AE006471, GCA_000006985 – 1.AE006470, GCA_000007025 – 1.AE006914, GCA_000007045 – 1.AE007317, GCA_000007085 –
1.AE008691, GCA_000007105 – 1.AE008692, GCA_000007125 – 1.AE008917 – AE008918, GCA_000007145 – 1.AE008922,
GCA_000007165 – 1.AE008923 – AE008925, GCA_000007205 – 1.AE009440, GCA_000007245 – 1.AE009442 – AE009443,
GCA_000007265 – 1.AE009948, GCA_000007285 – 1.AE009949, GCA_000007325 – 1.AE009951, GCA_000007365 – 1.AE013218,
GCA_000007385 – 1.AE013598, GCA_000007405 – 1.AE014073, GCA_000007445 – 1.AE014075, GCA_000007465 – 2.AE014133,
GCA_000007485 – 1.AE014184, GCA_000007505 – 1.AE014291 – AE014292, GCA_000007545 – 1.AE014613, GCA_000007565 –
1.AE015451, GCA_000007585 – 1.AE015924, GCA_000007605 – 1.AE015925 – AE015926, GCA_000007645 – 1.AE015929 –
AE015935, GCA_000007665 – 1.AE016822, GCA_000007685 – 1.AE016823 – AE016824, GCA_000007705 – 1.AE016825,
GCA_000007745 – 1.AE016827, GCA_000007765 – 1.AE016828 – AE016829, GCA_000007785 – 1.AE016830 – AE016833,
GCA_000007805 – 1.AE016853 – AE016855, GCA_000007865 – 1.AE016958, GCA_000007885 – 1.AE017042 – AE017046,
GCA_000007905 – 1.AE017125, GCA_000007925 – 1.AE017126, GCA_000007945 – 1.AE017143, GCA_000008005 – 1.AE017194 –
AE017195, GCA_000008025 – 1.AE017196, GCA_000008045 – 1.AE017197, GCA_000008125 – 1.AE017221 – AE017222,
GCA_000008145 – 1.AE017223 – AE017224, GCA_000008165 – 1.AE017225, GCA_000008185 – 1.AE017226, GCA_000008205 –
1.AE017243, GCA_000008225 – 1.AE017244, GCA_000008245 – 1.AE017245, GCA_000008285 – 1.AE017262, GCA_000008305 –
1.AE017263, GCA_000008325 – 1.AE017282, GCA_000008365 – 1.AE017308, GCA_000008385 – 1.AE017321, GCA_000008405 –
1.AE017332, GCA_000008445 – 1.AE017334 – AE017336, GCA_000008465 – 1.AE017340, GCA_000008485 – 1.AE017354,
GCA_000008525 – 1.AE000511, GCA_000008545 – 1.AE000512, GCA_000008585 – 1.AE000516, GCA_000008605 – 1.AE000520,
GCA_000008725 – 1.AE001273, GCA_000008745 – 1.AE001363, GCA_000008765 – 1.AE001437 – AE001438, GCA_000008785 –
1.AE001439, GCA_000008805 – 1.AE002098, GCA_000008885 – 1.BA000021, GCA_000008925 – 1.AF488831 – AF488832,
GCA_000009025 – 1.AJ965256, GCA_000009045 – 1.AL009126, GCA_000009125 – 1.AL646052 – AL646053, GCA_000009145 –
1.AL954747, GCA_000009165 – 1.AM039948 – AM039952, GCA_000009305 – 1.AM260522 – AM260523, GCA_000009365 –
1.AM286690, GCA_000009445 – 1.AM408590, GCA_000009585 – 1.AM990992 – AM990995, GCA_000009785 – 1.AP006520,
GCA_000009805 – 1.AP006618 – AP006620, GCA_000009845 – 1.AP006628, GCA_000009865 – 1.AP006716 – AP006719,
GCA_000009905 – 1.AP006840, GCA_000009925 – 1.AP006841 – AP006842, GCA_000009985 – 1.AP007255, GCA_000010005 –
1.AP007281, GCA_000010025 – 1.AP008229, GCA_000010045 – 1.AP008230, GCA_000010065 – 1.AP008231, GCA_000010085 –
1.AP008232 – AP008235, GCA_000010125 – 1.AP008934 – AP008936, GCA_000010145 – 1.AP008937, GCA_000010165 –
1.AP008955, GCA_000010185 – 1.AP008971 – AP008972, GCA_000010205 – 1.AP008981, GCA_000010225 – 1.AP009044 –
AP009045, GCA_000010265 – 1.AP009049 – AP009050, GCA_000010285 – 1.AP009152, GCA_000010305 – 1.AP009153,
GCA_000010365 – 1.AP009180, GCA_000010385 – 1.AP009240 – AP009246, GCA_000010405 – 1.AP009247, GCA_000010425 –
1.AP009256, GCA_000010445 – 1.AP009324, GCA_000010465 – 1.AP009351, GCA_000010485 – 1.AP009378 – AP009379,
GCA_000010505 – 1.AP009380, GCA_000010525 – 1.AP009384, GCA_000010565 – 1.AP009389, GCA_000010585 – 1.AP009484 –
AP009492, GCA_000010605 – 1.AP009493, GCA_000010625 – 1.AP009552, GCA_000010645 – 1.AP010656 – AP010660,
GCA_000010665 – 1.AP010904 – AP010906, GCA_000010725 – 1.AP010946 – AP010952, GCA_000010745 – 1.AP010958 –
AP010959, GCA_000010785 – 1.AP011112, GCA_000010805 – 1.AP011115 – AP011120, GCA_000010825 – 1.AP011121 –
AP011127, GCA_000010845 – 1.AP011128 – AP011134, GCA_000010865 – 1.AP011135 – AP011141, GCA_000010885 –
1.AP011142 – AP011148, GCA_000010905 – 1.AP011149 – AP011155, GCA_000010925 – 1.AP011156 – AP011162, GCA_000010945 –
1.AP011163 – AP011169, GCA_000010965 – 1.AP011170 – AP011176, GCA_000011025 – 1.AP011540, GCA_000011045 –
1.AP011548, GCA_000011145 – 1.BA000004, GCA_000011165 – 1.BA000008, GCA_000011225 – 1.BA000026, GCA_000011245 –
1.BA000028, GCA_000011285 – 1.BA000034, GCA_000011325 – 1.BA000036, GCA_000011345 – 1.BA000039, GCA_000011365 –
1.BA000040, GCA_000011385 – 1.BA000045, GCA_000011445 – 1.BX293980, GCA_000011645 – 1.CP000002, GCA_000011665 –
1.CP000003, GCA_000011685 – 1.CP000004 – CP000009, GCA_000011705 – 1.CP000010 – CP000011, GCA_000011725 –
1.CP000012, GCA_000011745 – 1.CP000016, GCA_000011825 – 1.CP000023, GCA_000011845 – 1.CP000024, GCA_000011865 –
1.CP000025, GCA_000011885 – 1.CP000026, GCA_000011905 – 1.CP000027, GCA_000011945 – 1.CP000030, GCA_000011965 –
2.CP000031 – CP000032, GCA_000011985 – 1.CP000033, GCA_000012045 – 1.CP000045 – CP000046, GCA_000012085 –
1.CP000049, GCA_000012105 – 1.CP000050, GCA_000012125 – 1.CP000051 – CP000052, GCA_000012145 – 1.CP000053 –
CP000055, GCA_000012205 – 1.CP000058 – CP000060, GCA_000012225 – 1.CP000061 – CP000065, GCA_000012245 –
1.CP000075, GCA_000012265 – 1.CP000076, GCA_000012325 – 1.CP000083, GCA_000012345 – 1.CP000084, GCA_000012365 –
1.CP000085 – CP000086, GCA_000012385 – 1.CP000087, GCA_000012405 – 1.CP000088, GCA_000012425 – 1.CP000089,
GCA_000012445 – 1.CP000094, GCA_000012465 – 1.CP000095, GCA_000012485 – 1.CP000096, GCA_000012505 – 1.CP000097,
GCA_000012525 – 1.CP000100 – CP000101, GCA_000012565 – 1.CP000107, GCA_000012585 – 1.CP000108, GCA_000012605 –

1.CP000109, GCA_000012625 − 1.CP000110, GCA_000012645 − 1.CP000111, GCA_000012665 − 1.CP000112, GCA_000012685 −
1.CP000113, GCA_000012705 − 1.CP000114, GCA_000012725 − 1.CP000115, GCA_000012745 − 1.CP000116, GCA_000012765 −
1.CP000123, GCA_000012785 − 1.CP000124 − CP000125, GCA_000012805 − 1.CP000126 − CP000127, GCA_000012825 −
1.CP000139, GCA_000012845 − 1.CP000140, GCA_000012865 − 1.CP000141, GCA_000012885 − 1.CP000142, GCA_000012925 −
1.CP000148 − CP000149, GCA_000012945 − 1.CP000150 − CP000152, GCA_000012965 − 1.CP000153, GCA_000013005 −
1.CP000157, GCA_000013045 − 1.CP000159 − CP000160, GCA_000013065 − 1.CP000227 − CP000229, GCA_000013085 −
1.CP000230 − CP000231, GCA_000013105 − 1.CP000232, GCA_000013125 − 1.CP000235, GCA_000013145 − 1.CP000236,
GCA_000013165 − 1.CP000237, GCA_000013185 − 1.CP000238, GCA_000013205 − 1.CP000239, GCA_000013225 − 1.CP000240,
GCA_000013245 − 1.CP000241 − CP000242, GCA_000013265 − 1.CP000243 − CP000244, GCA_000013285 − 1.CP000246,
GCA_000013305 − 1.CP000247, GCA_000013345 − 1.CP000249, GCA_000013365 − 1.CP000250, GCA_000013385 − 1.CP000251,
GCA_000013405 − 1.CP000252, GCA_000013425 − 1.CP000253, GCA_000013465 − 1.CP000255 − CP000258, GCA_000013565 −
1.CP000264 − CP000265, GCA_000013585 − 1.CP000266, GCA_000013605 − 1.CP000267 − CP000268, GCA_000013625 −
1.CP000269, GCA_000013645 − 1.CP000270 − CP000272, GCA_000013665 − 1.CP000282, GCA_000013685 − 1.CP000283,
GCA_000013705 − 1.CP000284, GCA_000013745 − 1.CP000301, GCA_000013765 − 1.CP000302, GCA_000013785 − 1.CP000304,
GCA_000013805 − 1.CP000305 − CP000307, GCA_000013825 − 1.CP000308 − CP000311, GCA_000013845 − 1.CP000312 −
CP000315, GCA_000013865 − 1.CP000316 − CP000318, GCA_000013885 − 1.CP000319 − CP000322, GCA_000013905 −
1.CP000323 − CP000324, GCA_000013925 − 1.CP000325, GCA_000013945 − 1.CP000348 − CP000349, GCA_000013965 −
1.CP000350 − CP000351, GCA_000013985 − 1.CP000356 − CP000357, GCA_000014005 − 1.CP000360, GCA_000014025 −
1.CP000361, GCA_000014065 − 1.CP000375 − CP000377, GCA_000014085 − 1.CP000378 − CP000380, GCA_000014105 −
1.CP000381, GCA_000014125 − 1.CP000382, GCA_000014145 − 1.CP000383, GCA_000014165 − 1.CP000384 − CP000385,
GCA_000014185 − 1.CP000386, GCA_000014205 − 1.CP000387, GCA_000014225 − 1.CP000388, GCA_000014245 − 1.CP000389 −
CP000392, GCA_000014265 − 1.CP000393, GCA_000014285 − 1.CP000394, GCA_000014305 − 1.CP000407, GCA_000014325 −
1.CP000408, GCA_000014345 − 1.CP000409, GCA_000014365 − 1.CP000410, GCA_000014385 − 1.CP000411, GCA_000014405 −
1.CP000412, GCA_000014425 − 1.CP000413, GCA_000014445 − 1.CP000414 − CP000415, GCA_000014465 − 1.CP000416 −
CP000418, GCA_000014485 − 1.CP000419 − CP000421, GCA_000014505 − 1.CP000422, GCA_000014525 − 1.CP000423 −
CP000424, GCA_000014545 − 1.CP000425 − CP000430, GCA_000014565 − 1.CP000431 − CP000434, GCA_000014585 −
1.CP000435, GCA_000014605 − 1.CP000437, GCA_000014625 − 1.CP000438, GCA_000014645 − 1.CP000439, GCA_000014665 −
1.CP000444 − CP000445, GCA_000014685 − 1.CP000446, GCA_000014705 − 1.CP000447, GCA_000014725 − 1.CP000448,
GCA_000014745 − 1.CP000449, GCA_000014765 − 1.CP000450 − CP000452, GCA_000014785 − 1.CP000453, GCA_000014805 −
1.CP000462, GCA_000014825 − 1.CP000463, GCA_000014865 − 1.CP000471, GCA_000014905 − 1.CP000473, GCA_000014925 −
1.CP000474 − CP000476, GCA_000014965 − 1.CP000478, GCA_000014985 − 1.CP000479, GCA_000015005 − 1.CP000480,
GCA_000015025 − 1.CP000481, GCA_000015045 − 1.CP000482 − CP000484, GCA_000015065 − 1.CP000485 − CP000486,
GCA_000015085 − 1.CP000487, GCA_000015105 − 1.CP000488, GCA_000015125 − 1.CP000492, GCA_000015165 − 1.CP000494 −
CP000495, GCA_000015185 − 1.CP000503, GCA_000015245 − 1.CP000507, GCA_000015265 − 1.CP000508 − CP000509,
GCA_000015285 − 1.CP000510, GCA_000015305 − 1.CP000511, GCA_000015325 − 1.CP000512, GCA_000015345 − 1.CP000513,
GCA_000015365 − 1.CP000514 − CP000516, GCA_000015385 − 1.CP000517, GCA_000015405 − 1.CP000518 − CP000520,
GCA_000015425 − 1.CP000521 − CP000523, GCA_000015445 − 1.CP000524, GCA_000015485 − 1.CP000527 − CP000528,
GCA_000015505 − 1.CP000529 − CP000537, GCA_000015545 − 1.CP000539 − CP000541, GCA_000015565 − 1.CP000542 −
CP000543, GCA_000015585 − 1.CP000544, GCA_000015605 − 1.CP000545 − CP000546, GCA_000015625 − 1.CP000547 −
CP000548, GCA_000015645 − 1.CP000551, GCA_000015665 − 1.CP000552, GCA_000015685 − 1.CP000553, GCA_000015705 −
1.CP000554, GCA_000015725 − 1.CP000555 − CP000556, GCA_000015745 − 1.CP000557 − CP000558, GCA_000015785 −
1.CP000560, GCA_000015845 − 1.CP000563 − CP000567, GCA_000015865 − 1.CP000568, GCA_000015885 − 1.CP000569,
GCA_000015905 − 1.CP000570 − CP000571, GCA_000015925 − 1.CP000572 − CP000573, GCA_000015965 − 1.CP000576,
GCA_000015985 − 1.CP000577 − CP000579, GCA_000016005 − 1.CP000580, GCA_000016085 − 1.CP000607, GCA_000016105 −
1.CP000608, GCA_000016145 − 1.CP000611, GCA_000016165 − 1.CP000612, GCA_000016205 − 1.CP000614 − CP000621,
GCA_000016245 − 1.CP000626 − CP000627, GCA_000016265 − 1.CP000628 − CP000632, GCA_000016285 − 1.CP000633 −
CP000639, GCA_000016305 − 1.CP000647 − CP000652, GCA_000016325 − 1.CP000653 − CP000654, GCA_000016345 −
1.CP000655, GCA_000016365 − 1.CP000656 − CP000659, GCA_000016405 − 1.CP000661 − CP000666, GCA_000016425 −
1.CP000667, GCA_000016445 − 1.CP000668 − CP000670, GCA_000016465 − 1.CP000671, GCA_000016485 − 1.CP000672,
GCA_000016505 − 1.CP000673 − CP000674, GCA_000016545 − 1.CP000679, GCA_000016565 − 1.CP000680, GCA_000016585 −
1.CP000681, GCA_000016625 − 1.CP000683 − CP000684, GCA_000016645 − 1.CP000685, GCA_000016665 − 1.CP000686,
GCA_000016685 − 1.CP000687, GCA_000016705 − 1.CP000688, GCA_000016725 − 1.CP000689 − CP000697, GCA_000016745 −
1.CP000698, GCA_000016765 − 1.CP000699 − CP000701, GCA_000016785 − 1.CP000702, GCA_000016805 − 1.CP000703 −
CP000704, GCA_000016825 − 1.CP000705, GCA_000016845 − 1.CP000708 − CP000709, GCA_000016865 − 1.CP000712,
GCA_000016885 − 1.CP000713 − CP000715, GCA_000016905 − 1.CP000716, GCA_000016945 − 1.CP000718 − CP000720,

GCA_000016965 − 1.CP000721, GCA_000016985 − 1.CP000724, GCA_000017005 − 1.CP000725, GCA_000017025 − 1.CP000726,
GCA_000017045 − 1.CP000727, GCA_000017065 − 1.CP000728 − CP000729, GCA_000017125 − 1.CP000736 − CP000737,
GCA_000017145 − 1.CP000738 − CP000741, GCA_000017205 − 1.CP000744, GCA_000017245 − 1.CP000746, GCA_000017265 −
1.CP000747 − CP000748, GCA_000017285 − 1.CP000749, GCA_000017305 − 1.CP000750 − CP000752, GCA_000017325 −
1.CP000753 − CP000754, GCA_000017405 − 1.CP000758 − CP000763, GCA_000017425 − 1.CP000764 − CP000765, GCA_000017445 −
3.CP000766, GCA_000017465 − 1.CP000767, GCA_000017485 − 1.CP000768, GCA_000017505 − 1.CP000769, GCA_000017525 −
1.CP000770, GCA_000017545 − 1.CP000771, GCA_000017565 − 1.CP000774, GCA_000017585 − 1.CP000775 − CP000776,
GCA_000017605 − 1.CP000777 − CP000779, GCA_000017645 − 1.CP000781 − CP000782, GCA_000017665 − 1.CP000783 −
CP000785, GCA_000017685 − 1.CP000786 − CP000788, GCA_000017705 − 1.CP000789 − CP000791, GCA_000017725 −
1.CP000792 − CP000794, GCA_000017745 − 1.CP000795 − CP000801, GCA_000017765 − 1.CP000802, GCA_000017785 −
1.CP000803, GCA_000017805 − 1.CP000804, GCA_000017845 − 1.CP000806 − CP000811, GCA_000017865 − 1.CP000812,
GCA_000017885 − 1.CP000813, GCA_000017905 − 1.CP000814, GCA_000017965 − 1.CP000817 − CP000818, GCA_000017985 −
1.CP000819, GCA_000018005 − 1.CP000820, GCA_000018025 − 1.CP000821, GCA_000018045 − 1.CP000822 − CP000824,
GCA_000018065 − 1.CP000825, GCA_000018085 − 1.CP000826 − CP000827, GCA_000018145 − 1.CP000830 − CP000835,
GCA_000018185 − 1.CP000837, GCA_000018205 − 1.CP000847, GCA_000018225 − 1.CP000848, GCA_000018245 − 1.CP000849,
GCA_000018265 − 1.CP000850, GCA_000018285 − 1.CP000851, GCA_000018325 − 1.CP000853, GCA_000018385 − 1.CP000857 −
CP000858, GCA_000018425 − 1.CP000860, GCA_000018445 − 1.CP000863 − CP000865, GCA_000018505 − 1.CP000868 −
CP000871, GCA_000018525 − 1.CP000872 − CP000873, GCA_000018565 − 1.CP000875 − CP000877, GCA_000018605 −
1.CP000879, GCA_000018625 − 1.CP000880, GCA_000018665 − 1.CP000884, GCA_000018685 − 1.CP000885, GCA_000018705 −
1.CP000886, GCA_000018725 − 1.CP000887 − CP000888, GCA_000018745 − 1.CP000889 − CP000890, GCA_000018765 −
1.CP000891 − CP000894, GCA_000018785 − 1.CP000896, GCA_000018805 − 1.CP000900 − CP000902, GCA_000018825 −
1.CP000903 − CP000907, GCA_000018845 − 1.CP000908, GCA_000018865 − 1.CP000909, GCA_000018885 − 1.CP000910,
GCA_000018905 − 1.CP000911 − CP000912, GCA_000018925 − 1.CP000915, GCA_000018945 − 1.CP000916, GCA_000018965 −
1.CP000918, GCA_000018985 − 1.CP000919, GCA_000019005 − 1.CP000920, GCA_000019025 − 1.CP000921, GCA_000019045 −
1.CP000922, GCA_000019065 − 1.CP000923, GCA_000019085 − 1.CP000924, GCA_000019125 − 1.CP000926, GCA_000019145 −
1.CP000927 − CP000929, GCA_000019165 − 1.CP000930, GCA_000019185 − 1.CP000931, GCA_000019205 − 1.CP000932 −
CP000933, GCA_000019225 − 1.CP000934, GCA_000019265 − 1.CP000936, GCA_000019285 − 1.CP000937 − CP000938,
GCA_000019305 − 1.CP000939 − CP000940, GCA_000019325 − 1.CP000941, GCA_000019345 − 1.CP000942, GCA_000019365 −
1.CP000943 − CP000945, GCA_000019385 − 1.CP000946, GCA_000019405 − 1.CP000947, GCA_000019445 − 1.CP000949,
GCA_000019465 − 1.CP000950, GCA_000019485 − 1.CP000951 − CP000957, GCA_000019505 − 1.CP000958 − CP000960,
GCA_000019525 − 1.CP000961, GCA_000019545 − 1.CP000962 − CP000963, GCA_000019565 − 1.CP000964 − CP000966,
GCA_000019585 − 2.CP000967, GCA_000019625 − 1.CP000969, GCA_000019645 − 1.CP000970 − CP000974, GCA_000019665 −
1.CP000975, GCA_000019685 − 1.CP000976 − CP000992, GCA_000019705 − 1.CP000993 − CP001000, GCA_000019725 −
1.CP001001 − CP001009, GCA_000019745 − 1.CP001010, GCA_000019765 − 1.CP001011 − CP001012, GCA_000019785 −
1.CP001013, GCA_000019825 − 1.CP001015, GCA_000019845 − 1.CP001016 − CP001018, GCA_000019865 − 1.CP001019,
GCA_000019885 − 1.CP001020 − CP001021, GCA_000019905 − 1.CP001022 − CP001024, GCA_000019925 − 1.CP001025 −
CP001028, GCA_000019945 − 1.CP001029 − CP001031, GCA_000019965 − 1.CP001032, GCA_000019985 − 1.CP001033,
GCA_000020005 − 1.CP001034 − CP001036, GCA_000020025 − 1.CP001037 − CP001042, GCA_000020045 − 1.CP001043 −
CP001046, GCA_000020065 − 1.CP001047, GCA_000020085 − 1.CP001048 − CP001049, GCA_000020105 − 1.CP001050 −
CP001051, GCA_000020125 − 1.CP001052 − CP001054, GCA_000020145 − 1.CP001055, GCA_000020165 − 1.CP001056 −
CP001057, GCA_000020185 − 1.CP001058 − CP001063, GCA_000020205 − 1.CP001068 − CP001070, GCA_000020225 −
1.CP001071, GCA_000020245 − 1.CP001072, GCA_000020285 − 1.CP001078, GCA_000020305 − 1.CP001079, GCA_000020325 −
1.CP001080, GCA_000020345 − 1.CP001081 − CP001083, GCA_000020365 − 1.CP001087 − CP001088, GCA_000020385 −
1.CP001089 − CP001090, GCA_000020405 − 1.CP001091 − CP001094, GCA_000020425 − 1.CP001095, GCA_000020445 −
1.CP001096, GCA_000020465 − 1.CP001097, GCA_000020485 − 1.CP001098, GCA_000020505 − 1.CP001099, GCA_000020525 −
1.CP001100, GCA_000020545 − 1.CP001101, GCA_000020565 − 1.CP001102, GCA_000020585 − 3.CP001103, GCA_000020605 −
1.CP001107, GCA_000020625 − 1.CP001108 − CP001109, GCA_000020645 − 1.CP001110, GCA_000020665 − 1.CP001111,
GCA_000020685 − 1.CP001114 − CP001117, GCA_000020705 − 1.CP001118 − CP001120, GCA_000020725 − 1.CP001124,
GCA_000020745 − 1.CP001125 − CP001127, GCA_000020765 − 1.CP001129, GCA_000020785 − 1.CP001130, GCA_000020805 −
1.CP001131, GCA_000020825 − 1.CP001132, GCA_000020865 − 1.CP001135 − CP001136, GCA_000020885 − 1.CP001137 −
CP001138, GCA_000020925 − 1.CP001143 − CP001144, GCA_000020965 − 1.CP001146, GCA_000020985 − 1.CP001147,
GCA_000021005 − 1.CP001150 − CP001153, GCA_000021025 − 1.CP001154, GCA_000021045 − 1.CP001157, GCA_000021065 −
1.CP001158, GCA_000021085 − 1.CP001161, GCA_000021125 − 1.CP001163 − CP001165, GCA_000021145 − 1.CP001172,
GCA_000021165 − 1.CP001173 − CP001174, GCA_000021185 − 1.CP001175, GCA_000021205 − 1.CP001176, GCA_000021225 −
1.CP001177 − CP001181, GCA_000021245 − 1.CP001182 − CP001183, GCA_000021265 − 1.CP001184, GCA_000021285 −

1.CP001185, GCA_000021305 – 1.CP001186 – CP001188, GCA_000021325 – 1.CP001189 – CP001190, GCA_000021345 –
1.CP001191 – CP001195, GCA_000021365 – 1.CP001196, GCA_000021385 – 1.CP001197, GCA_000021405 – 1.CP001198 –
CP001212, GCA_000021425 – 1.CP001213, GCA_000021445 – 1.CP001214 – CP001216, GCA_000021465 – 1.CP001217 –
CP001218, GCA_000021485 – 1.CP001219, GCA_000021505 – 1.CP001226, GCA_000021525 – 1.CP001227 – CP001228,
GCA_000021545 – 1.CP001229, GCA_000021565 – 1.CP001230 – CP001231, GCA_000021605 – 1.CP001233 – CP001234,
GCA_000021625 – 1.CP001235 – CP001236, GCA_000021645 – 1.CP001251, GCA_000021665 – 1.CP001252 – CP001255,
GCA_000021685 – 1.CP001275 – CP001276, GCA_000021725 – 1.CP001279, GCA_000021745 – 1.CP001280, GCA_000021765 –
1.CP001281 – CP001282, GCA_000021785 – 1.CP001283 – CP001286, GCA_000021805 – 1.CP001287 – CP001290, GCA_000021825 –
1.CP001291 – CP001297, GCA_000021845 – 1.CP001298 – CP001300, GCA_000021865 – 1.CP001312 – CP001313, GCA_000021885 –
1.CP001321, GCA_000021905 – 1.CP001322, GCA_000021925 – 1.CP001336, GCA_000021945 – 1.CP001337, GCA_000021985 –
1.CP001339, GCA_000022005 – 1.CP001340, GCA_000022025 – 1.CP001341 – CP001343, GCA_000022045 – 1.CP001344 –
CP001347, GCA_000022065 – 1.CP001348, GCA_000022085 – 1.CP001349 – CP001356, GCA_000022105 – 1.CP001360,
GCA_000022125 – 1.CP001358, GCA_000022145 – 1.CP001359, GCA_000022185 – 1.CP001364, GCA_000022225 – 1.CP001368 –
CP001369, GCA_000022245 – 1.CP001383 – CP001388, GCA_000022265 – 1.CP001390, GCA_000022285 – 1.CP001391,
GCA_000022305 – 1.CP001392, GCA_000022325 – 1.CP001393 – CP001395, GCA_000022345 – 1.CP001396, GCA_000022505 –
1.CP001406 – CP001407, GCA_000022525 – 1.CP001431, GCA_000022565 – 1.CP001472, GCA_000022585 – 1.CP001485 –
CP001486, GCA_000022625 – 1.CP001488 – CP001489, GCA_000022645 – 2.CP001503 – CP001508, GCA_000022665 –
2.CP001509, GCA_000022685 – 1.CP001510 – CP001514, GCA_000022705 – 1.CP001515, GCA_000022725 – 1.CP001562 –
CP001563, GCA_000022745 – 1.CP001578 – CP001579, GCA_000022765 – 1.CP001581, GCA_000022785 – 1.CP001584,
GCA_000022845 – 1.CP001593 – CP001596, GCA_000022865 – 1.CP001597 – CP001599, GCA_000022885 – 2.CP001600,
GCA_000022925 – 1.CP001604, GCA_000022945 – 1.CP001605, GCA_000022965 – 1.CP001606, GCA_000022985 – 1.CP001607,
GCA_000023005 – 1.CP001612 – CP001613, GCA_000023025 – 1.CP001614, GCA_000023045 – 1.CP001615, GCA_000023065 –
1.CP001616, GCA_000023085 – 1.CP001617, GCA_000023105 – 1.CP001618, GCA_000023125 – 1.CP001619, GCA_000023145 –
1.CP001620, GCA_000023185 – 1.CP001622 – CP001627, GCA_000023205 – 1.CP001628, GCA_000023225 – 1.CP001629,
GCA_000023245 – 1.CP001630, GCA_000023265 – 1.CP001631, GCA_000023285 – 1.CP001632, GCA_000023305 – 1.CP001633,
GCA_000023325 – 1.CP001634, GCA_000023345 – 1.CP001635 – CP001636, GCA_000023365 – 1.CP001637, GCA_000023385 –
1.CP001638 – CP001640, GCA_000023405 – 1.CP001643, GCA_000023425 – 1.CP001644 – CP001648, GCA_000023445 –
1.CP001649, GCA_000023465 – 1.CP001650, GCA_000023545 – 1.CP001654, GCA_000023565 – 1.CP001655, GCA_000023585 –
1.CP001656, GCA_000023605 – 1.CP001657, GCA_000023625 – 1.CP001658, GCA_000023645 – 1.CP001661, GCA_000023665 –
1.CP001665, GCA_000023705 – 1.CP001672, GCA_000023725 – 1.CP001673, GCA_000023745 – 1.CP001674 – CP001676,
GCA_000023765 – 2.CP001677, GCA_000023785 – 1.CP001678 – CP001679, GCA_000023805 – 1.CP001680, GCA_000023825 –
1.CP001681, GCA_000023845 – 1.CP001682, GCA_000023865 – 1.CP001683, GCA_000023885 – 1.CP001684, GCA_000023905 –
1.CP001685, GCA_000023925 – 1.CP001686, GCA_000024005 – 1.CP001699, GCA_000024025 – 1.CP001700, GCA_000024045 –
1.CP001701 – CP001705, GCA_000024065 – 1.CP001706, GCA_000024085 – 1.CP001707, GCA_000024105 – 1.CP001708 –
CP001709, GCA_000024125 – 1.CP001712, GCA_000024145 – 1.CP001713 – CP001714, GCA_000024165 – 1.CP001715 –
CP001718, GCA_000024205 – 1.CP001720, GCA_000024225 – 1.CP001721, GCA_000024245 – 1.CP001722 – CP001725,
GCA_000024265 – 1.CP001726, GCA_000024285 – 1.CP001727 – CP001730, GCA_000024325 – 1.CP001734 – CP001735,
GCA_000024345 – 1.CP001736, GCA_000024365 – 1.CP001737, GCA_000024385 – 1.CP001738, GCA_000024405 – 1.CP001739 –
CP001741, GCA_000024425 – 1.CP001743, GCA_000024445 – 1.CP001750, GCA_000024465 – 1.CP001751, GCA_000024505 –
1.CP001759, GCA_000024525 – 1.CP001769 – CP001777, GCA_000024545 – 1.CP001778, GCA_000024565 – 1.CP001779 –
CP001780, GCA_000024585 – 1.CP001781 – CP001784, GCA_000024605 – 1.CP001785 – CP001786, GCA_000024645 –
1.CP001790, GCA_000024665 – 1.CP001792, GCA_000024685 – 1.CP001793, GCA_000024705 – 1.CP001794 – CP001795,
GCA_000024725 – 1.CP001798 – CP001799, GCA_000024765 – 1.CP001801, GCA_000024785 – 1.CP001802 – CP001803,
GCA_000024805 – 1.CP001804, GCA_000024825 – 1.CP001805 – CP001806, GCA_000024845 – 1.CP001807 – CP001808,
GCA_000024865 – 1.CP001814 – CP001815, GCA_000024885 – 1.CP001816, GCA_000024905 – 1.CP001818, GCA_000024925 –
1.CP001819, GCA_000024945 – 1.CP001820, GCA_000024965 – 1.CP001821 – CP001822, GCA_000024985 – 1.CP001823 –
CP001824, GCA_000025005 – 1.CP001825 – CP001826, GCA_000025025 – 1.CP001827, GCA_000025045 – 1.CP001834 –
CP001835, GCA_000025065 – 1.CP001836, GCA_000025085 – 1.CP001837, GCA_000025105 – 1.CP001839, GCA_000025125 –
1.CP001842, GCA_000025165 – 1.CP001846 – CP001847, GCA_000025185 – 1.CP001848, GCA_000025205 – 1.CP001849,
GCA_000025245 – 1.CP001853, GCA_000025265 – 1.CP001854, GCA_000025305 – 1.CP001859, GCA_000025345 – 1.CP001867,
GCA_000025365 – 1.CP001872, GCA_000025385 – 1.CP001873, GCA_000025405 – 2.CP001875, GCA_000025425 – 1.CP001876 –
CP001877, GCA_000025465 – 1.CP001891, GCA_000025485 – 1.CP001896 – CP001898, GCA_000025545 – 1.CP001905 –
CP001906, GCA_000025565 – 1.CP001918 – CP001920, GCA_000025585 – 1.CP001924, GCA_000025605 – 1.CP001931,
GCA_000025645 – 1.CP001936, GCA_000025705 – 1.CP001965, GCA_000025725 – 1.CP001968, GCA_000025745 – 1.CP001969,
GCA_000025765 – 1.CP001977, GCA_000025785 – 1.CP001981, GCA_000025845 – 1.CP001991, GCA_000025885 – 1.CP001997,

GCA_000025905 − 1.CP001998, GCA_000025925 − 1.CP002006, GCA_000025945 − 1.CR522870 − CR522872, GCA_000025965 − 1.CR555306 − CR555308, GCA_000026065 − 1.CR936503, GCA_000026085 − 1.CR954246 − CR954247, GCA_000026105 − 1.CT573326, GCA_000026125 − 1.CU207211, GCA_000026145 − 1.CU234118, GCA_000026205 − 1.CU469464, GCA_000026245 − 1.CU928145, GCA_000026265 − 1.CU928160, GCA_000026285 − 1.CU928161, GCA_000026325 − 1.CU928163, GCA_000026345 − 1.CU928164, GCA_000026485 − 1.FM177140, GCA_000026505 − 1.FM179322, GCA_000026525 − 1.FM179323 − FM179324, GCA_000026645 − 1.FM209186, GCA_000026705 − 1.FM242711, GCA_000026965 − 1.FM999788, GCA_000027065 − 2.FN543093 − FN543096, GCA_000027185 − 1.FN597254, GCA_000027225 − 1.FN667741, GCA_000027305 − 1.L42023, GCA_000027325 − 1.L43967, GCA_000027345 − 1.U00089, GCA_000039765 − 1.AE016795 − AE016796, GCA_000046845 − 1.CR543861, GCA_000050405 − 1.CR925677, GCA_000050425 − 1.CR925678, GCA_000055785 − 1.CP000285, GCA_000056065 − 1.CR954253, GCA_000060285 − 1.AM263198, GCA_000060345 − 1.CU207366, GCA_000061505 − 1.AM406670, GCA_000063505 − 1.CT971583, GCA_000063525 − 1.CT978603, GCA_000063605 − 1.CU179680, GCA_000064305 − 2.AM398681, GCA_000069245 − 1.CU459137 − CU459141, GCA_000069925 − 1.AM422018, GCA_000069945 − 1.AM942444, GCA_000083545 − 1.FP103042 − FP103044, GCA_000085865 − 1.FP236530, GCA_000089865 − 1.FP671138, GCA_000090405 − 1.FP565810 − FP565814, GCA_000091005 − 1.AP010953 − AP010957, GCA_000091125 − 1.AP010803 − AP010807, GCA_000091325 − 1.AP011177, GCA_000091345 − 1.FM991728, GCA_000091465 − 1.FM954972 − FM954973, GCA_000091545 − 1.AP008226 − AP008228, GCA_000091565 − 1.FN434113 − FN434114, GCA_000091645 − 1.AP010655, GCA_000091725 − 1.FN806773, GCA_000091785 − 1.FN650140 − FN650141, GCA_000092025 − 1.AE007869 − AE007872, GCA_000092105 − 1.CP001744 − CP001745, GCA_000092125 − 1.CP002042 − CP002044, GCA_000092165 − 1.CP001903 − CP001904, GCA_000092205 − 1.CP001940, GCA_000092225 − 1.CP001966 − CP001967, GCA_000092245 − 1.CP001999, GCA_000092265 − 1.CP002005, GCA_000092285 − 1.CP002008, GCA_000092325 − 1.CP002010, GCA_000092345 − 1.CP002011 − CP002012, GCA_000092365 − 1.CP002045, GCA_000092385 − 1.CP002047, GCA_000092405 − 1.CP002048, GCA_000092425 − 1.CP002049, GCA_000092445 − 1.CP002050, GCA_000092485 − 1.CP002052 − CP002053, GCA_000092505 − 1.CP001753 − CP001758, GCA_000092545 − 1.CP000675, GCA_000092565 − 1.AE010300 − AE010301, GCA_000092585 − 1.AE015450, GCA_000092605 − 1.CP002021 − CP002023, GCA_000092625 − 1.CP001828, GCA_000092645 − 1.CP001874, GCA_000092665 − 1.CP001886, GCA_000092685 − 1.CP001887, GCA_000092705 − 1.CP001888, GCA_000092725 − 1.CP001889, GCA_000092745 − 1.CP001890, GCA_000092765 − 1.CP001892, GCA_000092785 − 1.CP001928 − CP001929, GCA_000092805 − 1.CP001930, GCA_000092825 − 1.CP001958, GCA_000092845 − 1.CP001959, GCA_000092865 − 1.CP001964, GCA_000092885 − 1.CP002013 − CP002016, GCA_000092905 − 1.CP002017, GCA_000092925 − 1.CP002026, GCA_000092945 − 1.CP002028, GCA_000092965 − 1.CP002032, GCA_000092985 − 1.CP002040 − CP002041, GCA_000093005 − 1.CP002054 − CP002055, GCA_000093025 − 1.CP002056, GCA_000093065 − 1.CP001085 − CP001086, GCA_000093085 − 1.CP001791, GCA_000093125 − 2.CP001602 − CP001603, GCA_000093145 − 2.CP001220, GCA_000093165 − 1.CP001429 − CP001430, GCA_000093185 − 1.CP001582 − CP001583, GCA_000143085 − 1.CP002086 − CP002088, GCA_000143145 − 1.CP002083, GCA_000143165 − 1.CP002084, GCA_000143205 − 1.CP002094, GCA_000143225 − 1.CP002039, GCA_000143435 − 1.CP002034 − CP002037, GCA_000143605 − 1.CP001746 − CP001749, GCA_000143685 − 1.CP001666, GCA_000143705 − 1.CP002097, GCA_000143725 − 1.CP002025, GCA_000143845 − 1.CP002106, GCA_000143865 − 1.CP002107, GCA_000143945 − 1.CP002077, GCA_000143965 − 1.CP002085, GCA_000143985 − 1.CP002116, GCA_000144405 − 1.CP002122 − CP002123, GCA_000144605 − 1.CP002102, GCA_000144625 − 1.CP002109, GCA_000144645 − 1.CP002131, GCA_000144675 − 1.CP001829, GCA_000144695 − 1.CP002105, GCA_000144935 − 2.CP001809, GCA_000144955 − 1.CP002114 − CP002115, GCA_000145035 − 1.CP001810 − CP001813, GCA_000145215 − 1.CP002164, GCA_000145235 − 1.CP002162, GCA_000145255 − 1.CP002159, GCA_000145275 − 1.CP002160, GCA_000145595 − 1.CP002120, GCA_000145615 − 1.CP002171, GCA_000145705 − 1.CP002170, GCA_000146165 − 2.AE014299 − AE014300, GCA_000146185 − 1.CP001104 − CP001106, GCA_000146305 − 1.CP002154 − CP002155, GCA_000146505 − 1.CP002158, GCA_000146565 − 1.CP002183, GCA_000147015 − 1.CP002161, GCA_000147035 − 1.CP002163, GCA_000147075 − 1.CP001698, GCA_000147335 − 1.CP002198 − CP002204, GCA_000147355 − 1.CP002205, GCA_000147695 − 3.CP002991, GCA_000147715 − 3.CP003532 − CP003533, GCA_000147735 − 3.CP002767 − CP002769, GCA_000147775 − 3.CP002740 − CP002742, GCA_000147795 − 3.CP002781 − CP002785, GCA_000147815 − 3.CP002994 − CP002996, GCA_000147835 − 3.CP002798 − CP002800, GCA_000147855 − 3.CP002516 − CP002518, GCA_000148405 − 1.CP002165 − CP002166, GCA_000148425 − 1.CP002210, GCA_000148605 − 1.CP002167 − CP002168, GCA_000148625 − 1.CP001995, GCA_000148645 − 1.CP002209, GCA_000148665 − 1.CP002184, GCA_000148685 − 1.CP002217 − CP002218, GCA_000148705 − 1.CP001900, GCA_000148815 − 2.CP002222 − CP002223, GCA_000148855 − 1.CP002073, GCA_000148875 − 1.CP002074 − CP002075, GCA_000148895 − 1.CP002076, GCA_000148915 − 1.CP002071 − CP002072, GCA_000152065 − 1.CP002251, GCA_000152245 − 2.CP002273, GCA_000152825 − 2.CP002156, GCA_000152925 − 3.CP009301, GCA_000152945 − 2.CP004349, GCA_000153165 − 2.CP002157, GCA_000153405 − 2.CP004404, GCA_000153485 − 2.CP003879, GCA_000153685 − 2.CP001664, GCA_000154585 − 2.CP001662, GCA_000154605 − 2.CP001976, GCA_000154745 − 2.CP002972 − CP002975, GCA_000154765 − 2.CP002976 − CP002979, GCA_000154785 − 2.CP002623 − CP002626, GCA_000155515 − 2.CP002391 − CP002393, GCA_000155675 − 2.CP003740 − CP003741, GCA_000155735 − 2.CP003742 − CP003744, GCA_000156855 − 2.CP003909 − CP003910, GCA_000157355 − 2.CP004856, GCA_000157895 − 2.CP006835 − CP006836, GCA_000158275 −

2.CP007062 – CP007063, GCA_000159155 – 2.CP002104, GCA_000159455 – 2.CP002844 – CP002848, GCA_000159535 – 2.CP002110 – CP002111, GCA_000160335 – 2.CP002388 – CP002389, GCA_000161795 – 2.CP006882, GCA_000162235 – 2.CP003700, GCA_000163055 – 2.CP003666 – CP003667, GCA_000163615 – 2.CP003496, GCA_000163895 – 2.CP002390, GCA_000163915 – 2.CP007064 – CP007066, GCA_000164675 – 2.CP002843, GCA_000164695 – 2.CP002280, GCA_000164865 – 1.CP002272, GCA_000164885 – 1.CP002224 – CP002226, GCA_000164905 – 1.CP002221, GCA_000164965 – 1.CP002220, GCA_000164985 – 2.CP002213 – CP002214, GCA_000165465 – 1.CP002175, GCA_000165485 – 1.CP002271, GCA_000165505 – 1.CP002281 – CP002283, GCA_000165575 – 1.CP002276, GCA_000165715 – 3.CP002546, GCA_000165775 – 3.CP003799, GCA_000165815 – 1.CP002124 – CP002129, GCA_000165835 – 1.CP002287 – CP002289, GCA_000165905 – 1.CP001840, GCA_000165925 – 1.CP002207, GCA_000166055 – 1.CP002292, GCA_000166075 – 1.CP002293 – CP002294, GCA_000166115 – 1.CP002297 – CP002298, GCA_000166135 – 1.CP002299, GCA_000166275 – 1.AAXU02000001, GCA_000166295 – 1.CP001978 – CP001980, GCA_000166315 – 1.CP002286, GCA_000166335 – 1.CP002216, GCA_000166355 – 1.CP002219, GCA_000166395 – 1.CP002305, GCA_000166415 – 1.CP002304, GCA_000166455 – 2.CP003069 – CP003070, GCA_000166695 – 1.CP002326 – CP002327, GCA_000166775 – 1.CP002330, GCA_000166935 – 1.AABW01000001, GCA_000168315 – 3.CP003057, GCA_000168355 – 3.CP002007, GCA_000168575 – 2.CP002003, GCA_000168595 – 2.CP002004, GCA_000168635 – 2.CP002001, GCA_000168695 – 2.CP002002, GCA_000168775 – 2.CP003862, GCA_000169195 – 2.CP003056, GCA_000169215 – 2.CP002457, GCA_000171795 – 2.CP005388, GCA_000172575 – 2.CP002621, GCA_000172635 – 2.CP003841, GCA_000174395 – 2.CP003583 – CP003586, GCA_000174795 – 2.CP002442 – CP002443, GCA_000175095 – 2.CP002552 – CP002554, GCA_000175115 – 2.CP002479, GCA_000175215 – 2.CP002395 – CP002398, GCA_000175255 – 2.CP002850 – CP002856, GCA_000175295 – 2.CP002466, GCA_000175575 – 2.CP005986 – CP005989, GCA_000175935 – 2.CP002433 – CP002438, GCA_000176035 – 2.CP002279, GCA_000176115 – 2.CP002475 – CP002477, GCA_000176835 – 2.CP006250 – CP006251, GCA_000176855 – 2.CP002521, GCA_000176915 – 2.CP002431, GCA_000176935 – 2.CP002519 – CP002520, GCA_000177195 – 2.CP002993, GCA_000177235 – 2.CP002394, GCA_000177255 – 2.CP002418, GCA_000177535 – 2.CP002857, GCA_000177615 – 2.CP002801 – CP002803, GCA_000177635 – 2.CP002432, GCA_000177655 – 2.CP002399, GCA_000178115 – 2.CP002400, GCA_000178395 – 2.CP002835 – CP002837, GCA_000178835 – 2.CP002582, GCA_000178875 – 2.CP002383 – CP002384, GCA_000178935 – 2.CP002096, GCA_000178955 – 2.CP003130, GCA_000178975 – 2.CP002480 – CP002485, GCA_000179015 – 2.CP002449 – CP002451, GCA_000179035 – 2.CP002525, GCA_000179395 – 2.CP002917, GCA_000179635 – 2.CP002403 – CP002407, GCA_000179915 – 2.CP002467, GCA_000180175 – 2.AP012551 – AP012553, GCA_000182745 – 1.AP012159 – AP012166, GCA_000182835 – 1.CP002341 – CP002342, GCA_000182875 – 1.CP002340, GCA_000183115 – 1.CP002252, GCA_000183135 – 1.CP002345, GCA_000183155 – 1.CP002346, GCA_000183185 – 1.CP002274, GCA_000183225 – 1.CP002300, GCA_000183245 – 1.CP002301, GCA_000183285 – 1.CP002302, GCA_000183305 – 1.CP002303, GCA_000183345 – 1.CP001855 – CP001856, GCA_000183365 – 1.CP002108, GCA_000183385 – 1.CP002188, GCA_000183405 – 1.CP002347 – CP002348, GCA_000183425 – 1.CP002349 – CP002350, GCA_000183645 – 1.CP002290, GCA_000183665 – 1.CP002371, GCA_000183725 – 1.CP002355 – CP002359, GCA_000183745 – 1.CP002361 – CP002362, GCA_000184065 – 1.CP001796 – CP001797, GCA_000184085 – 1.CP002029 – CP002030, GCA_000184325 – 1.CP002377 – CP002378, GCA_000184345 – 2.CP002903, GCA_000184435 – 1.CP002385 – CP002387, GCA_000184685 – 1.CP002343, GCA_000184705 – 1.CP002344, GCA_000184745 – 1.CP002417, GCA_000184925 – 1.CP002416, GCA_000185185 – 1.CP002331, GCA_000185205 – 1.CP002332 – CP002333, GCA_000185225 – 1.CP002334 – CP002335, GCA_000185245 – 1.CP002336 – CP002337, GCA_000185745 – 1.CP002456, GCA_000185805 – 1.CP002444 – CP002445, GCA_000185885 – 1.CP002439, GCA_000185905 – 1.CP002447 – CP002448, GCA_000185965 – 1.CP002446, GCA_000185985 – 2.CP002189, GCA_000186005 – 1.CP002458, GCA_000186225 – 1.CP002352, GCA_000186245 – 1.CP002452, GCA_000186265 – 1.CP002453, GCA_000186345 – 1.CP002353 – CP002354, GCA_000186385 – 1.CP002454, GCA_000186405 – 1.CP002465, GCA_000186585 – 1.CP002469 – CP002470, GCA_000186665 – 4.CP003967 – CP003968, GCA_000186725 – 1.CP001608 – CP001611, GCA_000186745 – 1.CP002468, GCA_000186885 – 1.CP002364, GCA_000186985 – 3.CP002808, GCA_000187005 – 1.CP001962 – CP001963, GCA_000187705 – 1.CP002505 – CP002507, GCA_000187935 – 2.AEUT02000001, GCA_000188215 – 1.CP001921 – CP001923, GCA_000188715 – 1.CP002215, GCA_000188735 – 1.CP002487 – CP002490, GCA_000188955 – 5.CP007530, GCA_000189295 – 2.CP003220, GCA_000189415 – 1.CP002455, GCA_000189495 – 1.CP002478, GCA_000189515 – 1.CP002429 – CP002430, GCA_000189535 – 1.CP002379 – CP002381, GCA_000189735 – 2.CP002522 – CP002524, GCA_000189775 – 3.CP002739, GCA_000190435 – 1.CP002541, GCA_000190515 – 1.CP002508 – CP002510, GCA_000190535 – 1.CP002544, GCA_000190555 – 1.CP002536 – CP002540, GCA_000190575 – 1.CP002530 – CP002533, GCA_000190595 – 1.CP002534, GCA_000190635 – 1.CP002547, GCA_000191045 – 1.CP002543, GCA_000191145 – 1.CP002177, GCA_000191165 – 1.CP000156, GCA_000191545 – 1.CP002559 – CP002561, GCA_000191565 – 1.CP002562, GCA_000191905 – 1.CP002118 – CP002119, GCA_000191925 – 1.CP002549 – CP002550, GCA_000192105 – 1.CP002246 – CP002247, GCA_000192315 – 1.CP002571, GCA_000192335 – 1.CP002572, GCA_000192635 – 1.CP002248 – CP002250, GCA_000192705 – 1.CP002365 – CP002370, GCA_000192725 – 1.CP002459 – CP002460, GCA_000192745 – 1.CP002568 – CP002569, GCA_000192845 – 1.CP002584, GCA_000192865 – 1.CP002583, GCA_000192885 – 1.CP001851 – CP001852, GCA_000193185 – 1.CP012090, GCA_000193205 – 1.CP002512, GCA_000193355 – 1.CP001907 – CP001917, GCA_000193395 – 1.CP002589, GCA_000193435 –

3.CP003001, GCA_000193595 − 3.CP009751, GCA_000194075 − 3.CP003494, GCA_000194115 − 1.CP002609 − CP002611, GCA_000194135 − 1.CP002606, GCA_000194605 − 1.CP002542, GCA_000194745 − 1.CP002599 − CP002604, GCA_000194765 − 1.CP002618 − CP002619, GCA_000194785 − 1.CP002616 − CP002617, GCA_000194805 − 1.CP002585, GCA_000195065 − 1.CP002555 − CP002556, GCA_000195085 − 1.CP002511, GCA_000195275 − 1.CP002631 − CP002632, GCA_000195295 − 1.CP002629, GCA_000195315 − 1.CP002628, GCA_000195335 − 1.CP002630, GCA_000195395 − 3.CP006046, GCA_000195435 − 3.CP006047, GCA_000195515 − 1.CP002627, GCA_000195535 − 1.CP002557, GCA_000195555 − 1.CP002558, GCA_000195575 − 1.CP002563 − CP002564, GCA_000195755 − 1.AE017285 − AE017286, GCA_000196095 − 1.BA000031 − BA000032, GCA_000196215 − 1.CP000013 − CP000015, GCA_000196235 − 1.CP000454 − CP000457, GCA_000196315 − 1.CP002046, GCA_000196355 − 1.CP000103 − CP000106, GCA_000196455 − 1.FP565809, GCA_000196515 − 1.CP002059 − CP002061, GCA_000196535 − 1.CP001992, GCA_000196555 − 1.AP010888, GCA_000196575 − 1.AP010890 − AP010892, GCA_000196595 − 1.CP001993, GCA_000196735 − 1.FN597644, GCA_000196795 − 1.CP002080, GCA_000196815 − 1.FP929003, GCA_000196835 − 1.CP002000, GCA_000196855 − 1.FN822744, GCA_000198515 − 1.FP929140, GCA_000198775 − 1.FR687359 − FR687361, GCA_000199675 − 1.AP012029, GCA_000200735 − 1.FR773153, GCA_000202635 − 1.AP012052, GCA_000202835 − 1.AP012035 − AP012043, GCA_000203195 − 1.FR824043 − FR824044, GCA_000203215 − 1.FQ790233, GCA_000203875 − 1.CP000090 − CP000093, GCA_000203895 − 1.CP000489 − CP000491, GCA_000203915 − 1.CP000440 − CP000443, GCA_000203935 − 1.CP000469 − CP000470, GCA_000203955 − 1.CP000458 − CP000461, GCA_000204075 − 1.CP000117 − CP000121, GCA_000204115 − 1.CP002607, GCA_000204135 − 1.CP002608, GCA_000204155 − 1.CP002638 − CP002639, GCA_000204275 − 1.CP002634 − CP002635, GCA_000204295 − 1.CP002620, GCA_000204565 − 1.CP002410 − CP002415, GCA_000204625 − 1.CP002633, GCA_000204645 − 1.CP002657 − CP002658, GCA_000204665 − 1.CP002643, GCA_000204985 − 1.CP002464, GCA_000208385 − 1.CP002659, GCA_000208405 − 1.CP002637, GCA_000209655 − 1.CP002663 − CP002664, GCA_000209675 − 1.CP002667 − CP002668, GCA_000209755 − 1.FM211688, GCA_000209795 − 2.AP011541 − AP011542, GCA_000210155 − 1.CP002031, GCA_000210315 − 1.CP001996, GCA_000210515 − 1.CP002033, GCA_000211255 − 1.CP002491 − CP002495, GCA_000211295 − 1.CP002669, GCA_000211375 − 1.CP002652 − CP002655, GCA_000211545 − 5.CP006916, GCA_000211855 − 3.CP002825, GCA_000212355 − 1.CP002528, GCA_000212375 − 1.CP002689, GCA_000212395 − 1.CP002690, GCA_000212415 − 1.CP002696, GCA_000212605 − 1.AFER01000001 − AFER01000004, GCA_000212675 − 2.CP003221, GCA_000212695 − 1.CP002666, GCA_000212735 − 1.CP002691 − CP002694, GCA_000212975 − 1.CP002695, GCA_000213135 − 1.CP002605, GCA_000213155 − 1.CP002409, GCA_000213235 − 1.CP002728, GCA_000213255 − 1.CP002360, GCA_000213635 − 1.CP002614 − CP002615, GCA_000213655 − 1.CP002339, GCA_000213785 − 1.CP002726, GCA_000213805 − 1.CP002727, GCA_000213825 − 1.CP002471, GCA_000213865 − 1.CP002743, GCA_000213955 − 1.CP002725, GCA_000214095 − 3.CP002985, GCA_000214155 − 1.CP002329, GCA_000214175 − 1.CP002786 − CP002788, GCA_000214195 − 1.CP002774, GCA_000214215 − 1.CP002771, GCA_000214235 − 1.CP002773, GCA_000214355 − 1.CP001841, GCA_000214375 − 1.CP001843, GCA_000214395 − 1.CP002735, GCA_000214435 − 1.CP002736, GCA_000214665 − 1.CP002738, GCA_000214705 − 1.CP002770, GCA_000214765 − 2.CP002797, GCA_000214785 − 1.CP002764 − CP002766, GCA_000214805 − 1.CP002775, GCA_000214825 − 1.CP002776, GCA_000214845 − 1.CP002777 − CP002778, GCA_000215065 − 1.CP002809, GCA_000215085 − 1.CP002780, GCA_000215105 − 1.CP002810, GCA_000215325 − 1.CP002819 − CP002820, GCA_000215345 − 3.CP006615, GCA_000215645 − 1.CP002790, GCA_000215665 − 1.CP002791, GCA_000215705 − 1.CP000245, GCA_000215745 − 1.CP002824, GCA_000215895 − 1.CP002811 − CP002814, GCA_000215975 − 1.CP002829, GCA_000217615 − 1.CP002815, GCA_000217655 − 1.CP002103, GCA_000217675 − 1.CP002284 − CP002285, GCA_000217795 − 1.CP002683, GCA_000217815 − 1.CP002351, GCA_000217835 − 1.CP002472, GCA_000218265 − 1.CP001830 − CP001832, GCA_000218305 − 1.CP002816, GCA_000218545 − 1.CP002665, GCA_000218565 − 1.CP002826 − CP002828, GCA_000218585 − 1.CP002821 − CP002823, GCA_000218855 − 1.CP002660 − CP002662, GCA_000218875 − 1.CP002865 − CP002867, GCA_000218915 − 1.CP002869, GCA_000219045 − 1.CP002872, GCA_000219105 − 1.CP002830, GCA_000219175 − 1.CP002243, GCA_000219195 − 1.CP002244, GCA_000219215 − 1.CP002877 − CP002880, GCA_000219355 − 1.CP002130, GCA_000219375 − 1.CP002513, GCA_000219455 − 1.CP002794 − CP002796, GCA_000219515 − 3.CP006632 − CP006635, GCA_000219535 − 3.CP007806 − CP007808, GCA_000219585 − 1.CP002876, GCA_000219605 − 1.CP002881, GCA_000219705 − 1.CP002870, GCA_000219725 − 1.CP002868, GCA_000219765 − 1.CP002904, GCA_000219785 − 1.CP002898, GCA_000219805 − 1.CP002899 − CP002900, GCA_000219855 − 1.CP002901, GCA_000219875 − 1.CP002902, GCA_000219915 − 3.CP003555 − CP003556, GCA_000220005 − 2.AGTD01000001 − AGTD01000006, GCA_000220105 − 1.CP002024, GCA_000220135 − 1.CP000303, GCA_000220485 − 1.CP002910 − CP002911, GCA_000220625 − 1.CP002113, GCA_000220885 − 1.CP002915, GCA_000220945 − 1.CP002896, GCA_000220965 − 1.CP002918, GCA_000221005 − 1.CP002078 − CP002079, GCA_000221025 − 1.CP002573 − CP002577, GCA_000221045 − 1.CP002745, GCA_000221205 − 1.CP002912, GCA_000221625 − 1.CP002924, GCA_000221645 − 1.CP002927, GCA_000221965 − 1.CP002789, GCA_000221985 − 1.CP002925 − CP002926, GCA_000222305 − 1.CP002746 − CP002762, GCA_000222485 − 1.CP002955, GCA_000222835 − 1.CP002933 − CP002950, GCA_000223195 − 1.CP002997 − CP002998, GCA_000223375 − 1.CP002018 − CP002020, GCA_000223885 − 1.CP002986, GCA_000224005 − 3.CP007031, GCA_000224105 − 1.CP003021, GCA_000224435 − 1.CP002992, GCA_000224535 − 1.CP002980 − CP002981, GCA_000224555 − 1.CP002982, GCA_000224575 − 1.CP002983 − CP002984, GCA_000224675 − 1.CP003026 − CP003028,

GCA_000224745-1.CP003029-CP003031, GCA_000224965-2.CP003039, GCA_000224985-1.CP003032, GCA_000225265-1.CP003017-CP003020, GCA_000225325-1.CP002461-CP002463, GCA_000225345-1.CP003040, GCA_000225445-1.CP002645-CP002647, GCA_000225465-1.CP002648-CP002650, GCA_000225915-1.CP002914, GCA_000225955-1.CP003046, GCA_000226155-1.CP002496, GCA_000226275-1.CP001937-CP001938, GCA_000226295-1.CP002514-CP002515, GCA_000226315-1.CP002382, GCA_000226565-1.CP003060, GCA_000226605-1.CP002401-CP002402, GCA_000226625-1.CP003059, GCA_000227175-1.CP003062, GCA_000227465-1.CP002905, GCA_000227485-1.CP002906, GCA_000227605-1.CP003061, GCA_000227625-1.CP003034-CP003038, GCA_000227645-1.CP002931-CP002932, GCA_000227665-3.CP007030, GCA_000227685-3.CP007029, GCA_000227705-3.CP003255-CP003256, GCA_000227745-3.CP003154, GCA_000230275-1.CP003058, GCA_000230295-1.CP003068, GCA_000230555-1.CP003075-CP003076, GCA_000230655-3.CP007013, GCA_000230695-3.CP003470, GCA_000230895-3.CP003169, GCA_000230975-3.CP005587, GCA_000230995-3.CP003358, GCA_000231175-1.CP001221-CP001222, GCA_000231215-1.CP003084, GCA_000231385-3.CP003629, GCA_000231405-3.CP007032, GCA_000231865-1.CP002640, GCA_000231885-1.CP002641, GCA_000231905-1.CP002644, GCA_000231925-1.CP002651, GCA_000233575-1.CP002570, GCA_000233595-1.CP003085-CP003086, GCA_000233715-3.CP003273, GCA_000233735-1.CP003082, GCA_000233755-1.CP003094, GCA_000233775-1.CP003096-CP003097, GCA_000233875-1.CP002211, GCA_000233895-1.CP002212, GCA_000233915-4.CP003093, GCA_000234745-1.CP003022, GCA_000234825-2.CP003101-CP003105, GCA_000235405-3.CP003260, GCA_000235545-1.CP003047, GCA_000235585-1.CP003107, GCA_000235605-1.CP003108, GCA_000235765-3.CP003678, GCA_000236065-1.CP003087-CP003092, GCA_000236215-4.HG938370-HG938372, GCA_000236255-1.CP003128-CP003129, GCA_000236405-1.CP003015-CP003016, GCA_000236475-1.CP003132-CP003136, GCA_000236585-1.CP003126-CP003127, GCA_000236605-1.CP003125, GCA_000236665-1.CP003153, GCA_000236685-1.CP003155, GCA_000236705-1.CP003156, GCA_000237065-1.CP003150, GCA_000237085-1.CP003065, GCA_000237125-1.CP003166, GCA_000237145-1.CP003170, GCA_000237205-1.FR872581-FR872582, GCA_000237305-1.FQ859184-FQ859185, GCA_000237325-1.HE577054-HE577055, GCA_000237845-1.CP002428, GCA_000237975-1.CP003179-CP003180, GCA_000237995-2.CP003137-CP003145, GCA_000238175-1.CP003176-CP003177, GCA_000238195-1.CP003174-CP003175, GCA_000238215-1.CP003191, GCA_000238995-1.CP003199, GCA_000239175-1.CP003192, GCA_000239195-1.CP003187-CP003189, GCA_000239235-1.CP003193-CP003194, GCA_000239975-1.CP002886-CP002887, GCA_000240015-1.CP003195, GCA_000240035-1.CP003196, GCA_000240055-1.CP003197, GCA_000240075-2.CP003222, GCA_000240325-1.CP003218, GCA_000241025-2.CP003099, GCA_000241055-1.AFVZ01000001-AFVZ01000002, GCA_000241125-1.CP003231, GCA_000241385-1.CP003241-CP003242, GCA_000241855-1.CP003152, GCA_000241875-1.CP003206, GCA_000241895-1.CP003207, GCA_000241915-1.CP003208, GCA_000241935-1.CP003209, GCA_000242255-3.CP003350, GCA_000242335-3.CP003368-CP003371, GCA_000242455-3.CP003364-CP003367, GCA_000242595-3.CP003282, GCA_000242635-3.CP003349, GCA_000242775-1.CP003214, GCA_000242895-3.CP005586, GCA_000242915-2.AFRZ01000001, GCA_000242935-3.CP007053-CP007054, GCA_000243075-1.CP003171-CP003173, GCA_000243115-3.CP007035, GCA_000243135-3.CP003344, GCA_000243155-3.CP003348, GCA_000244875-1.CP003259, GCA_000245355-1.CP003275-CP003277, GCA_000245495-1.CP003033, GCA_000245515-1.CP003109-CP003114, GCA_000245535-1.CP003278-CP003279, GCA_000246835-1.CP003295-CP003296, GCA_000246855-1.CP003178, GCA_000247605-1.CP002987, GCA_000247715-1.CP003119-CP003120, GCA_000248095-3.CP011013-CP011014, GCA_000248375-1.CP003077, GCA_000248415-1.CP003048, GCA_000248435-1.CP003049, GCA_000250635-1.CP002831-CP002832, GCA_000250655-1.CP003235, GCA_000250675-3.CP003876, GCA_000250855-1.CP003330-CP003331, GCA_000250905-1.CP003116, GCA_000250925-1.CP003121, GCA_000250945-1.CP003351-CP003354, GCA_000251085-2.CP003357, GCA_000252365-1.CP003375, GCA_000252445-1.CP002191-CP002197, GCA_000252855-1.CP003388, GCA_000252975-1.FR687253, GCA_000253035-1.FR872580, GCA_000253135-1.FR714927-FR714929, GCA_000253155-1.FR720602, GCA_000253255-1.FR856859-FR856862, GCA_000253275-1.FQ859182-FQ859183, GCA_000253295-1.FQ859181, GCA_000253315-1.FR873482, GCA_000253335-1.FR873481, GCA_000253395-1.FR875178, GCA_000255115-3.CP003639-CP003641, GCA_000255135-1.CP003257-CP003258, GCA_000255155-1.CP003213, GCA_000255175-1.CP003210, GCA_000255195-1.CP003215, GCA_000255215-1.CP003211, GCA_000255235-1.CP003212, GCA_000255255-1.CP003217, GCA_000255275-1.CP003216, GCA_000255295-1.CP003389, GCA_000255535-1.CP003403-CP003406, GCA_000255915-1.CP003313-CP003314, GCA_000255935-1.CP003385, GCA_000255955-1.CP002953-CP002954, GCA_000257275-1.CP002291, GCA_000257545-3.CP005960-CP005961, GCA_000258025-1.CP002970-CP002971, GCA_000258145-1.CP002967-CP002969, GCA_000258245-1.CP003252-CP003254, GCA_000258385-1.CP003407, GCA_000258405-1.CP003418, GCA_000258535-2.CP003422, GCA_000258885-1.CP002091-CP002093, GCA_000258905-1.CP003414, GCA_000259155-2.CP003421, GCA_000259175-1.CP003488, GCA_000259235-1.CP003486-CP003487, GCA_000259255-1.CP003479-CP003480, GCA_000259275-1.CP003481-CP003482, GCA_000259365-1.CP003492, GCA_000259545-1.CP001409, GCA_000260515-1.CP002833-CP002834, GCA_000260715-1.CP002567, GCA_000260925-1.CP003415, GCA_000260965-1.CP003380-CP003381, GCA_000260985-2.CP003390, GCA_000261025-1.CP003502-CP003503, GCA_000262125-1.CP001925-CP001927, GCA_000262145-

1.CP003122, GCA_000262165 − 1.CP003491, GCA_000262205 − 1.CP003402, GCA_000262305 − 1.CP001560, GCA_000262325 − 2.CP003041 − CP003042, GCA_000262385 − 1.CP003332, GCA_000262655 − 1.CP003419 − CP003420, GCA_000262675 − 1.CP003499, GCA_000262695 − 1.CP003514 − CP003517, GCA_000263195 − 1.CP002961 − CP002966, GCA_000263215 − 1.CP003312, GCA_000263755 − 2.CP003540, GCA_000264455 − 2.CP003236 − CP003240, GCA_000264665 − 1.CP003588 − CP003589, GCA_000264765 − 2.CP006664 − CP006666, GCA_000265095 − 1.CP001361, GCA_000265295 − 1.CP003333, GCA_000265365 − 1.CP003274, GCA_000265385 − 1.CP003280, GCA_000265405 − 1.CP003281, GCA_000265425 − 1.CP003379, GCA_000265465 − 1.CP003283, GCA_000265505 − 1.CP003345, GCA_000265545 − 1.CP003652, GCA_000266885 − 1.CP002959 − CP002960, GCA_000266905 − 1.CP003053 − CP003055, GCA_000266925 − 1.CP003198, GCA_000266945 − 1.CP003360 − CP003361, GCA_000267545 − 1.CP003677, GCA_000269925 − 1.AP009332, GCA_000269945 − 1.AP009333, GCA_000269985 − 1.AP010968, GCA_000270005 − 1.AP011940, GCA_000270025 − 1.AP011941 − AP011942, GCA_000270045 − 1.AP011943 − AP011944, GCA_000270065 − 1.AP011945, GCA_000270105 − 1.AP012030, GCA_000270125 − 1.AP012032 − AP012033, GCA_000270145 − 1.AP012053, GCA_000270165 − 1.AP012054, GCA_000270225 − 1.AP012203, GCA_000270245 − 1.AP012204, GCA_000270265 − 1.AP012205, GCA_000270285 − 1.AP012211, GCA_000270305 − 1.AP012212, GCA_000270345 − 1.CP001641, GCA_000270365 − 1.CP001642, GCA_000270385 − 1.CP002804, GCA_000270405 − 1.CP002805, GCA_000270425 − 1.CP002806, GCA_000270445 − 1.CP002807, GCA_000270525 − 1.CP002058, GCA_000271325 − 1.AP012157 − AP012158, GCA_000271365 − 1.CP003149, GCA_000271665 − 2.CP010978, GCA_000271865 − 1.CP003686, GCA_000272715 − 3.CP010283, GCA_000272735 − 3.CP010281, GCA_000272755 − 3.CP010282, GCA_000272775 − 3.CP010279, GCA_000272795 − 2.CP009565, GCA_000272815 − 2.CP009561, GCA_000272835 − 3.CP010284, GCA_000272895 − 3.CP010280, GCA_000275625 − 1.AFRY01000001, GCA_000276685 − 1.CP003264, GCA_000276825 − 1.CP003324, GCA_000277085 − 1.CP003233, GCA_000277105 − 1.CP003234, GCA_000277125 − 1.CP003322, GCA_000277145 − 1.CP003323, GCA_000277165 − 1.CP003391, GCA_000277185 − 1.CP003392, GCA_000277205 − 1.CP003393, GCA_000277245 − 1.CP003395, GCA_000277265 − 1.CP003396, GCA_000277285 − 1.CP003397, GCA_000277305 − 1.CP003398, GCA_000277325 − 1.CP003497, GCA_000277345 − 1.CP003498, GCA_000277365 − 1.CP003472, GCA_000277385 − 1.CP003473, GCA_000277405 − 1.CP003474, GCA_000277425 − 1.CP003475, GCA_000277715 − 1.CP002734, GCA_000277735 − 2.CP003248, GCA_000277755 − 1.CP003704 − CP003707, GCA_000277775 − 2.CP003699, GCA_000277795 − 1.CP003703, GCA_000279145 − 1.CP003557, GCA_000279165 − 1.CP003725, GCA_000280315 − 2.CP007598 − CP007599, GCA_000280925 − 3.CP012109, GCA_000281175 − 1.AP012337, GCA_000281195 − 1.CP003726 − CP003728, GCA_000281215 − 1.CP003734, GCA_000281235 − 1.CP003731, GCA_000281435 − 2.CP007727 − CP007730, GCA_000281535 − 2.CP008827 − CP008830, GCA_000282715 − 1.CP003729, GCA_000283275 − 1.CP003735, GCA_000283295 − 1.CP001663, GCA_000283515 − 1.AP012222 − AP012223, GCA_000283535 − 1.AP012224, GCA_000283555 − 1.AP012210, GCA_000283575 − 1.AP012044 − AP012045, GCA_000283595 − 1.AP011533, GCA_000283615 − 1.AP012046, GCA_000283675 − 1.AP007209 − AP007214, GCA_000283695 − 1.HE617159, GCA_000283735 − 1.AP011957 − AP011959, GCA_000283755 − 1.AP012303, GCA_000283775 − 1.CP003306, GCA_000283795 − 1.CP003307, GCA_000283815 − 1.CP003309, GCA_000283875 − 1.HE617160 − HE617161, GCA_000283915 − 1.CP003304, GCA_000283935 − 1.CP003311, GCA_000283955 − 1.CP003305, GCA_000283995 − 1.CP003308, GCA_000284015 − 1.FO117623, GCA_000284035 − 1.FO082843, GCA_000284055 − 1.CP003334 − CP003337, GCA_000284075 − 1.CP003342 − CP003343, GCA_000284095 − 1.AP012292 − AP012301, GCA_000284115 − 1.AP012338 − AP012339, GCA_000284135 − 1.AP012276, GCA_000284155 − 1.CP003338 − CP003339, GCA_000284175 − 1.CP003340, GCA_000284195 − 1.CP003341, GCA_000284215 − 1.AP012277, GCA_000284235 − 1.AP012048 − AP012049, GCA_000284255 − 1.AP012320, GCA_000284275 − 1.AP012279, GCA_000284295 − 1.AP012319, GCA_000284315 − 1.AP012342, GCA_000284335 − 1.AP012051, GCA_000284355 − 1.AP012047, GCA_000284375 − 1.AP012206, GCA_000284395 − 1.HE774679, GCA_000284415 − 2.HE663493, GCA_000284435 − 1.AP012209, GCA_000284455 − 1.AP012278, GCA_000284495 − 1.CU651637, GCA_000284555 − 1.AP012280, GCA_000284575 − 1.AP012336, GCA_000284615 − 1.FO203363, GCA_000284635 − 1.AP012344 − AP012345, GCA_000285655 − 3.HG941718 − HG941720, GCA_000286275 − 1.CP003737, GCA_000286435 − 2.CP004345, GCA_000286675 − 1.CP003506, GCA_000286695 − 1.CP003507, GCA_000286715 − 1.CP003508, GCA_000286735 − 1.CP003509, GCA_000286755 − 1.CP003510, GCA_000286775 − 1.CP003511, GCA_000286795 − 1.CP003512, GCA_000286815 − 1.CP003513, GCA_000287215 − 3.CP007567 − CP007568, GCA_000287235 − 1.CP003541, GCA_000287255 − 1.CP003542, GCA_000287275 − 1.CP003543, GCA_000287295 − 1.CP003544, GCA_000287315 − 1.CP003545, GCA_000287335 − 1.CP003546, GCA_000287355 − 1.CP003547, GCA_000292405 − 1.CP003770, GCA_000292415 − 1.CP003747 − CP003751, GCA_000292445 − 1.CP003771, GCA_000292455 − 1.CP003752 − CP003760, GCA_000292485 − 1.CP003772, GCA_000292505 − 1.CP003773, GCA_000292685 − 1.CP003708, GCA_000292705 − 1.CP003763 − CP003769, GCA_000292915 − 1.CP003774 − CP003775, GCA_000293765 − 1.CP003783, GCA_000293885 − 3.CP010945, GCA_000294365 − 1.CP003785, GCA_000294495 − 1.CP003736, GCA_000294535 − 1.CP003776, GCA_000294635 − 1.CP003781 − CP003782, GCA_000294695 − 2.CP003203 − CP003205, GCA_000294715 − 1.CP003784, GCA_000295655 − 1.CP003787, GCA_000296575 − 1.CP003490, GCA_000296595 − 1.CP003808, GCA_000297055 − 2.AP013066 − AP013067, GCA_000297075 − 2.HG916826, GCA_000298385 − 1.CP003835, GCA_000298415 − 1.CP003791, GCA_000298875 − 1.CP003839, GCA_000299015 − 1.CP001845, GCA_000299095 − 1.CP003809, GCA_000299115 − 1.CP003801, GCA_000299135 − 1.CP003810, GCA_000299235 − 1.CP002919, GCA_000299255 − 1.CP003297 − CP003300, GCA_000299335 − 2.CP004387, GCA_000299355 − 1.CP003326 −

CP003327, GCA_000299435-1.CP003063, GCA_000299455-1.CP003289-CP003292, GCA_000299475-1.CP003301-CP003303, GCA_000299955-1.CP003844, GCA_000299965-1.CP003230, GCA_000299995-1.CP003845, GCA_000300005-1.CP003466, GCA_000300035-1.CP003867, GCA_000300045-1.CP003866, GCA_000300075-1.CP003868, GCA_000300095-1.CP003863-CP003865, GCA_000300175-1.CP003873-CP003874, GCA_000300235-2.CP004388-CP004389, GCA_000300295-2.CP011849-CP011852, GCA_000300455-3.CP007215, GCA_000300475-1.CP003687-CP003694, GCA_000302475-2.CP003919, GCA_000302515-1.CP003877, GCA_000302535-1.CP003872, GCA_000302575-1.CP003856, GCA_000304215-1.CP003325, GCA_000304315-1.HE956757, GCA_000304415-1.HE995405-HE995408, GCA_000304455-1.HE983995-HE983996, GCA_000304735-1.CP003882, GCA_000305335-1.CP002888-CP002889, GCA_000305775-1.CP003869, GCA_000305785-2.CP003746, GCA_000305815-1.CP003870, GCA_000305935-1.CP003732, GCA_000306745-1.CP003889-CP003898, GCA_000306785-1.FO203431, GCA_000306805-1.AP010969, GCA_000306845-1.FQ958211, GCA_000306885-1.HE660029, GCA_000306905-1.FR733645, GCA_000306985-1.FR733650, GCA_000307005-1.FR733649, GCA_000307025-1.FR733643, GCA_000307045-1.FR733647, GCA_000307065-1.FR733651, GCA_000307085-1.FR733642, GCA_000307105-1.FO203503, GCA_000307165-1.AP012050, GCA_000307535-1.CP003901, GCA_000307615-1.FR733644, GCA_000307795-1.CP003904, GCA_000307815-1.CP003905, GCA_000307835-1.CP003906, GCA_000307975-2.CP007577-CP007580, GCA_000309885-1.CP003249-CP003251, GCA_000310065-1.CP003493, GCA_000310085-1.CP003917-CP003918, GCA_000310105-1.AMYA01000001-AMYA01000146, GCA_000311765-1.CP003926-CP003927, GCA_000312685-1.CP003157-CP003165, GCA_000312705-1.CP003284-CP003288, GCA_000313175-2.CP006694-CP006695, GCA_000313385-1.CP003932, GCA_000313635-1.CP003914, GCA_000315955-1.CP003483-CP003485, GCA_000316175-1.CP003880, GCA_000316515-1.CP003495, GCA_000316575-1.CP003943, GCA_000316605-1.CP003946, GCA_000316625-1.CP003548, GCA_000316645-1.CP003552-CP003554, GCA_000316665-1.CP003549-CP003551, GCA_000316685-1.CP003558-CP003559, GCA_000317025-1.CP003590, GCA_000317045-1.CP003591, GCA_000317065-1.CP003592-CP003593, GCA_000317085-1.CP003594-CP003596, GCA_000317105-1.CP003607-CP003609, GCA_000317125-1.CP003597-CP003599, GCA_000317435-1.CP003610-CP003613, GCA_000317475-1.CP003614-CP003619, GCA_000317495-1.CP003620-CP003628, GCA_000317515-1.CP003630-CP003638, GCA_000317555-1.CP003646-CP003650, GCA_000317575-1.CP003653-CP003658, GCA_000317615-1.CP003944, GCA_000317635-1.CP003945, GCA_000317655-1.CP003940, GCA_000317675-1.CP003947-CP003948, GCA_000317695-1.CP003659-CP003665, GCA_000317835-1.CP003382-CP003384, GCA_000317855-1.HE858529, GCA_000317875-1.CP003476-CP003478, GCA_000317895-1.CP002930, GCA_000317915-1.HF558530, GCA_000317975-2.HE999757, GCA_000318035-1.HE970764-HE970765, GCA_000318055-1.HF558398, GCA_000318825-1.HE601950, GCA_000318885-1.HE601954, GCA_000319225-1.CP003978, GCA_000319245-1.CP003733, GCA_000319385-1.CP003539, GCA_000319475-1.CP003838, GCA_000319575-2.CP005963, GCA_000321395-1.CP003695, GCA_000321415-1.CP003989, GCA_000325665-1.CP002873, GCA_000325705-1.CP003346, GCA_000325725-1.CP003738-CP003739, GCA_000325745-1.CP003789, GCA_000327045-1.CP003051-CP003052, GCA_000327065-1.CP003071-CP003074, GCA_000328405-1.CP003972-CP003973, GCA_000328545-1.CP003066-CP003067, GCA_000328565-1.CP003078-CP003081, GCA_000328625-1.CP003359, GCA_000328705-1.HE804045, GCA_000328725-1.HF559394, GCA_000329365-2.CP007528-CP007529, GCA_000330485-2.CP012347-CP012348, GCA_000330845-1.CP003938, GCA_000330865-1.CP003942, GCA_000330885-1.CP004015-CP004018, GCA_000331065-1.CP003903, GCA_000331085-2.CP003913, GCA_000331445-1.HE663067, GCA_000331695-1.CP004020, GCA_000331715-1.CP004029-CP004032, GCA_000331735-1.CP004025, GCA_000331975-1.CP003263, GCA_000331995-1.CP004044, GCA_000332115-1.CP001397, GCA_000332735-1.CP003668-CP003676, GCA_000334405-1.CP003605-CP003606, GCA_000334515-1.FO203353-FO203355, GCA_000335875-2.CP011394-CP011395, GCA_000336405-1.CP004063-CP004064, GCA_000336425-1.CP003959-CP003960, GCA_000336445-1.CP004008, GCA_000336465-1.CP004045, GCA_000338095-1.CP004085, GCA_000338695-1.CP002744, GCA_000338715-2.CP003900, GCA_000338735-1.CP004019, GCA_000338755-1.CP004069-CP004076, GCA_000339015-1.CP004091-CP004094, GCA_000340785-1.CP003265-CP003272, GCA_000340795-1.CP003803, GCA_000340825-1.CP003804, GCA_000340845-1.CP003720-CP003722, GCA_000340865-1.CP003805, GCA_000340885-1.CP004121-CP004122, GCA_000340905-1.CP003806, GCA_000340925-1.CP003807, GCA_000341345-1.CP003697-CP003698, GCA_000341355-1.CP003123, GCA_000341385-1.CP003124, GCA_000341395-1.CP003985-CP003986, GCA_000341655-1.CP004079, GCA_000341665-1.CP004123-CP004137, GCA_000341695-1.CP004080, GCA_000341855-1.CP004347, GCA_000342265-1.CP004348, GCA_000344575-1.AP012281, GCA_000344745-1.CP003329, GCA_000344765-1.CP003922, GCA_000344785-1.CP004354-CP004356, GCA_000344805-1.AP012603, GCA_000346065-1.CP004138-CP004140, GCA_000346595-1.CP004005, GCA_000347595-1.CP003745, GCA_000347615-1.CP003885-CP003886, GCA_000347635-1.CP003837, GCA_000347675-2.CP004036-CP004041, GCA_000347695-1.CP004346, GCA_000348565-1.CP004141, GCA_000348585-1.CP004399-CP004402, GCA_000348705-1.ALJW01000001-ALJW01000002, GCA_000348725-1.CP003537, GCA_000348745-1.CP003538, GCA_000348765-2.CP004390, GCA_000348785-1.AP012057, GCA_000348865-1.AP012600, GCA_000348885-1.AP012601-AP012602, GCA_000349225-1.CP003778-CP003780, GCA_000349745-1.CP004358, GCA_000349765-1.CP004752, GCA_000349785-1.CP004753, GCA_000349795-1.CP004405,

GCA_000349845 − 1.CP004143, GCA_000349945 − 1.AP012304 − AP012305, GCA_000349975 − 1.AP012492, GCA_000350185 − 1.AP012306, GCA_000350205 − 1.AP012340, GCA_000354175 − 2.CP006718 − CP006719, GCA_000359505 − 1.CP004061, GCA_000359525 − 1.CP004370, GCA_000359625 − 1.AP012167 − AP012176, GCA_000363905 − 1.CP004888, GCA_000364725 − 1.CP003881, GCA_000364765 − 1.CP003963 − CP003964, GCA_000364785 − 1.CP003965 − CP003966, GCA_000364805 − 1.CP003990 − CP003991, GCA_000364825 − 1.CP005082, GCA_000367205 − 1.CP005287, GCA_000367405 − 1.CP004889, GCA_000367425 − 1.CP004142, GCA_000376585 − 1.CP003883, GCA_000376605 − 1.CP003884, GCA_000376625 − 1.CP004357, GCA_000376645 − 1.CP005383, GCA_000376705 − 1.CP003293 − CP003294, GCA_000380335 − 1.CP005094, GCA_000380365 − 1.CP005095, GCA_000382905 − 1.HE802067, GCA_000382925 − 1.HE999704, GCA_000382945 − 1.HE999705, GCA_000382965 − 1.HE579065 − HE579066, GCA_000382985 − 1.HE579067 − HE579068, GCA_000383005 − 1.HE579073 − HE579074, GCA_000385525 − 1.CP004095 − CP004096, GCA_000385905 − 1.CP002099, GCA_000385925 − 1.CP004409, GCA_000385945 − 1.CP005080, GCA_000389635 − 1.CP003261 − CP003262, GCA_000389675 − 2.CP005926, GCA_000389905 − 1.CP004886, GCA_000389925 − 1.CP005386, GCA_000389945 − 1.CP005387, GCA_000389965 − 1.CP005929, GCA_000390085 − 1.CP005928, GCA_000390245 − 1.CP003993, GCA_000390265 − 1.CP004077, GCA_000392435 − 1.CP005957, GCA_000392455 − 3.CP005490, GCA_000392475 − 3.CP005491, GCA_000392515 − 3.CP005492, GCA_000392535 − 3.CP005493, GCA_000397165 − 1.CP005958, GCA_000397185 − 1.CP002548, GCA_000397205 − 1.CP003190, GCA_000400615 − 1.CP002884, GCA_000400635 − 2.CP003410 − CP003411, GCA_000400855 − 1.CP003131, GCA_000400875 − 1.CP003723 − CP003724, GCA_000400935 − 1.CP005077, GCA_000400955 − 1.CP005078, GCA_000401175 − 1.CP005964, GCA_000401555 − 1.CP005966, GCA_000402035 − 1.AP013058 − AP013062, GCA_000403625 − 1.CP005941, GCA_000404145 − 1.CP004047, GCA_000404185 − 1.CP004048, GCA_000404205 − 1.CP005079, GCA_000404245 − 1.CP003730, GCA_000406765 − 2.CP011980 − CP011984, GCA_000408885 − 1.CP005965, GCA_000410515 − 1.CP005991 − CP005992, GCA_000410575 − 1.CP005976, GCA_000410995 − 1.CP006011 − CP006017, GCA_000412205 − 1.CP006033 − CP006043, GCA_000412265 − 2.CP011664 − CP011667, GCA_000412675 − 1.AP013070, GCA_000412695 − 1.AP013068 − AP013069, GCA_000412755 − 1.CP003982 − CP003983, GCA_000412775 − 1.CP006044 − CP006045, GCA_000414035 − 1.CP006245, GCA_000414215 − 1.CP004053, GCA_000416365 − 2.CP009914, GCA_000417085 − 2.CP011989 − CP011993, GCA_000417225 − 2.CP011976 − CP011979, GCA_000417265 − 2.CP011985 − CP011988, GCA_000418305 − 1.CP006265 − CP006268, GCA_000418325 − 1.CP003969, GCA_000418345 − 1.CP005288 − CP005289, GCA_000418365 − 1.CP003696, GCA_000418475 − 1.CP005484, GCA_000418495 − 1.CP005485, GCA_000418515 − 1.CP005486 − CP005487, GCA_000419405 − 1.CP003847 − CP003848, GCA_000422045 − 1.CP006366, GCA_000422085 − 1.CP006252 − CP006253, GCA_000422095 − 1.CP006574, GCA_000422125 − 1.CP006578, GCA_000422145 − 1.CP005972, GCA_000422165 − 1.CP002081, GCA_000427035 − 1.HF952104, GCA_000427055 − 1.HF952106, GCA_000427075 − 1.HF952105, GCA_000427215 − 1.CP003802, GCA_000427275 − 1.CP006573, GCA_000430085 − 2.CP005389 − CP005391, GCA_000430125 − 1.CP006055 − CP006057, GCA_000430165 − 1.CP006602, GCA_000430385 − 1.CP006006, GCA_000430405 − 1.CP006004 − CP006005, GCA_000430425 − 1.CP006007 − CP006008, GCA_000438585 − 1.CP006594 − CP006595, GCA_000438605 − 1.CP006591, GCA_000438625 − 1.CP006592, GCA_000438645 − 1.CP006593, GCA_000438665 − 1.CP006596, GCA_000438825 − 1.CP006566, GCA_000439255 − 1.CP006608 − CP006609, GCA_000439275 − 1.CP006603, GCA_000439295 − 2.CP006610, GCA_000439395 − 1.CP005384, GCA_000439415 − 1.CP006053 − CP006054, GCA_000439435 − 1.CP005074 − CP005075, GCA_000439455 − 1.CP005076, GCA_000439475 − 1.CP004846 − CP004847, GCA_000439495 − 1.CP004848, GCA_000439535 − 1.CP004851, GCA_000439555 − 1.CP004852, GCA_000439575 − 1.CP004853 − CP004854, GCA_000439595 − 1.CP004855, GCA_000439695 − 1.CP006469 − CP006470, GCA_000439735 − 1.CP006619, GCA_000439755 − 1.CP006616, GCA_000439775 − 1.CP006617, GCA_000441535 − 1.CP003025, GCA_000441555 − 1.CP003468 − CP003469, GCA_000441575 − 1.CP003467, GCA_000441585 − 1.CP002671, GCA_000441615 − 1.CP002672, GCA_000441635 − 1.CP002673, GCA_000441655 − 1.CP002674, GCA_000441675 − 1.CP002675, GCA_000441695 − 1.CP002676, GCA_000441715 − 1.CP002677, GCA_000441735 − 1.CP002678, GCA_000441755 − 1.CP002679, GCA_000441775 − 1.CP002680, GCA_000441795 − 1.CP002681, GCA_000441815 − 1.CP002682, GCA_000442415 − 1.CP006631, GCA_000442435 − 1.CP005950 − CP005956, GCA_000442605 − 1.CP006059, GCA_000442635 − 1.CP006060, GCA_000442645 − 1.CP003924 − CP003925, GCA_000444405 − 1.CP006620 − CP006626, GCA_000444425 − 1.CP004022, GCA_000444445 − 1.CP006575, GCA_000444465 − 1.CP005925, GCA_000444875 − 1.CP006259 − CP006261, GCA_000444995 − 1.CP006650 − CP006655, GCA_000445035 − 1.CP004374 − CP004376, GCA_000445405 − 1.CP006656 − CP006658, GCA_000445995 − 2.CP006254 − CP006255, GCA_000447675 − 1.CP004120, GCA_000452705 − 3.CP007014 − CP007015, GCA_000454025 − 1.CP003777, GCA_000454045 − 1.CP003761 − CP003762, GCA_000455565 − 1.HG328253, GCA_000455585 − 1.HG328254, GCA_000455605 − 1.HE774682, GCA_000462955 − 1.CP006706, GCA_000462975 − 1.CP006699 − CP006701, GCA_000462995 − 1.CP003786, GCA_000463015 − 1.CP003911 − CP003912, GCA_000463055 − 1.CP003979 − CP003981, GCA_000463355 − 1.CP003857, GCA_000463385 − 1.CP003858, GCA_000463395 − 1.CP003800, GCA_000463425 − 1.CP003859, GCA_000463445 − 1.CP003840, GCA_000463465 − 1.CP003860, GCA_000463505 − 1.CP003861, GCA_000464435 − 1.CP006605 − CP006607, GCA_000465235 − 1.CP004066 − CP004068, GCA_000465255 − 1.CP005967, GCA_000466065 − 2.CP006707, GCA_000466075 − 2.CP006709 − CP006710, GCA_000466105 − 2.CP006708, GCA_000468515 − 1.CP006584, GCA_000468615 − 2.CP009976, GCA_000468915 − 2.CP006729, GCA_000468955 − 1.CP004884, GCA_000470765 − 1.CP004033, GCA_000470775 − 1.CP006734,

GCA_000470805 − 1.CP004034, GCA_000470825 − 1.CP004035, GCA_000470845 − 1.CP003603, GCA_000470865 − 1.CP003604,
GCA_000471925 − 1.CP006667 − CP006669, GCA_000471945 − 1.CP003941, GCA_000471965 − 1.CP005488 − CP005489,
GCA_000471985 − 1.CP006645, GCA_000473245 − 1.CP006643, GCA_000473275 − 1.CP006717, GCA_000473745 − 3.CP008739,
GCA_000473995 − 1.CP006721, GCA_000474015 − 1.CP006648, GCA_000477035 − 2.CP009887, GCA_000477415 − 1.CP006771,
GCA_000477435 − 1.CP004885, GCA_000478255 − 2.CP009054 − CP009055, GCA_000478825 − 2.CP007542 − CP007545,
GCA_000478885 − 1.AP013105, GCA_000478905 − 1.AP012554, GCA_000478925 − 1.AP013072, GCA_000479315 − 1.CP006776,
GCA_000479335 − 1.CP006805, GCA_000479355 − 1.CP006778, GCA_000484195 − 2.CP011259, GCA_000484535 − 1.CP003587,
GCA_000485885 − 1.CP006838 − CP006840, GCA_000485905 − 1.CP005990, GCA_000486365 − 2.CP011396, GCA_000486405 −
2.CP007505, GCA_000486445 − 2.CP012349, GCA_000486765 − 2.CP012346, GCA_000487295 − 2.CP012344 − CP012345,
GCA_000487575 − 2.CP007531, GCA_000487615 − 2.CP007534, GCA_000487775 − 2.CP007533, GCA_000487915 − 2.CP007532,
GCA_000493375 − 1.HG514499 − HG514500, GCA_000493495 − 1.HG428754, GCA_000493735 − 1.CP006812 − CP006817,
GCA_000493755 − 1.CP006784 − CP006789, GCA_000493775 − 1.AP012976, GCA_000494755 − 1.CP006272, GCA_000494775 −
1.CP006702 − CP006703, GCA_000494835 − 1.CP006845, GCA_000494855 − 1.CP003976 − CP003977, GCA_000494875 −
1.CP004014, GCA_000494895 − 1.CP006246, GCA_000494915 − 1.CP003961 − CP003962, GCA_000495455 − 2.CP009974 −
CP009975, GCA_000495505 − 1.CP006833, GCA_000495935 − 2.CP007547, GCA_000496265 − 1.CP006854, GCA_000496285 −
1.CP006863 − CP006865, GCA_000496595 − 1.CP006604, GCA_000496605 − 1.CP004054, GCA_000496635 − 1.CP006866,
GCA_000496645 − 1.CP004055, GCA_000496815 − 1.CP006849, GCA_000497265 − 2.AP014547, GCA_000497505 − 1.CP006736 −
CP006737, GCA_000498315 − 1.CP006888, GCA_000498335 − 1.CP006889, GCA_000498675 − 1.CP006811, GCA_000498975 −
2.CP009365, GCA_000499365 − 1.CP006730, GCA_000499665 − 2.LN794217, GCA_000499805 − 2.CP011913, GCA_000500935 −
1.CP006682, GCA_000503835 − 1.CP006914, GCA_000503845 − 1.CP006876, GCA_000503895 − 1.CP006912, GCA_000503915 −
1.CP006915, GCA_000504045 − 1.CP006853, GCA_000504125 − 1.AP013036 − AP013041, GCA_000504545 − 1.CP006731 −
CP006733, GCA_000505705 − 1.CP005994 − CP005995, GCA_000505725 − 1.CP006875, GCA_000507225 − 1.CP006945 −
CP006946, GCA_000507245 − 1.CP006939, GCA_000508205 − 1.CP006852, GCA_000508225 − 1.CP006917, GCA_000508245 −
1.CP006935, GCA_000508265 − 1.CP006952, GCA_000508765 − 1.CP006937, GCA_000510265 − 1.CP006745, GCA_000510285 −
1.CP006978, GCA_000510305 − 1.CP006931, GCA_000510325 − 1.CP006979, GCA_000511305 − 1.CP006996 − CP006998,
GCA_000511325 − 1.CP007012, GCA_000511355 − 1.CP006773 − CP006775, GCA_000511385 − 1.CP006966 − CP006973,
GCA_000511405 − 1.CP003355 − CP003356, GCA_000511895 − 1.CP004001 − CP004002, GCA_000511915 − 1.CP004003 −
CP004004, GCA_000512125 − 1.CP007025, GCA_000512165 − 1.CP003994 − CP004000, GCA_000512185 − 1.CP006579,
GCA_000512355 − 1.CP004006 − CP004007, GCA_000512375 − 1.CP006580, GCA_000512395 − 1.CP003328, GCA_000512735 −
1.CP007028, GCA_000512745 − 1.CP006999 − CP007002, GCA_000512775 − 1.CP001974 − CP001975, GCA_000512835 −
1.CP001970 − CP001972, GCA_000512895 − 1.CP007033, GCA_000512915 − 1.CP007034, GCA_000512955 − 1.CP000574,
GCA_000513295 − 1.HG794546, GCA_000513355 − 1.CBVU010000001 − CBVU010000008, GCA_000513395 − 1.CBVT010000001 −
CBVT010000008, GCA_000513415 − 1.CBVS010000001 − CBVS010000008, GCA_000513475 − 1.HG793133, GCA_000517305 −
1.CP007039, GCA_000517365 − 1.CP002082, GCA_000517565 − 1.CP007051 − CP007052, GCA_000517605 − 1.CP007045 −
CP007050, GCA_000520015 − 2.CP007044, GCA_000520035 − 1.CP006027 − CP006029, GCA_000520055 − 1.CP006262 −
CP006264, GCA_000520875 − 1.CP007067 − CP007070, GCA_000520895 − 1.CP004023 − CP004024, GCA_000521505 −
1.CP003915 − CP003916, GCA_000521525 − 1.CP002697 − CP002698, GCA_000521545 − 1.CP002699 − CP002700, GCA_000521565 −
1.CP002701 − CP002702, GCA_000521585 − 1.CP002703 − CP002704, GCA_000521605 − 1.CP006942, GCA_000521645 −
1.CP004042 − CP004043, GCA_000521655 − 1.CP006943, GCA_000521685 − 1.CP006953, GCA_000521695 − 1.CP006944,
GCA_000521725 − 1.CP006954, GCA_000521745 − 1.CP006955, GCA_000521765 − 1.CP006956, GCA_000522985 − 1.CP007127 −
CP007130, GCA_000523045 − 1.AP012496, GCA_000523235 − 1.CP006850, GCA_000524555 − 1.CP007154, GCA_000524575 −
1.CP007148, GCA_000524595 − 1.CP007147, GCA_000525635 − 1.CP007155, GCA_000525655 − 1.CP007156 − CP007157,
GCA_000525675 − 1.CP002190, GCA_000525715 − 1.CP002427, GCA_000550745 − 1.CP004081, GCA_000550765 − 1.CP004360 −
CP004365, GCA_000550785 − 1.CP004350 − CP004352, GCA_000550805 − 1.CP004353, GCA_000565155 − 1.CP007172,
GCA_000565175 − 1.CP006681, GCA_000565195 − 1.CP006720, GCA_000565215 − 1.CP006934, GCA_000567905 − 1.CP004089 −
CP004090, GCA_000567925 − 1.CP004097 − CP004098, GCA_000567945 − 1.CP004117 − CP004118, GCA_000568455 −
1.CP007176 − CP007178, GCA_000568475 − 1.CP007160, GCA_000568495 − 1.CP007004 − CP007006, GCA_000568815 −
1.CP007201, GCA_000568935 − 1.CP007210, GCA_000568955 − 1.CP006711, GCA_000568975 − 1.CP006712, GCA_000569015 −
1.CP006713, GCA_000569035 − 1.CP006714, GCA_000569055 − 1.CP006715, GCA_000569075 − 1.CP006716, GCA_000572125 −
1.CP002871, GCA_000572155 − 1.CP002882, GCA_000572175 − 1.CP002883, GCA_000572195 − 1.CP002885, GCA_000576085 −
1.CP003181, GCA_000576145 − 1.CP003182, GCA_000576165 − 1.CP003715 − CP003719, GCA_000576185 − 1.CP003183,
GCA_000576555 − 1.CP007143 − CP007146, GCA_000577275 − 1.HG916852 − HG916855, GCA_000577895 − 1.HG917868 −
HG917869, GCA_000582515 − 1.CP007230 − CP007231, GCA_000582535 − 1.CP006932, GCA_000582555 − 1.CP006829,
GCA_000582665 − 1.CP007122 − CP007126, GCA_000582845 − 1.HG421741, GCA_000583065 − 1.CP007244, GCA_000583105 −
1.CP006742 − CP006744, GCA_000583755 − 1.CP007183 − CP007187, GCA_000583775 − 1.CP007181 − CP007182, GCA_000583795 −

1.CP007179 − CP007180, GCA_000583835 − 1.CP004368 − CP004369, GCA_000583855 − 1.CP004373, GCA_000583875 − 1.CP006571 − CP006572, GCA_000585995 − 1.CP007410, GCA_000590455 − 1.CP007436, GCA_000590475 − 1.CP007441, GCA_000590555 − 1.CP006365, GCA_000590575 − 1.CP006671, GCA_000590615 − 1.CP006673, GCA_000590635 − 1.CP006674, GCA_000590675 − 1.CP006676, GCA_000590755 − 1.CP006680, GCA_000590775 − 1.CP006691, GCA_000590795 − 1.CP006896 − CP006897, GCA_000590815 − 1.CP006898 − CP006899, GCA_000590925 − 1.CP004372, GCA_000597785 − 2.CP009706, GCA_000597845 − 1.CP007265, GCA_000597865 − 1.CP007452 − CP007453, GCA_000597945 − 1.CP007448 − CP007450, GCA_000597965 − 1.CP007454 − CP007455, GCA_000597985 − 1.CP006872, GCA_000598005 − 1.CP006923 − CP006928, GCA_000599545 − 1.CP003949 − CP003958, GCA_000599625 − 1.CP007390, GCA_000599645 − 1.CP007391, GCA_000599665 − 1.CP007392, GCA_000599685 − 1.CP007393, GCA_000599705 − 1.CP007394, GCA_000599965 − 1.CP007482, GCA_000599985 − 1.CP007445, GCA_000600005 − 1.CP007446, GCA_000600015 − 1.CP007492, GCA_000600045 − 1.CP006820, GCA_000600055 − 1.CP006961 − CP006962, GCA_000600085 − 1.CP006821, GCA_000600105 − 1.CP006837, GCA_000600125 − 1.CP006822, GCA_000600145 − 1.CP006823, GCA_000600165 − 1.CP006824, GCA_000600185 − 1.CP006825, GCA_000600205 − 1.CP006826, GCA_000600225 − 1.CP006827, GCA_000604045 − 1.CP007502, GCA_000604125 − 1.CP003679, GCA_000612055 − 1.CP007519, GCA_000612075 − 1.CP007518, GCA_000612325 − 1.CP007507 − CP007508, GCA_000612485 − 1.CP007494, GCA_000612685 − 1.HG916765, GCA_000612765 − 1.HG969191, GCA_000612965 − 1.HG965802, GCA_000613085 − 1.HG813247 − HG813248, GCA_000623075 − 1.CP007270, GCA_000623095 − 1.CP007271, GCA_000623115 − 1.CP007272, GCA_000623135 − 1.CP007273, GCA_000623155 − 1.CP007274, GCA_000623175 − 1.CP007266, GCA_000623195 − 2.CP007267, GCA_000623275 − 1.CP007263, GCA_000623295 − 1.CP007258, GCA_000623315 − 1.CP007259, GCA_000623335 − 1.CP007260, GCA_000623355 − 1.CP007261, GCA_000623375 − 1.CP007262, GCA_000623755 − 1.CP007320, GCA_000623775 − 1.CP007321, GCA_000623795 − 1.CP007322, GCA_000624155 − 1.CP007332, GCA_000624395 − 2.CP007329, GCA_000626115 − 1.CP007420, GCA_000626135 − 1.CP007421, GCA_000626155 − 1.CP007422, GCA_000626175 − 1.CP007175, GCA_000626195 − 1.CP007245, GCA_000626215 − 1.CP007246, GCA_000626235 − 1.CP007247, GCA_000626255 − 1.CP007248, GCA_000626275 − 2.CP007249, GCA_000626295 − 1.CP007250, GCA_000626315 − 1.CP007251, GCA_000626335 − 1.CP007252, GCA_000626355 − 1.CP007253, GCA_000626375 − 1.CP007254, GCA_000626395 − 1.CP007438, GCA_000626415 − 1.CP007463, GCA_000626475 − 1.CP007466, GCA_000626495 − 1.CP007467, GCA_000626515 − 1.CP007468, GCA_000626535 − 1.CP007469, GCA_000626595 − 1.CP007524, GCA_000626615 − 1.CP007539, GCA_000626635 − 1.CP007451, GCA_000626675 − 1.CP006842 − CP006843, GCA_000632395 − 1.CP007546, GCA_000632415 − 1.CP004887, GCA_000632435 − 1.CP005081, GCA_000632475 − 2.CP007793 − CP007798, GCA_000632805 − 1.CP007444, GCA_000632815 − 1.CP007473, GCA_000632845 − 1.CP007474, GCA_000632865 − 1.CP007475, GCA_000632885 − 1.CP007476, GCA_000632905 − 1.CP007477, GCA_000632925 − 1.CP007478, GCA_000632945 − 1.CP007479, GCA_000632965 − 1.CP007480, GCA_000632985 − 1.CP007481, GCA_000633175 − 1.CP007566, GCA_000635955 − 1.CP007576, GCA_000636115 − 1.CP007565, GCA_000636135 − 1.CP007581 − CP007582, GCA_000648515 − 1.CP007557 − CP007558, GCA_000648525 − 1.CP007586, GCA_000648555 − 1.CP007587, GCA_000648735 − 2.CP011972 − CP011973, GCA_000661895 − 1.CP007514 − CP007517, GCA_000662395 − 1.CP007133 − CP007135, GCA_000671295 − 1.CP007136 − CP007138, GCA_000681515 − 1.CP007600, GCA_000685625 − 1.CP007603, GCA_000685665 − 1.CP007604, GCA_000685705 − 1.CP007605, GCA_000685725 − 1.CP006890, GCA_000685745 − 1.CP007606, GCA_000688775 − 2.CP007628, GCA_000689235 − 1.HG939456, GCA_000689355 − 1.HG969192 − HG969193, GCA_000689415 − 1.HG322950, GCA_000691545 − 1.CP007564, GCA_000691565 − 1.CP007620, GCA_000691585 − 1.CP007629 − CP007630, GCA_000691605 − 1.CP007656, GCA_000695215 − 1.CP007659, GCA_000695235 − 1.CP007574 − CP007575, GCA_000695835 − 1.CP007521, GCA_000695855 − 2.CP007535, GCA_000695875 − 1.CP007690, GCA_000695895 − 1.CP007755, GCA_000695935 − 1.CP007731 − CP007736, GCA_000695975 − 1.CP007243, GCA_000695995 − 1.CP005927, GCA_000696015 − 1.CP005933, GCA_000696465 − 1.CP007744, GCA_000696485 − 1.CP007757, GCA_000696505 − 1.CP006770, GCA_000698245 − 1.CP007691 − CP007692, GCA_000698285 − 1.CP007693 − CP007694, GCA_000698325 − 1.CP007697 − CP007698, GCA_000698345 − 1.CP007720 − CP007721, GCA_000698365 − 1.CP007805, GCA_000698475 − 1.CP007803, GCA_000698805 − 1.CP006696 − CP006697, GCA_000698825 − 1.CP006739 − CP006740, GCA_000698845 − 1.CP006986 − CP006991, GCA_000698865 − 1.CP008696, GCA_000698885 − 1.CP006819, GCA_000699025 − 1.CP007695 − CP007696, GCA_000699475 − 1.CP008699, GCA_000699505 − 1.CP007490, GCA_000706665 − 1.CP007809, GCA_000706685 − 1.CP007208 − CP007209, GCA_000706705 − 1.CP007409, GCA_000706725 − 1.CP003923, GCA_000706745 − 1.CP004383 − CP004384, GCA_000706765 − 1.CP006018, GCA_000709415 − 1.CP008724, GCA_000709435 − 1.CP008713, GCA_000709455 − 1.CP007276, GCA_000709475 − 1.CP007447, GCA_000709495 − 1.CP006032, GCA_000709535 − 1.CP006692, GCA_000709555 − 1.CP006873, GCA_000714595 − 1.CP007799, GCA_000714635 − 1.CP008700 − CP008701, GCA_000714655 − 1.CP008788 − CP008791, GCA_000714675 − 1.CP008797 − CP008800, GCA_000717515 − 1.CP008831 − CP008833, GCA_000717535 − 1.CP004870 − CP004883, GCA_000723165 − 1.HG322949, GCA_000723505 − 1.HG934468, GCA_000723785 − 1.HF677570 − HF677577, GCA_000724485 − 1.CP007739 − CP007741, GCA_000724505 − 1.CP008823 − CP008826, GCA_000724525 − 1.CP008841 − CP008845, GCA_000724585 − 1.CP008712, GCA_000724605 − 1.CP008802, GCA_000724625 − 1.CP007139, GCA_000724775 − 2.CP010820 − CP010821, GCA_000725265 − 1.CP005998 − CP006002, GCA_000725285 − 1.CP007161 − CP007164, GCA_000725305 − 1.CP008801, GCA_000725325 − 1.CP008846 − CP008848, GCA_000725345 − 1.CP008852, GCA_000725365 − 1.CP008876 −

CP008877, GCA_000725385 − 1.CP008884, GCA_000725405 − 1.CP008889 − CP008890, GCA_000730205 − 1.CP008885,
GCA_000730215 − 2.CP007631, GCA_000730245 − 1.CP004403, GCA_000730255 − 1.CP007632, GCA_000730345 − 1.CP008805 −
CP008807, GCA_000730365 − 1.CP008922, GCA_000730385 − 1.CP008849, GCA_000730405 − 1.CP008923, GCA_000730425 −
1.CP008896, GCA_000730445 − 1.CP008924, GCA_000731295 − 1.HG938355 − HG938357, GCA_000731315 − 1.HG938353 −
HG938354, GCA_000732355 − 1.CP008921, GCA_000732385 − 1.CP007024, GCA_000732425 − 1.CP007041, GCA_000732905 −
1.CP007588, GCA_000732925 − 1.CP008953 − CP008954, GCA_000732945 − 1.CP008944 − CP008945, GCA_000732965 −
1.CP008957 − CP008958, GCA_000733255 − 1.CP006798 − CP006802, GCA_000734015 − 1.CP008796, GCA_000734055 −
1.CP006649, GCA_000734895 − 2.CP011382 − CP011385, GCA_000734975 − 2.CP011052 − CP011054, GCA_000736415 −
1.CP009043 − CP009044, GCA_000737085 − 1.CP008787, GCA_000737325 − 2.CP011125, GCA_000737515 − 1.CP006587 −
CP006590, GCA_000737535 − 1.CP006269, GCA_000737555 − 1.CP006030 − CP006031, GCA_000737575 − 1.CP006270,
GCA_000737595 − 1.CP006271, GCA_000737615 − 1.CP006630, GCA_000737865 − 1.CP007287, GCA_000737885 − 1.CP007443,
GCA_000738435 − 1.CP003984, GCA_000738445 − 1.CP009100, GCA_000738475 − 1.CP009101, GCA_000739085 − 1.CP009110,
GCA_000739105 − 1.CP009124, GCA_000739375 − 1.CP006704, GCA_000739395 − 1.CP007627, GCA_000739435 − 1.CP009159,
GCA_000739455 − 1.CP009211, GCA_000739475 − 1.CP009117 − CP009119, GCA_000739495 − 1.CP009114 − CP009116,
GCA_000740135 − 1.CP007680 − CP007681, GCA_000740155 − 1.CP007682 − CP007683, GCA_000740175 − 1.CP007700 −
CP007701, GCA_000740195 − 1.CP007705 − CP007706, GCA_000740215 − 1.CP007709 − CP007710, GCA_000740235 −
1.CP007716 − CP007717, GCA_000740255 − 1.CP007718 − CP007719, GCA_000740275 − 1.CP007742 − CP007743, GCA_000740295 −
1.CP007662 − CP007663, GCA_000740315 − 1.CP007737 − CP007738, GCA_000740335 − 1.CP007758 − CP007759, GCA_000740355 −
1.CP007760 − CP007761, GCA_000740375 − 1.CP007764 − CP007765, GCA_000740415 − 1.CP007762 − CP007763, GCA_000740435 −
1.CP008756 − CP008757, GCA_000740455 − 1.CP008804, GCA_000740965 − 1.CP009125 − CP009126, GCA_000740985 −
1.CP009158, GCA_000741005 − 1.CP009212 − CP009214, GCA_000742655 − 1.CP007617 − CP007618, GCA_000742715 −
1.CP007722 − CP007723, GCA_000742735 − 1.CP007724 − CP007725, GCA_000742755 − 1.CP009208, GCA_000742795 −
1.CP009237, GCA_000742815 − 1.CP007639, GCA_000742835 − 1.CP008941 − CP008942, GCA_000742855 − 1.CP007621 −
CP007626, GCA_000742895 − 1.CP007664 − CP007666, GCA_000742955 − 1.CP008817 − CP008820, GCA_000742975 −
1.CP008814 − CP008816, GCA_000743015 − 1.CP008926, GCA_000743035 − 1.CP008936 − CP008940, GCA_000743055 −
1.CP009102 − CP009103, GCA_000743255 − 1.CP009072 − CP009074, GCA_000743945 − 1.CP009238, GCA_000743955 −
1.CP004056, GCA_000743995 − 1.CP004057, GCA_000746505 − 1.CP007499 − CP007500, GCA_000746525 − 1.CP009048,
GCA_000746585 − 2.CP009217 − CP009219, GCA_000746605 − 1.CP009256, GCA_000746625 − 1.CP009258, GCA_000746645 −
1.CP009257, GCA_000746665 − 1.CP009261 − CP009263, GCA_000747295 − 1.CP009144 − CP009146, GCA_000747315 −
1.CP009215 − CP009216, GCA_000747335 − 1.CP008853, GCA_000747345 − 1.CP008855, GCA_000747375 − 1.CP008854,
GCA_000747525 − 1.CP009278, GCA_000747545 − 1.CP007607 − CP007616, GCA_000747565 − 1.CP003424 − CP003425,
GCA_000750195 − 1.CP009239, GCA_000750215 − 1.CP009089, GCA_000750255 − 1.CP009090, GCA_000750295 − 1.CP009091,
GCA_000750315 − 1.CP008943, GCA_000750335 − 1.CP009092, GCA_000750375 − 1.CP009093, GCA_000750395 − 2.CP009085,
GCA_000750415 − 2.CP009084, GCA_000750435 − 1.CP009086, GCA_000750455 − 1.CP009087, GCA_000750475 − 1.CP009083,
GCA_000750495 − 1.CP009088, GCA_000750515 − 1.CP009223, GCA_000750535 − 1.CP009224, GCA_000751035 − 1.HG813242 −
HG813246, GCA_000754265 − 1.CP008985, GCA_000754275 − 1.CP008918, GCA_000754305 − 1.CP008986, GCA_000754345 −
1.CP008919 − CP008920, GCA_000754365 − 1.CP007206, GCA_000754375 − 1.CP008927 − CP008928, GCA_000754405 −
1.CP007207, GCA_000755085 − 1.CP009096 − CP009097, GCA_000755105 − 1.CP009094 − CP009095, GCA_000755145 −
1.CP009228 − CP009229, GCA_000755185 − 1.CP008913, GCA_000755705 − 1.CP009415, GCA_000755725 − 1.CP007782 −
CP007784, GCA_000755765 − 1.CP009209 − CP009210, GCA_000755785 − 1.CP009147 − CP009148, GCA_000755805 −
1.CP007785 − CP007787, GCA_000755825 − 1.CP009127 − CP009128, GCA_000755845 − 1.CP008710 − CP008711, GCA_000755865 −
1.CP008704 − CP008705, GCA_000755885 − 1.CP008722 − CP008723, GCA_000755905 − 1.CP008893 − CP008894, GCA_000755925 −
1.CP008911 − CP008912, GCA_000755945 − 1.CP008891 − CP008892, GCA_000755965 − 1.CP008909 − CP008910, GCA_000755985 −
1.CP008726 − CP008727, GCA_000756005 − 1.CP008728 − CP008730, GCA_000756025 − 2.CP008731 − CP008732, GCA_000756045 −
1.CP008760 − CP008762, GCA_000756065 − 1.CP008783 − CP008784, GCA_000756085 − 1.CP008834 − CP008835, GCA_000756125 −
1.CP008781 − CP008782, GCA_000756165 − 1.CP008779 − CP008780, GCA_000756185 − 1.CP008777 − CP008778, GCA_000756205 −
1.CP009361 − CP009362, GCA_000756465 − 1.CP006693, GCA_000756485 − 1.CP008695, GCA_000756505 − 1.CP006828,
GCA_000756545 − 1.CP009427, GCA_000756615 − 1.CP009288 − CP009289, GCA_000756715 − 2.CCSE01000001, GCA_000756775 −
2.CCSF01000001, GCA_000757015 − 2.CP009585 − CP009586, GCA_000757035 − 2.CP009898 − CP009899, GCA_000757785 −
1.CP009450, GCA_000757795 − 1.CP003811, GCA_000757825 − 1.CP009451, GCA_000757845 − 1.CP007753, GCA_000757865 −
1.CP007754, GCA_000758225 − 1.CP009447, GCA_000758245 − 1.CP009449, GCA_000758265 − 1.CP009448, GCA_000758285 −
1.CP009493, GCA_000758305 − 1.CP009458, GCA_000758325 − 1.CP009459, GCA_000758345 − 1.CP009460, GCA_000758365 −
1.CP007646 − CP007650, GCA_000758385 − 1.CP009407, GCA_000758405 − 1.CP009408, GCA_000758525 − 1.CP009241,
GCA_000758545 − 1.CP009279, GCA_000758565 − 1.CP009280, GCA_000758585 − 1.CP009281, GCA_000758605 − 1.CP009282,
GCA_000758625 − 1.CP009283, GCA_000758645 − 1.CP009284, GCA_000758665 − 1.CP009285, GCA_000758685 − 1.CP009286,

GCA_000758705 − 1.CP009287, GCA_000758725 − 1.CP009428, GCA_000759475 − 1.CP009454, GCA_000759485 − 1.CP008808 − CP008809, GCA_000759515 − 1.CP008810 − CP008812, GCA_000759535 − 1.CP009455, GCA_000759555 − 1.CP009046 − CP009047, GCA_000761115 − 1.CP009472, GCA_000761135 − 1.CP009531 − CP009532, GCA_000761155 − 1.CP009533, GCA_000761175 − 1.CP009534, GCA_000761195 − 1.CP009290, GCA_000761215 − 1.CP009438 − CP009439, GCA_000762285 − 1.CP007801 − CP007802, GCA_000763475 − 1.CP009575, GCA_000763495 − 1.CP009576, GCA_000763515 − 1.CP009577, GCA_000763535 − 2.CP009264 − CP009266, GCA_000763575 − 1.CP009170, GCA_000764535 − 1.CP009571 − CP009573, GCA_000764555 − 1.CP009574, GCA_000764575 − 1.CP004377 − CP004378, GCA_000764595 − 1.CP004385 − CP004386, GCA_000764615 − 1.CP008929 − CP008933, GCA_000764955 − 1.CP007779 − CP007781, GCA_000765375 − 1.CP004381 − CP004382, GCA_000765395 − 1.CP007495, GCA_000765415 − 1.CP007634 − CP007636, GCA_000766665 − 1.CP007669, GCA_000767055 − 1.CP006764 − CP006765, GCA_000767075 − 1.CP009610, GCA_000767095 − 1.CP008902, GCA_000767405 − 1.CP009608, GCA_000767415 − 1.CP009500, GCA_000767445 − 1.CP009609, GCA_000767465 − 1.CP009291 − CP009296, GCA_000767485 − 1.CP009499, GCA_000767505 − 1.CP007240, GCA_000767605 − 1.CP009494, GCA_000767615 − 1.CP009553, GCA_000767645 − 1.CP009583 − CP009584, GCA_000767665 − 1.CP009495, GCA_000767685 − 1.CP009622, GCA_000767705 − 1.CP009496, GCA_000767725 − 1.CP009652, GCA_000767745 − 1.CP006777, GCA_000769535 − 1.CP009678, GCA_000769555 − 1.CP009679 − CP009680, GCA_000769575 − 1.CP009681, GCA_000769635 − 1.CP009716, GCA_000770125 − 1.CP009613, GCA_000770155 − 1.CP009756, GCA_000770175 − 1.CP009615, GCA_000770195 − 1.CP007711, GCA_000770215 − 1.CP009616, GCA_000770235 − 1.CP009614, GCA_000770395 − 1.CP009234 − CP009235, GCA_000770455 − 1.CP009150 − CP009151, GCA_000770495 − 1.CP009160 − CP009161, GCA_000770515 − 1.CP009162 − CP009163, GCA_000770535 − 1.CP009164 − CP009165, GCA_000770565 − 1.CP009154 − CP009157, GCA_000772025 − 1.CP009554, GCA_000772065 − 1.CP009617 − CP009620, GCA_000772105 − 1.CP009354 − CP009359, GCA_000772125 − 1.CP009748, GCA_000772145 − 1.CP009760, GCA_000772165 − 1.CP009749, GCA_000772185 − 1.CP009612, GCA_000772205 − 1.CP009611, GCA_000772225 − 1.CP006851, GCA_000772245 − 1.CP007241, GCA_000772485 − 1.CP006741, GCA_000775375 − 1.CP009771 − CP009774, GCA_000775395 − 1.CP009775 − CP009778, GCA_000775955 − 1.CP009461, GCA_000775975 − 1.CP009746, GCA_000775995 − 1.CP009227, GCA_000782835 − 1.CP009796, GCA_000782855 − 1.CP006910, GCA_000784865 − 1.CP009850 − CP009853, GCA_000784905 − 1.CP009854 − CP009858, GCA_000784925 − 1.CP009859 − CP009862, GCA_000784945 − 1.CP009863 − CP009865, GCA_000784965 − 1.CP009866 − CP009871, GCA_000784985 − 1.CP009872 − CP009875, GCA_000785005 − 1.CP009876 − CP009879, GCA_000785495 − 1.CP009896, GCA_000785515 − 1.CP009913, GCA_000785535 − 1.CP007595, GCA_000785555 − 1.CP009129 − CP009130, GCA_000786505 − 1.HG813240, GCA_000786695 − 1.CP009962 − CP009963, GCA_000786735 − 1.CP007712 − CP007714, GCA_000789275 − 1.CP010052, GCA_000789295 − 1.CP010053, GCA_000789315 − 1.CP009939 − CP009941, GCA_000789335 − 1.CP003408, GCA_000789355 − 1.CP006903 − CP006904, GCA_000789375 − 1.CP003409, GCA_000789395 − 1.CP006905 − CP006906, GCA_000800235 − 1.CP009418, GCA_000800255 − 1.CP009747, GCA_000800275 − 1.CP009419, GCA_000800295 − 1.CP009761, GCA_000800315 − 1.CP009420, GCA_000800335 − 1.CP009897, GCA_000800355 − 1.CP009421, GCA_000800395 − 1.CP010028, GCA_000800415 − 1.CP009422, GCA_000800435 − 1.CP010105, GCA_000800455 − 1.CP007456, GCA_000800475 − 1.CP007457 − CP007458, GCA_000800785 − 1.CP010267, GCA_000800825 − 1.CP010075, GCA_000801145 − 1.CP007715, GCA_000801165 − 1.CP009104 − CP009105, GCA_000801185 − 2.CP009106 − CP009107, GCA_000801205 − 1.CP009685, GCA_000801315 − 1.CP010054 − CP010057, GCA_000803625 − 1.CP007496, GCA_000803645 − 1.CP009122 − CP009123, GCA_000803665 − 1.CP010089 − CP010099, GCA_000803705 − 1.CP010304 − CP010305, GCA_000807175 − 1.CP009267, GCA_000807225 − 1.CP010327, GCA_000807255 − 1.CP009268, GCA_000807275 − 1.CP009056, GCA_000807295 − 1.CP010308, GCA_000807315 − 1.CP010313, GCA_000807355 − 1.CP010058, GCA_000807375 − 1.CP010050, GCA_000808035 − 2.CP008914 − CP008915, GCA_000808055 − 1.CP010346, GCA_000808095 − 1.CP009910 − CP009912, GCA_000812165 − 1.CP010323, GCA_000812205 − 1.CP009274 − CP009276, GCA_000813165 − 1.CP005930 − CP005932, GCA_000813205 − 1.CP006738, GCA_000814125 − 2.CP010374 − CP010376, GCA_000814145 − 2.CP010371 − CP010373, GCA_000814165 − 1.CP010368 − CP010370, GCA_000814205 − 1.CP010377 − CP010380, GCA_000814225 − 1.CP010384 − CP010387, GCA_000814265 − 1.CP009226, GCA_000814305 − 1.CP010392 − CP010396, GCA_000814345 − 1.CP010397 − CP010400, GCA_000814805 − 1.CP006722, GCA_000814825 − 1.CP009302, GCA_000814845 − 1.CP010431, GCA_000814865 − 1.CP009927, GCA_000814885 − 1.CP006809 − CP006810, GCA_000815025 − 1.CP007541, GCA_000815045 − 1.CP010295, GCA_000815065 − 1.CP007585, GCA_000815085 − 1.CP010296, GCA_000815105 − 1.CP010310, GCA_000815125 − 1.CP010297, GCA_000815145 − 1.CP010406, GCA_000815165 − 1.CP010298, GCA_000815185 − 1.CP010409 − CP010410, GCA_000815205 − 1.CP010299, GCA_000815225 − 1.CP010427 − CP010428, GCA_000815245 − 1.CP010300, GCA_000816025 − 1.CP003987 − CP003988, GCA_000816045 − 1.CP006929, GCA_000816065 − 1.CP007003, GCA_000816085 − 1.CP008747, GCA_000816125 − 1.CP007641 − CP007645, GCA_000816165 − 1.CP006869, GCA_000816185 − 1.CP007770, GCA_000816205 − 1.CP007522, GCA_000816225 − 1.CP007771, GCA_000816245 − 1.CP007769, GCA_000816265 − 1.CP007772, GCA_000816305 − 1.CP007773, GCA_000816345 − 1.CP007774, GCA_000816365 − 1.CP007775, GCA_000816385 − 1.CP007776, GCA_000816405 − 1.CP007777, GCA_000816425 − 1.CP007778, GCA_000816785 − 1.CP007766 − CP007768, GCA_000816805 − 1.CP010434, GCA_000816845 − 1.CP006877 − CP006880, GCA_000816885 − 1.CP006855 − CP006857, GCA_000816945 − 1.CP006907, GCA_000816985 − 1.CP007399, GCA_000817005 −

1.CP007593, GCA_000817025 – 1.CP010013, GCA_000817045 – 1.CP010433, GCA_000817065 – 1.CP007016, GCA_000817325 – 1.CP006471 – CP006473, GCA_000817935 – 1.CP006908 – CP006909, GCA_000817955 – 1.CP009788, GCA_000817975 – 1.CP010415 – CP010421, GCA_000817995 – 1.CP010437, GCA_000818015 – 1.CP007511, GCA_000818035 – 1.CP007726, GCA_000818055 – 1.CP009045, GCA_000818075 – 1.CP009049, GCA_000818095 – 1.CP009416 – CP009417, GCA_000818115 – 1.CP009559, GCA_000819445 – 1.CP005286, GCA_000819505 – 1.CP006883, GCA_000819525 – 1.CP006957, GCA_000819565 – 1.CP004393 – CP004398, GCA_000819645 – 1.CP010315 – CP010318, GCA_000819665 – 1.CP010268 – CP010270, GCA_000826965 – 3.CP010518, GCA_000826985 – 1.CP010435, GCA_000827005 – 1.CP010519, GCA_000827025 – 1.CP010436, GCA_000827065 – 1.CP010314, GCA_000827085 – 1.CP007027, GCA_000827125 – 1.CP010311 – CP010312, GCA_000827855 – 1.CP007563, GCA_000827935 – 1.CP010520, GCA_000827955 – 1.CP010521, GCA_000828015 – 1.CP010451 – CP010452, GCA_000828035 – 1.CP010526, GCA_000828055 – 1.CP010523, GCA_000828475 – 1.AP014648, GCA_000828635 – 1.AP012547, GCA_000828695 – 1.AP014522, GCA_000828715 – 1.AP014583, GCA_000828735 – 1.AP014584, GCA_000828795 – 1.AP013357, GCA_000828815 – 1.AP014521, GCA_000828835 – 1.AP014633, GCA_000828855 – 1.AP014631, GCA_000828875 – 1.AP014576 – AP014581, GCA_000828895 – 1.AP014568, GCA_000828915 – 1.AP014569 – AP014570, GCA_000828935 – 1.AP014649 – AP014650, GCA_000828975 – 1.AP014683, GCA_000828995 – 1.AP014573, GCA_000829035 – 1.AP012541 – AP012543, GCA_000829055 – 1.AP012544 – AP012546, GCA_000829075 – 1.AP012555 – AP012556, GCA_000829155 – 1.AP013293, GCA_000829175 – 1.AP014582, GCA_000829195 – 1.AP013294, GCA_000829215 – 1.AP014524 – AP014525, GCA_000829255 – 1.AP014646, GCA_000829275 – 1.AP014622, GCA_000829295 – 1.AP014658, GCA_000829315 – 1.AP013028, GCA_000829335 – 1.AP013353, GCA_000829355 – 1.AP014595, GCA_000829375 – 1.AP014624, GCA_000829395 – 1.AP014680 – AP014682, GCA_000829415 – 1.AP014637, GCA_000829885 – 1.CP010555, GCA_000829965 – 1.CP010557, GCA_000829985 – 1.CP009166 – CP009169, GCA_000830005 – 1.CP010407 – CP010408, GCA_000830035 – 1.CP010585, GCA_000830075 – 1.CP010778, GCA_000830095 – 1.CP010792 – CP010794, GCA_000830775 – 1.CP006685, GCA_000830805 – 1.CP006686, GCA_000830825 – 1.CP006687, GCA_000830845 – 1.CP006688, GCA_000830865 – 1.CP006689, GCA_000830885 – 1.CP006951, GCA_000830905 – 1.CP006950, GCA_000830925 – 1.CP006949, GCA_000830945 – 1.CP006974, GCA_000830965 – 1.CP006975, GCA_000830985 – 1.CP007151, GCA_000831005 – 1.CP007152, GCA_000831025 – 1.CP007359, GCA_000831045 – 1.CP007358, GCA_000831065 – 1.CP007512 – CP007513, GCA_000831105 – 1.CP007572, GCA_000831125 – 1.CP007571, GCA_000831145 – 1.CP007570, GCA_000831165 – 1.CP007573, GCA_000831185 – 1.CP008881, GCA_000831205 – 1.CP008883, GCA_000831225 – 1.CP008878, GCA_000831245 – 1.CP009480, GCA_000831265 – 1.CP009481, GCA_000831285 – 1.CP009482, GCA_000831305 – 1.CP009483, GCA_000831405 – 1.CP007232, GCA_000831425 – 1.CP007233, GCA_000831465 – 1.CP007234, GCA_000831485 – 1.CP010783, GCA_000831525 – 1.CP006009, GCA_000831545 – 1.CP006010, GCA_000831565 – 1.CP010344 – CP010345, GCA_000831585 – 1.CP010359, GCA_000832145 – 1.CP007633, GCA_000832305 – 1.CP010536 – CP010537, GCA_000832385 – 1.CP009299 – CP009300, GCA_000832405 – 1.CP009317 – CP009318, GCA_000832425 – 1.CP009324 – CP009325, GCA_000832445 – 1.CP009326 – CP009328, GCA_000832465 – 1.CP009329 – CP009331, GCA_000832485 – 1.CP009332 – CP009336, GCA_000832505 – 1.CP009339 – CP009341, GCA_000832525 – 1.CP009368 – CP009369, GCA_000832565 – 1.CP009462 – CP009464, GCA_000832585 – 1.CP009475 – CP009476, GCA_000832605 – 1.CP009689 – CP009692, GCA_000832635 – 1.CP009540 – CP009541, GCA_000832665 – 1.CP009542 – CP009544, GCA_000832725 – 1.CP009698 – CP009700, GCA_000832745 – 1.CP009695 – CP009697, GCA_000832765 – 1.CP009593 – CP009596, GCA_000832785 – 1.CP009597 – CP009598, GCA_000832805 – 1.CP009589 – CP009592, GCA_000832825 – 1.CP009599 – CP009600, GCA_000832845 – 1.CP009627 – CP009628, GCA_000832865 – 1.CP009634 – CP009641, GCA_000832885 – 1.CP009645 – CP009651, GCA_000832905 – 1.CP009709, GCA_000832925 – 1.CP009717 – CP009720, GCA_000832965 – 1.CP009900 – CP009902, GCA_000832985 – 1.CP009915 – CP009921, GCA_000833005 – 1.CP010556, GCA_000833025 – 1.CP010817, GCA_000833045 – 1.CP009965 – CP009970, GCA_000833065 – 1.CP009979 – CP009981, GCA_000833085 – 1.CP010087 – CP010088, GCA_000833125 – 1.CP010320 – CP010322, GCA_000833145 – 1.CP010816, GCA_000833165 – 1.CP009352 – CP009353, GCA_000833195 – 1.CP009342 – CP009343, GCA_000833215 – 1.CP009436 – CP009437, GCA_000833235 – 1.CP009693, GCA_000833255 – 1.CP009440 – CP009441, GCA_000833275 – 1.CP009314 – CP009316, GCA_000833295 – 1.CP009442 – CP009443, GCA_000833315 – 1.CP009444 – CP009446, GCA_000833335 – 1.CP009694, GCA_000833355 – 1.CP009607, GCA_000833375 – 1.CP009633, GCA_000833455 – 1.CP010019, GCA_000833475 – 1.CP010115, GCA_000833495 – 1.CP010288, GCA_000833515 – 1.CP010289, GCA_000833535 – 1.CP010290, GCA_000833575 – 1.CP010827, GCA_000834195 – 1.CP009366 – CP009367, GCA_000834215 – 1.CP009363 – CP009364, GCA_000834235 – 1.CP009489 – CP009492, GCA_000834255 – 1.CP009539, GCA_000834275 – 1.CP009701 – CP009704, GCA_000834295 – 1.CP009710 – CP009712, GCA_000834315 – 1.CP009713 – CP009715, GCA_000834335 – 1.CP009721 – CP009724, GCA_000834355 – 1.CP009757, GCA_000834375 – 1.CP009792, GCA_000834415 – 1.CP009758 – CP009759, GCA_000834435 – 1.CP009786, GCA_000834455 – 1.CP009787, GCA_000834475 – 1.CP009779 – CP009780, GCA_000834495 – 1.CP009782 – CP009785, GCA_000834515 – 1.CP009801, GCA_000834735 – 1.CP009837 – CP009838, GCA_000834755 – 1.CP009839 – CP009841, GCA_000834775 – 1.CP009842 – CP009844, GCA_000834825 – 1.CP009903 – CP009906, GCA_000834845 – 1.CP009934 – CP009937, GCA_000834865 – 1.CP009997, GCA_000834885 – 1.CP009988 – CP009991, GCA_000834925 – 1.CP010020 – CP010023, GCA_000834945 – 1.CP010067 – CP010069, GCA_000834965 – 1.CP010103 – CP010104, GCA_000834985 – 1.CP010246 – CP010248, GCA_000835025 –

1.CP009344 – CP009351, GCA_000835085 – 1.CP006959, GCA_000835145 – 1.CP007242, GCA_000835165 – 1.CP007790 – CP007792, GCA_000835185 – 1.CP009603 – CP009606, GCA_000835205 – 1.CP002580 – CP002581, GCA_000835225 – 1.CP010113, GCA_000835265 – 1.CP010114, GCA_000835285 – 1.CP010306, GCA_000835305 – 1.CP010307, GCA_000835345 – 1.CP010072 – CP010074, GCA_000835365 – 1.CP010301 – CP010303, GCA_000875675 – 1.CP010848, GCA_000875695 – 1.CP010850 – CP010851, GCA_000875715 – 1.CP010852 – CP010854, GCA_000875755 – 1.CP007520, GCA_000877815 – 1.CP010014 – CP010015, GCA_000931425 – 1.CP010528, GCA_000931445 – 1.CP010849, GCA_000931565 – 1.CP010876 – CP010882, GCA_000931575 – 1.CP007470, GCA_000931605 – 1.CP007471, GCA_000931625 – 1.CP007472, GCA_000932055 – 1.CP010905, GCA_000934305 – 1.CP010906, GCA_000934525 – 1.CP010912, GCA_000934545 – 1.CP010554, GCA_000934565 – 1.CP010896, GCA_000934605 – 1.CP010897 – CP010898, GCA_000940785 – 1.CP010577 – CP010583, GCA_000940805 – 1.CP007142, GCA_000940825 – 1.CP007221, GCA_000940845 – 1.CP010341, GCA_000940895 – 1.CP007211, GCA_000940915 – 1.CP010947 – CP010950, GCA_000940935 – 1.CP007216, GCA_000940975 – 1.CP007222, GCA_000940995 – 1.CP005973 – CP005974, GCA_000941015 – 1.CP007235, GCA_000941035 – 1.CP006723 – CP006727, GCA_000941055 – 1.CP007202, GCA_000941075 – 1.CP007229, GCA_000943805 – 1.CP010954 – CP010960, GCA_000949425 – 1.CP010976 – CP010977, GCA_000950775 – 1.CP011004, GCA_000952035 – 1.CP007699, GCA_000952915 – 1.LM995445, GCA_000952975 – 1.LM997412, GCA_000953035 – 1.LM651928, GCA_000953135 – 1.LN614827 – LN614829, GCA_000953195 – 1.LK028559, GCA_000953215 – 1.LM995447, GCA_000953235 – 1.LN515399, GCA_000953255 – 1.LN626917, GCA_000953315 – 1.LK055284, GCA_000953355 – 1.LN681227 – LN681228, GCA_000953375 – 1.LN515398, GCA_000953415 – 1.LN734649, GCA_000953435 – 1.LM655252, GCA_000953455 – 1.LK391695, GCA_000953495 – 1.LN649235, GCA_000953535 – 1.LN515532, GCA_000953575 – 1.LN554884, GCA_000953635 – 1.LN614830, GCA_000953655 – 1.LN681225 – LN681226, GCA_000953695 – 1.LN554846 – LN554851, GCA_000953715 – 1.LN824141, GCA_000953735 – 1.LN554852 – LN554854, GCA_000954115 – 1.CP010797, GCA_000954135 – 2.CP010799 – CP010801, GCA_000954155 – 1.CP010873, GCA_000954175 – 1.CP010973 – CP010974, GCA_000954195 – 1.CP011015 – CP011017, GCA_000955665 – 1.CP011059, GCA_000956315 – 1.CP011060, GCA_000958465 – 1.CP011043 – CP011046, GCA_000959025 – 1.CP011051, GCA_000959125 – 1.CP004379 – CP004380, GCA_000959145 – 1.CP009297 – CP009298, GCA_000959165 – 1.CP009337 – CP009338, GCA_000959185 – 1.CP009473 – CP009474, GCA_000959205 – 1.CP009484 – CP009485, GCA_000959225 – 1.CP009477 – CP009478, GCA_000959245 – 1.CP009486 – CP009488, GCA_000959265 – 1.CP009535 – CP009536, GCA_000959285 – 1.CP009537 – CP009538, GCA_000959305 – 1.CP009545 – CP009546, GCA_000959325 – 1.CP009547 – CP009549, GCA_000959345 – 1.CP009550 – CP009551, GCA_000959365 – 1.CP009555 – CP009556, GCA_000959405 – 1.CP009587 – CP009588, GCA_000959425 – 1.CP009601 – CP009602, GCA_000959445 – 1.CP009629 – CP009632, GCA_000959465 – 1.CP009642 – CP009643, GCA_000959485 – 1.CP009707 – CP009708, GCA_000959505 – 1.CP009793 – CP009795, GCA_000959525 – 1.CP009830 – CP009832, GCA_000959545 – 1.CP009797 – CP009800, GCA_000959625 – 1.CP010065 – CP010066, GCA_000959725 – 1.CP009319 – CP009323, GCA_000960995 – 1.CP009432 – CP009435, GCA_000961015 – 1.CP009907 – CP009908, GCA_000961095 – 1.CP011058, GCA_000961155 – 1.CP008987 – CP008989, GCA_000961175 – 1.CP008990 – CP008992, GCA_000961195 – 1.CP008993 – CP008995, GCA_000961215 – 1.CP008996 – CP008998, GCA_000961235 – 1.CP008999 – CP009001, GCA_000961255 – 1.CP009002 – CP009004, GCA_000961275 – 1.CP009005 – CP009007, GCA_000961295 – 1.CP009008 – CP009010, GCA_000961315 – 1.CP009011 – CP009013, GCA_000961335 – 1.CP009014 – CP009016, GCA_000961355 – 1.CP009017 – CP009019, GCA_000961375 – 1.CP009020 – CP009022, GCA_000961395 – 1.CP009023 – CP009025, GCA_000961415 – 1.CP009026 – CP009028, GCA_000961435 – 1.CP009029 – CP009031, GCA_000961455 – 1.CP009032 – CP009034, GCA_000961475 – 1.CP009035 – CP009037, GCA_000961495 – 1.CP009038 – CP009040, GCA_000961515 – 1.CP010024 – CP010027, GCA_000962775 – 1.CP009058 – CP009071, GCA_000963515 – 1.CP006871, GCA_000963535 – 1.CP007483, GCA_000963555 – 1.CP009041 – CP009042, GCA_000963575 – 1.CP011077, GCA_000963635 – 1.CP011098, GCA_000963645 – 1.CP011100, GCA_000963675 – 1.CP011099, GCA_000963815 – 1.CP008706 – CP008709, GCA_000963835 – 1.CP011110 – CP011111, GCA_000963865 – 1.CP011071, GCA_000964565 – 1.CP011078, GCA_000965765 – 1.CP011021, GCA_000965785 – 1.CP011073, GCA_000967115 – 1.HG530135 – HG530136, GCA_000967135 – 1.HG004426 – HG004427, GCA_000967285 – 1.AM412059, GCA_000967325 – 1.HE579059 – HE579060, GCA_000967345 – 1.HE579061 – HE579062, GCA_000967365 – 1.HE579063 – HE579064, GCA_000967385 – 1.HE579069 – HE579070, GCA_000967405 – 1.HE579071 – HE579072, GCA_000967425 – 1.FO082820 – FO082822, GCA_000967445 – 1.FO393392, GCA_000967895 – 1.FO203512, GCA_000967915 – 1.FO681348, GCA_000968055 – 1.FO681347, GCA_000968155 – 1.FO834904 – FO834906, GCA_000968175 – 1.FO704551, GCA_000968195 – 1.FO704549 – FO704550, GCA_000968335 – 1.CP008740, GCA_000968375 – 1.CP009933, GCA_000968415 – 1.CP011117, GCA_000968515 – 1.CP011134 – CP011143, GCA_000968535 – 1.FO082060 – FO082061, GCA_000968945 – 1.CP011095, GCA_000969225 – 1.CP011147, GCA_000969235 – 1.CP010811, GCA_000969265 – 1.CP010812, GCA_000969685 – 1.FO681494 – FO681497, GCA_000969765 – 1.CP011096, GCA_000971565 – 1.CP011246 – CP011249, GCA_000971575 – 1.CP010274, GCA_000971615 – 1.CP011018 – CP011019, GCA_000971645 – 1.CP010276, GCA_000971665 – 1.CP011217, GCA_000971705 – 1.CP009923 – CP009924, GCA_000971725 – 1.CP009925 – CP009926, GCA_000971765 – 1.CP010907 – CP010911, GCA_000971785 – 1.CP010798, GCA_000971925 – 1.CP011115, GCA_000972245 – 2.CP011974, GCA_000972685 – 1.CP011150, GCA_000972725 – 1.CP011116, GCA_000972745 – 1.CP011256, GCA_000972785 – 2.CP011253, GCA_000972805 – 1.CP010889, GCA_000972865 – 1.CP011144, GCA_000973085 – 1.CP011280,

GCA_000973105 − 1.FP476056, GCA_000973125 − 1.FO818637 − FO818639, GCA_000973485 − 1.CP011278, GCA_000973525 −
1.CP004391, GCA_000973565 − 1.CP004392, GCA_000973625 − 1.CP007501, GCA_000973645 − 1.CP007523, GCA_000973665 −
1.CP007540, GCA_000973685 − 1.CP007559, GCA_000973705 − 1.CP009225, GCA_000973725 − 1.CP009621, GCA_000974405 −
1.CP010438, GCA_000974425 − 1.CP010429, GCA_000974465 − 1.CP010439, GCA_000974505 − 1.CP010440, GCA_000974535 −
1.CP010441, GCA_000974575 − 1.CP010442, GCA_000974825 − 1.CP010443, GCA_000974835 − 1.CP011301 − CP011302,
GCA_000974865 − 1.CP010444, GCA_000974885 − 1.CP010445, GCA_000975175 − 1.CP011295 − CP011298, GCA_000975245 −
1.CP011303, GCA_000978375 − 1.CP009686, GCA_000978535 − 1.CP010967, GCA_000978555 − 1.CP011107, GCA_000978575 −
1.CP011108, GCA_000978785 − 2.CP010446 − CP010447, GCA_000980815 − 1.CP011311, GCA_000980835 − 1.CP011312,
GCA_000981585 − 1.LN831776, GCA_000981765 − 1.CP010975, GCA_000981805 − 1.CP011132 − CP011133, GCA_000981825 −
1.CP011317, GCA_000981845 − 1.CP011313 − CP011316, GCA_000982695 − 1.CP011330, GCA_000982715 − 1.CP011341,
GCA_000986765 − 1.CP011331 − CP011338, GCA_000987825 − 1.CP011346, GCA_000987835 − 1.CP011308, GCA_000987865 −
1.CP011309, GCA_000987875 − 1.CP007594, GCA_000987925 − 1.CP011118 − CP011119, GCA_000988065 − 1.CP011368,
GCA_000988345 − 1.CP011347, GCA_000988395 − 1.CP006256 − CP006257, GCA_000988485 − 1.CP005969, GCA_000988525 −
1.CP007584, GCA_000993725 − 1.CP011365, GCA_000993745 − 1.CP007497, GCA_000993765 − 1.CP007537, GCA_000993785 −
2.CP009922, GCA_000993825 − 1.CP011114, GCA_001005905 − 1.CP011366, GCA_001005925 − 1.CP011398 − CP011399,
GCA_001005985 − 1.CP011397, GCA_001006005 − 1.CP011254, GCA_001006025 − 1.CP011386, GCA_001006525 − 1.CP011428 −
CP011435, GCA_001007025 − 1.LN846980, GCA_001007045 − 1.LN847221, GCA_001007065 − 1.LN847000, GCA_001007085 −
1.LN847257, GCA_001007105 − 1.LN846996, GCA_001007125 − 1.LN846995, GCA_001007875 − 1.CP011412 − CP011413,
GCA_001007915 − 1.CP011416 − CP011418, GCA_001007935 − 1.CP011451, GCA_001007995 − 1.CP011389, GCA_001008015 −
1.CP010999, GCA_001008165 − 1.CP011452 − CP011453, GCA_001010285 − 1.CP011104, GCA_001010485 − 1.AP014722,
GCA_001010505 − 1.AP014613, GCA_001010765 − 1.CP011410 − CP011411, GCA_001010785 − 1.CP011279, GCA_001011035 −
1.CP011492, GCA_001011055 − 1.CP011359, GCA_001011095 − 1.CP011403 − CP011405, GCA_001011135 − 1.CP009753,
GCA_001013565 − 1.LN849008, GCA_001014285 − 1.CP011414, GCA_001014305 − 1.CP011415, GCA_001015095 − 1.CP011534,
GCA_001017435 − 1.CP011371, GCA_001017575 − 1.CP011372, GCA_001017595 − 1.CP011538, GCA_001017615 − 1.CP011474,
GCA_001017635 − 1.CP011349 − CP011358, GCA_001017775 − 1.CP011568, GCA_001019635 − 1.CP007560, GCA_001019675 −
1.CP007561, GCA_001019695 − 1.CP007562, GCA_001020185 − 1.CP011535, GCA_001020205 − 1.CP011663, GCA_001020945 −
1.CP011342, GCA_001020955 − 1.CP011367, GCA_001020985 − 1.CP011542 − CP011544, GCA_001021005 − 1.CP011343,
GCA_001021025 − 1.CP011541, GCA_001021045 − 1.CP011545, GCA_001021065 − 1.CP011546, GCA_001021085 − 1.CP004021,
GCA_001021875 − 1.CP007670 − CP007671, GCA_001021895 − 1.CP007674 − CP007675, GCA_001021915 − 1.CP007810,
GCA_001021935 − 1.CP008714, GCA_001021955 − 1.CP008776, GCA_001021975 − 1.CP009928, GCA_001021995 − 1.CP011573 −
CP011574, GCA_001022015 − 1.CP011569 − CP011572, GCA_001022035 − 1.CP011575 − CP011578, GCA_001022055 −
1.CP011582 − CP011584, GCA_001022075 − 1.CP011579 − CP011581, GCA_001022095 − 1.CP011585 − CP011591, GCA_001022115 −
1.CP011613 − CP011618, GCA_001022135 − 1.CP011598 − CP011602, GCA_001022155 − 1.CP011603 − CP011612, GCA_001022175 −
1.CP011619 − CP011624, GCA_001022195 − 1.CP011625 − CP011636, GCA_001022215 − 1.CP011637 − CP011642, GCA_001022235 −
1.CP011643 − CP011647, GCA_001022255 − 1.CP011648 − CP011650, GCA_001022275 − 1.CP011651 − CP011657, GCA_001022295 −
1.CP011592 − CP011597, GCA_001023495 − 1.CP011068, GCA_001023535 − 1.CP011020, GCA_001023595 − 1.CP011686,
GCA_001025135 − 1.AP012323, GCA_001025155 − 1.AP012322, GCA_001025175 − 1.AP012324, GCA_001025195 − 1.AP012325,
GCA_001025215 − 1.AP012330, GCA_001026925 − 1.CP010867, GCA_001026945 − 1.CP010795, GCA_001026965 − 1.CP010779 −
CP010780, GCA_001026985 − 1.CP010423, GCA_001027025 − 1.CP011232, GCA_001027045 − 1.CP011528 − CP011529,
GCA_001027065 − 1.CP007689, GCA_001027085 − 1.CP007688, GCA_001027105 − 1.CP011526 − CP011527, GCA_001027125 −
1.CP007687, GCA_001027165 − 1.CP007686, GCA_001027205 − 1.CP007685, GCA_001027225 − 1.CP011511, GCA_001027245 −
1.CP007684, GCA_001027265 − 1.CP011501, GCA_001027285 − 1.CP011509, GCA_001028285 − 1.CP011827, GCA_001028625 −
1.CP011805, GCA_001028645 − 1.CP007601 − CP007602, GCA_001028665 − 1.CP011503 − CP011506, GCA_001028705 −
1.CP011770 − CP011772, GCA_001029105 − 1.CP011807 − CP011809, GCA_001029125 − 1.CP006636 − CP006642, GCA_001029145 −
1.CP009743 − CP009745, GCA_001029245 − 1.CP011855, GCA_001029265 − 1.CP011856, GCA_001029645 − 1.CP011798,
GCA_001029815 − 1.CP007667, GCA_001029835 − 1.CP007668, GCA_001037985 − 1.CP011882, GCA_001038625 − 1.CP011803 −
CP011804, GCA_001038645 − 1.CP011854, GCA_001039415 − 1.CP011938, GCA_001039495 − 1.CP011939, GCA_001040945 −
1.CP011930, GCA_001042405 − 1.AP014808, GCA_001042445 − 1.CP011926, GCA_001042525 − 1.CP011921, GCA_001042545 −
1.CP011922, GCA_001042565 − 1.CP011923, GCA_001042595 − 1.AP012326, GCA_001042615 − 1.AP012327, GCA_001042635 −
1.AP012331, GCA_001042655 − 1.AP012332, GCA_001042675 − 1.AP012333, GCA_001042695 − 1.AP012334, GCA_001042715 −
1.CP009687 − CP009688, GCA_001042735 − 1.CP011962 − CP011963, GCA_001042745 − 1.CP011955, GCA_001042775 −
1.CP011956, GCA_001042795 − 1.CP011957, GCA_001042815 − 1.CP011958, GCA_001042835 − 1.CP011959, GCA_001042855 −
1.CP011960, GCA_001042875 − 1.CP011961, GCA_001043135 − 1.CP011348, GCA_001043175 − 1.CP011494, GCA_001043215 −
1.CP011495 − CP011496, GCA_001043255 − 1.CP008744, GCA_001043295 − 1.CP008774 − CP008775, GCA_001045415 −
1.CP007653 − CP007655, GCA_001045685 − 1.CP012001, GCA_001046835 − 1.CP011024, GCA_001077655 − 1.CP012006 −

$CP012008$, $GCA\_001077675-1.CP012004-CP012005$, $GCA\_001078055-1.CP010777$
