# Supplementary material for: Intraspecies characterization of bacteria via evolutionary modeling of protein domains
Source: Sci Rep. 2022 Oct 5;12:16595. doi: 10.1038/s41598-022-21036-3 (PMC9534902; doi:10.1038/s41598-022-21036-3)
Supplement: Supplementary file 2 — Supplementary Information 2. [file 41598_2022_21036_MOESM2_ESM.pdf]

Supplementary Material

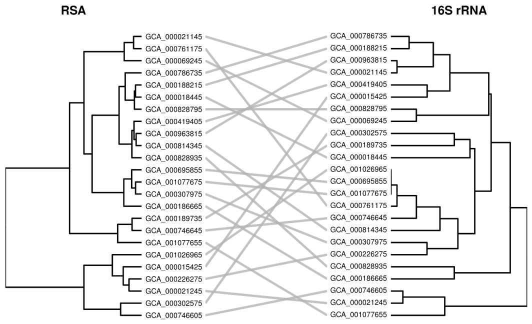

Strains of *Acinetobacter baumannii*

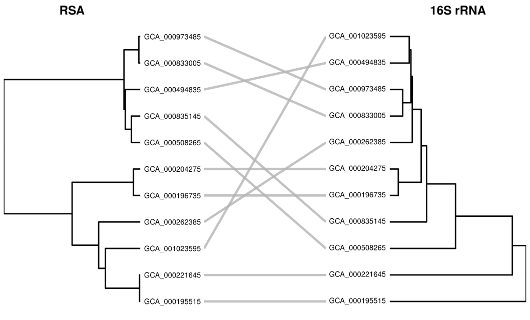

Strains of *Bacillus amyloliquefaciens*

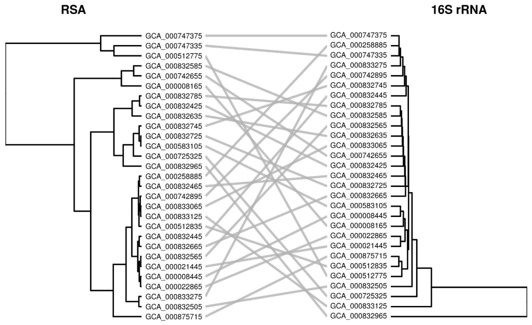

Strains of *Bacillus anthracis*

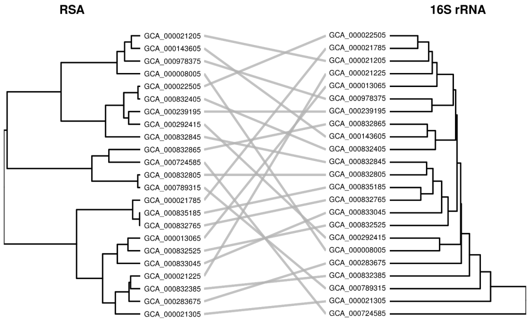

Strains of *Bacillus cereus*

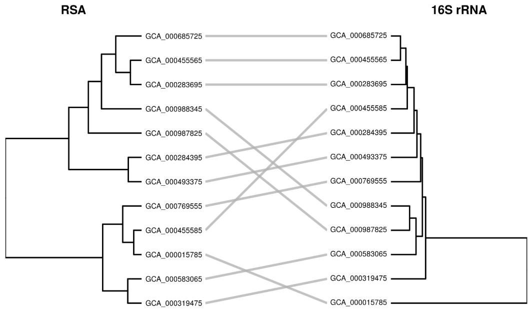

Strains of *Bacillus methylotrophicus*

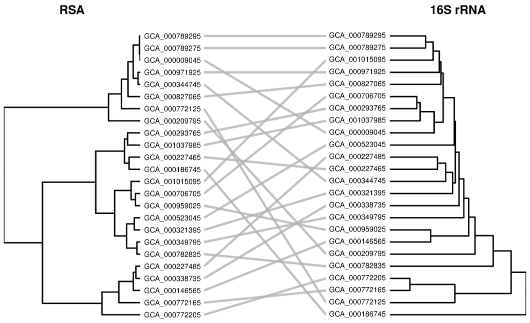

Strains of *Bacillus subtilis*

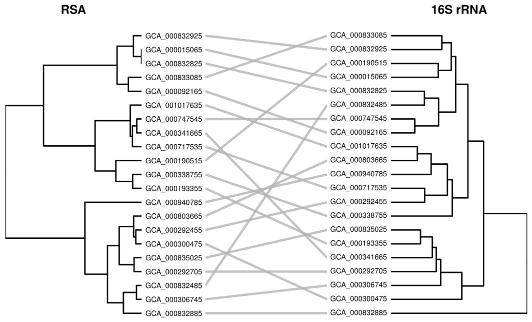

Strains of *Bacillus thuringiensis*

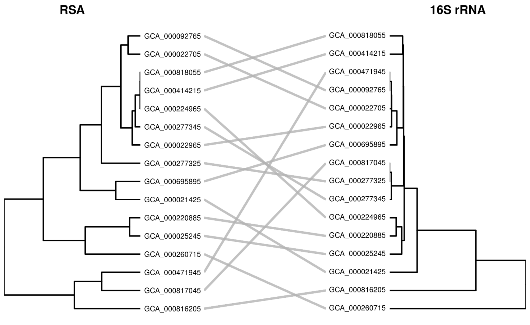

Strains of *Bifidobacterium animalis*

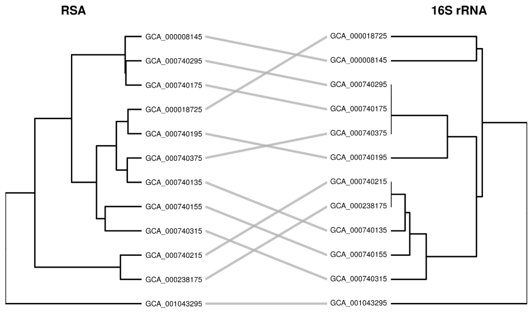

Strains of *Brucella abortus*

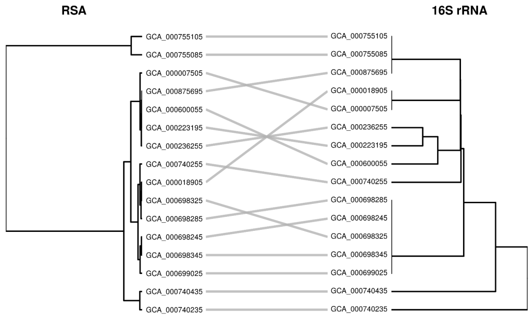

Strains of *Brucella suis*

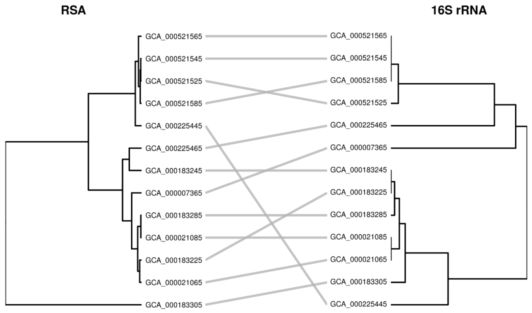

Strains of *Buchnera aphidicola*

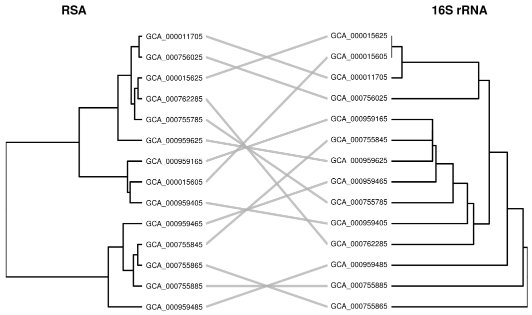

Strains of *Burkholderia mallei*

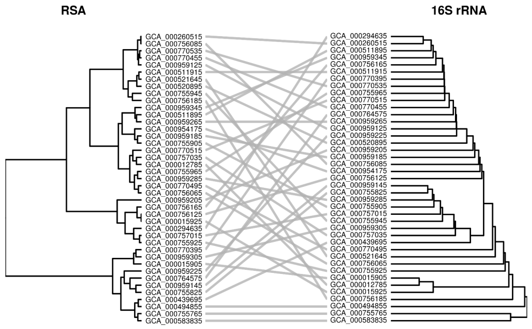

Strains of *Burkholderia pseudomallei*

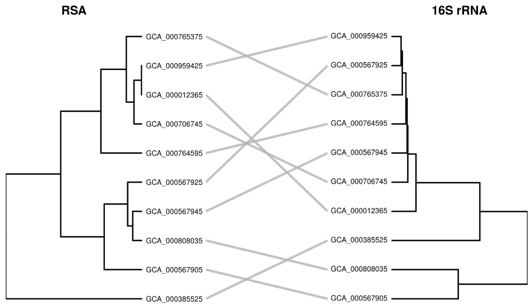

Strains of *Burkholderia thailandensis*

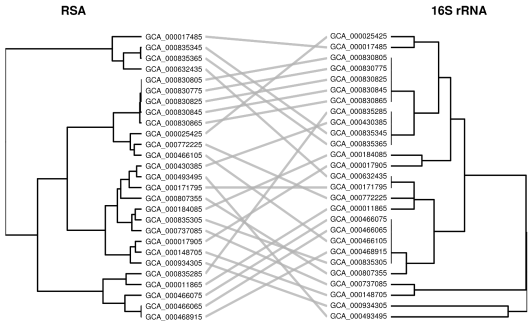

Strains of *Campylobacter jejuni*

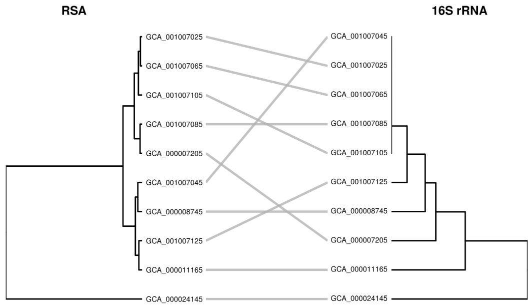

Strains of *Chlamydia pneumoniae*

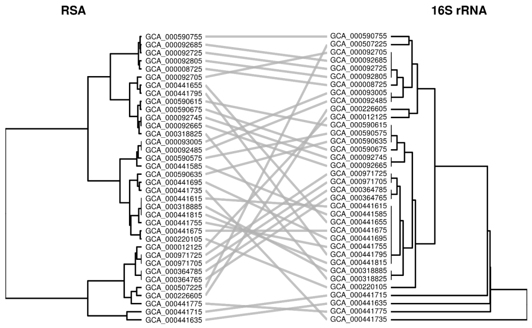

Strains of *Chlamydia trachomatis*

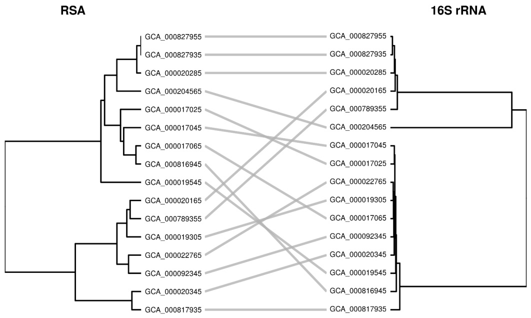

Strains of *Clostridium botulinum*

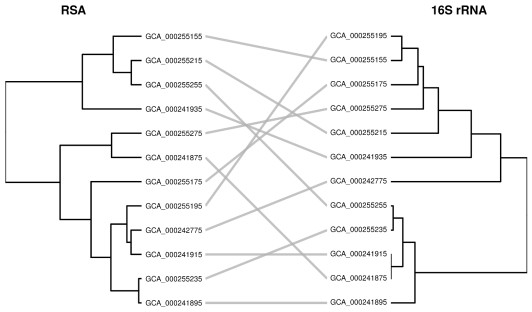

Strains of *Corynebacterium diphtheriae*

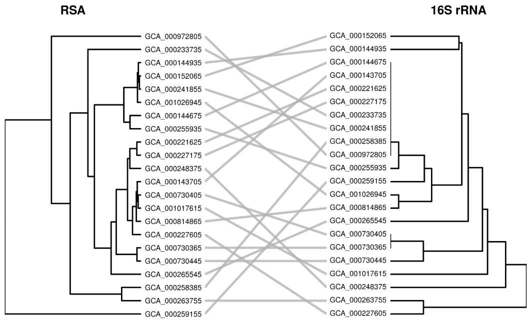

Strains of *Corynebacterium pseudotuberculosis*

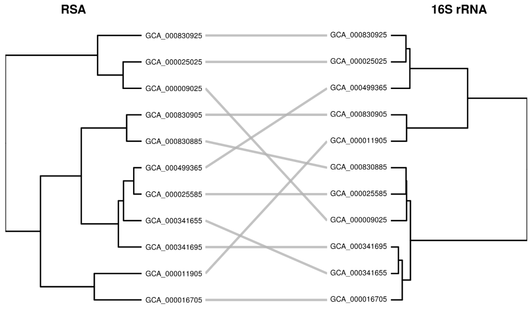

Strains of *Dehalococcoides mccartyi*

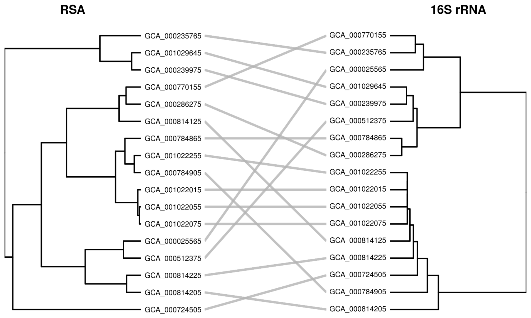

Strains of *Enterobacter cloacae*

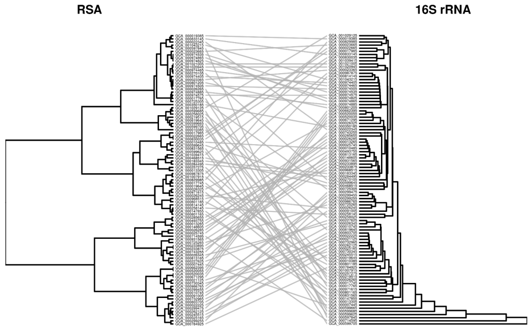

Strains of *Escherichia coli*

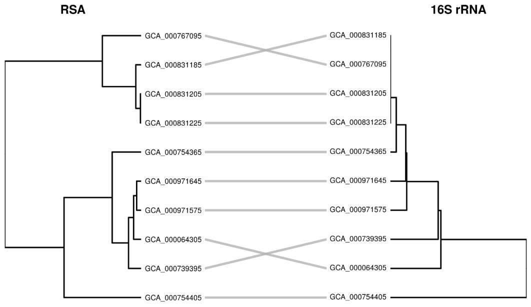

Strains of *Flavobacterium psychrophilum*

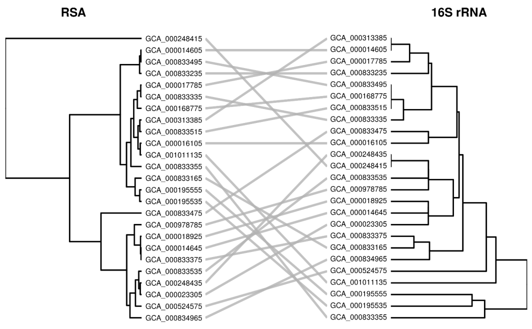

Strains of *Francisella tularensis*

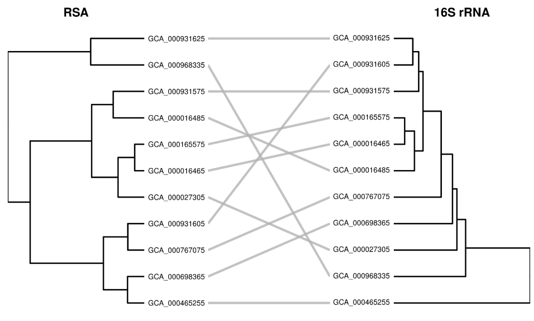

Strains of *Haemophilus influenzae*

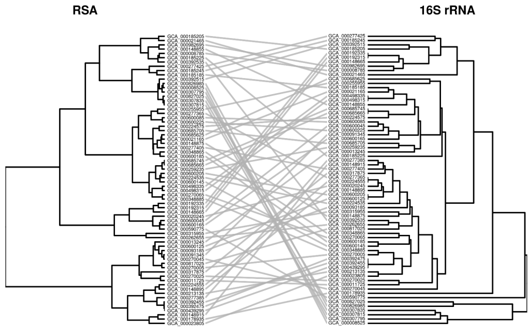

Strains of *Helicobacter pylori*

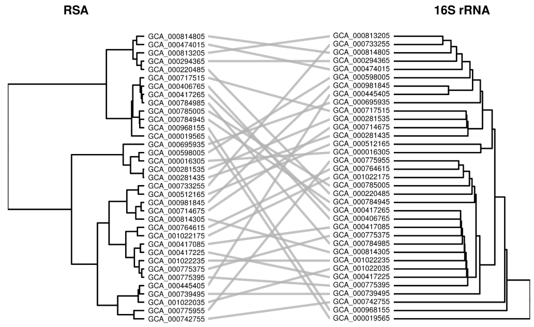

Strains of *Klebsiella pneumoniae*

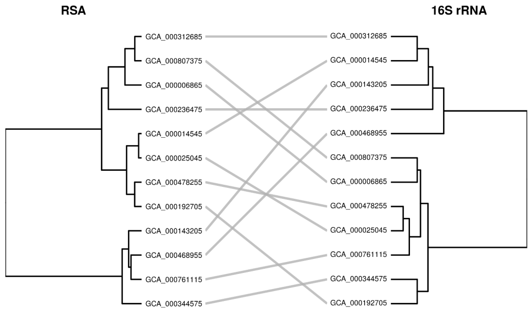

Strains of *Lactococcus lactis*

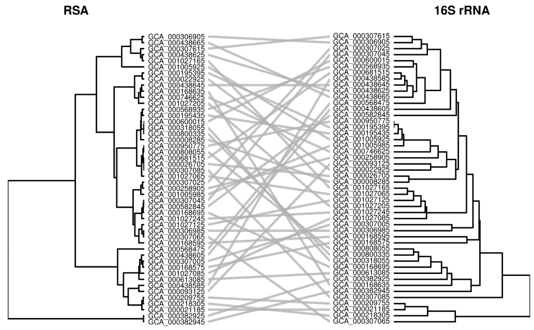

Strains of *Listeria monocytogenes*

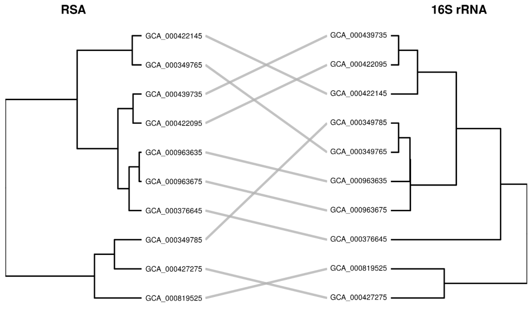

Strains of *Mannheimia haemolytica*

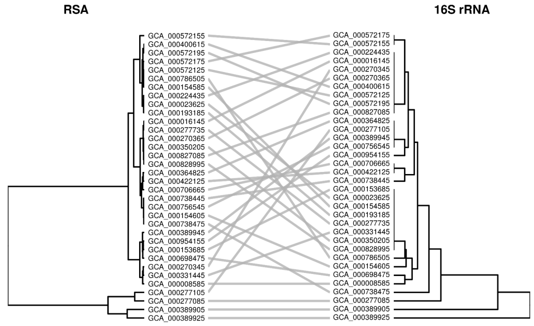

Strains of *Mycobacterium tuberculosis*

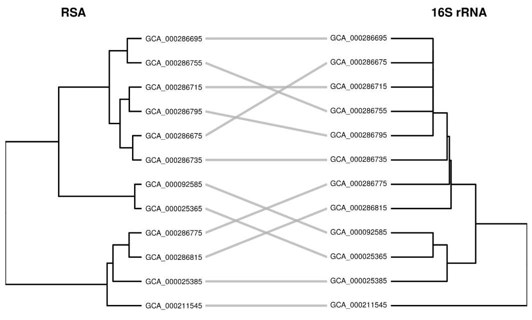

Strains of *Mycoplasma gallisepticum*

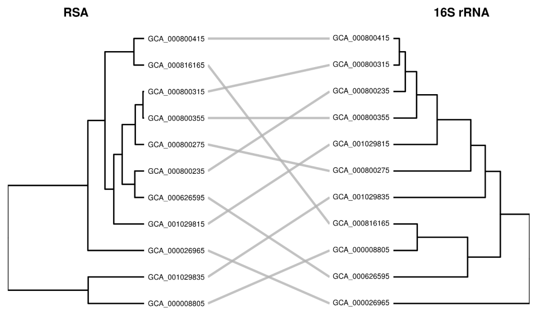

Strains of *Neisseria meningitidis*

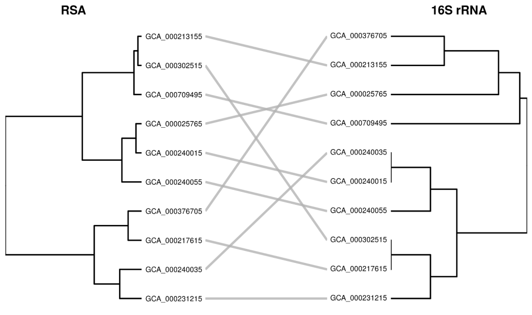

Strains of *Propionibacterium acnes*

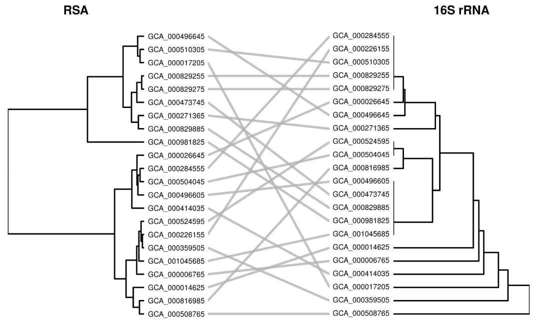

Strains of *Pseudomonas aeruginosa*

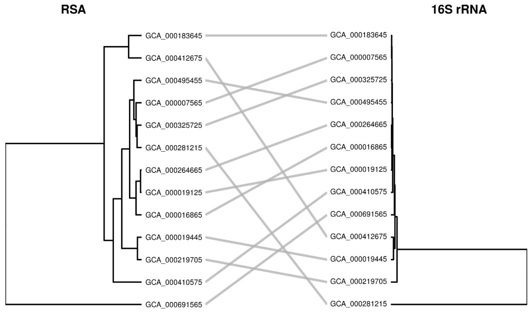

Strains of *Pseudomonas putida*

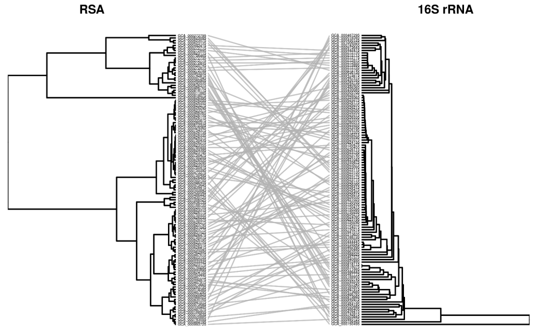

Strains of *Salmonella choleraesuis*

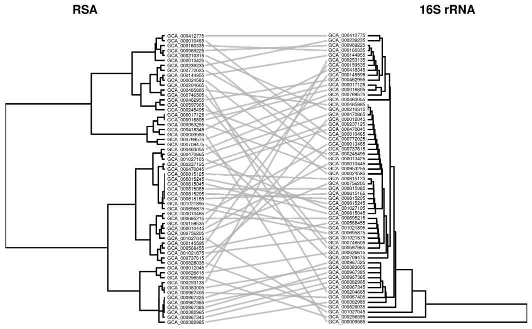

Strains of *Staphylococcus aureus*

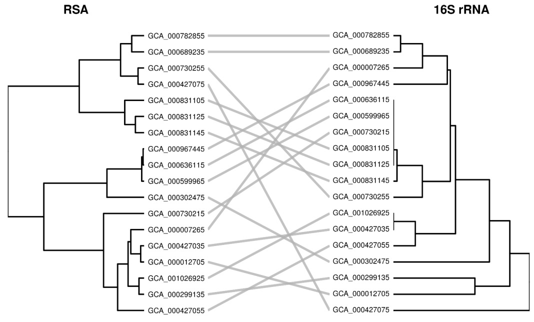

Strains of *Streptococcus agalactiae*

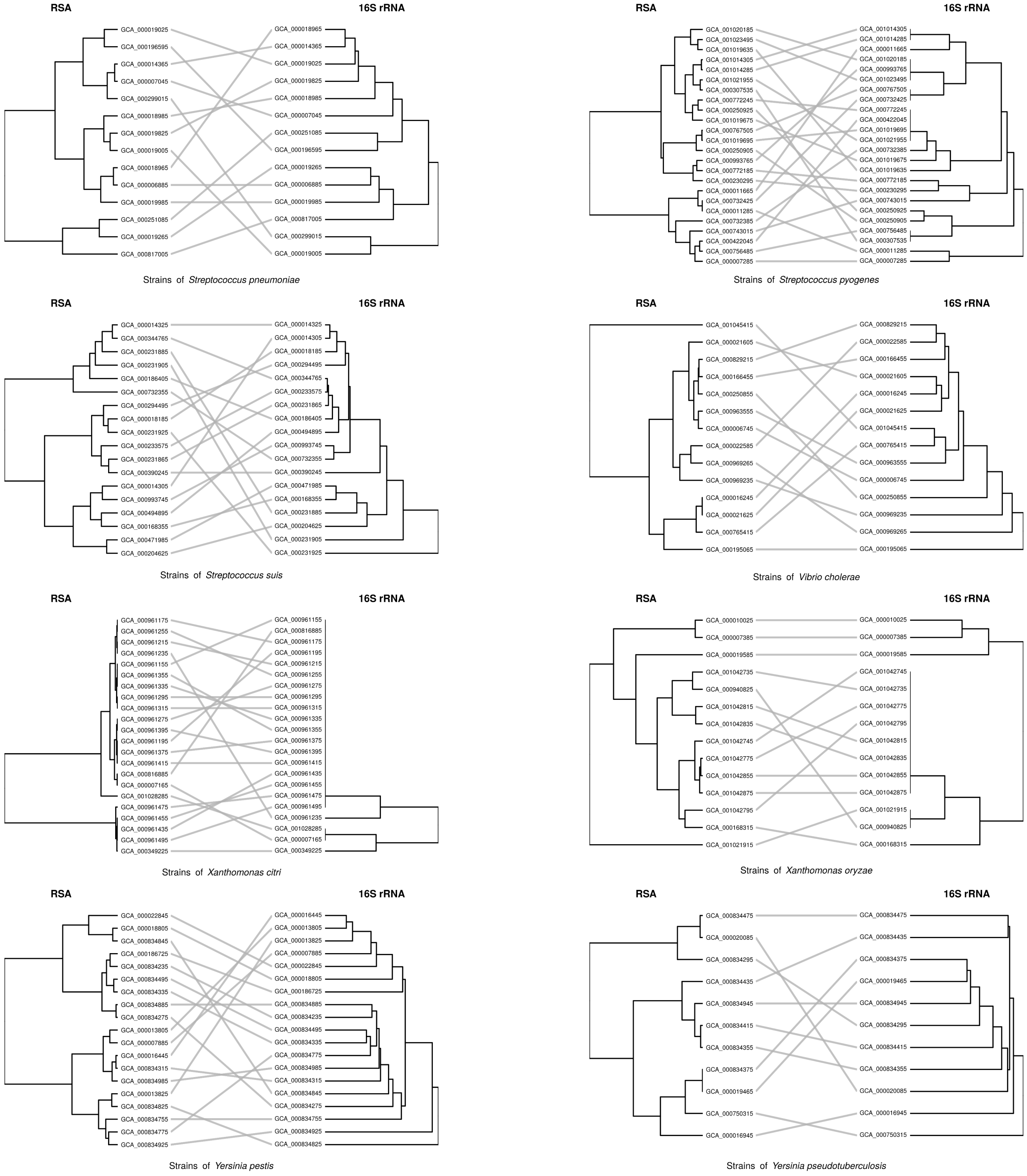

Figure S8. Hierarchical clustering of strains from all the species considered in the analysis.
